# The contributions of mitochondrial and nuclear mitochondrial genetic variation to neuroticism

**DOI:** 10.1038/s41467-023-38480-y

**Published:** 2023-05-30

**Authors:** Charley Xia, Sarah J. Pickett, David C. M. Liewald, Alexander Weiss, Gavin Hudson, W. David Hill

**Affiliations:** 1 Lothian Birth Cohort studies, University of Edinburgh, 7 George Square, Edinburgh, EH8 9JZ UK; 2 Department of Psychology, School of Philosophy, Psychology and Language Sciences, University of Edinburgh, 7 George Square, Edinburgh, EH8 9JZ UK; 3 Wellcome Centre for Mitochondrial Research and Translational and Clinical Research Institute, The Medical School, Newcastle University, Newcastle upon Tyne, NE2 4HH UK; 4 Wellcome Centre for Mitochondrial Research and Biosciences Institute, Newcastle University, Newcastle upon Tyne, NE2 4HH UK

**Keywords:** Genetics, Neuroscience, Psychology, Heritable quantitative trait, Risk factors

## Abstract

Neuroticism is a heritable trait composed of separate facets, each conferring different levels of protection or risk, to health. By examining mitochondrial DNA in 269,506 individuals, we show mitochondrial haplogroups explain 0.07-0.01% of variance in neuroticism and identify five haplogroup and 15 mitochondria-marker associations across a general factor of neuroticism, and two special factors of anxiety/tension, and worry/vulnerability with effect sizes of the same magnitude as autosomal variants. Within-haplogroup genome-wide association studies identified H-haplogroup-specific autosomal effects explaining 1.4% variance of worry/vulnerability. These H-haplogroup-specific autosomal effects show a pleiotropic relationship with cognitive, physical and mental health that differs from that found when assessing autosomal effects across haplogroups. We identify interactions between chromosome 9 regions and mitochondrial haplogroups at *P* < 5 × 10^−8^, revealing associations between general neuroticism and anxiety/tension with brain-specific gene co-expression networks. These results indicate that the mitochondrial genome contributes toward neuroticism and the autosomal links between neuroticism and health.

## Abstract (web summary)

Genetic variation in mitochondrial DNA can influence human traits, but it is not as well studied in association with complex traits. Here, the authors find associations between mitochondrial haplogroups and genetic variation and neuroticism.

## Introduction

Neuroticism is one of the five higher-order factors of personality and is measured using items that refer to irritability, anger, sadness, anxiety, worry, hostility, self-consciousness, and vulnerability^1,2^. Individual differences in neuroticism are relatively stable across the adult life course^3,4^.

Individuals with a higher level of neuroticism are more likely to experience stress, and to be less happy and satisfied with their lives than those with a lower level^5,6^. Neuroticism has also been linked to socioeconomic position, with higher levels of neuroticism associated with lower levels of education and income^7^. Furthermore, individuals with higher levels of neuroticism are more likely to be diagnosed with psychiatric disorders, suffer from neurodegenerative conditions, such as Alzheimer’s disease and Parkinson’s disease^8–10^, have poorer physical health^11^, and die at a younger age^12^. In addition to the burden it can place on the individual, neuroticism has been estimated to place an economic burden on society that is greater than substance abuse, mood disorders, or anxiety disorders^13^ making the identification of its causes a matter of public health.

Several studies have however, reported that higher neuroticism can be beneficial to health^14^ and mortality^15^. These paradoxical findings were explained by the observation that, using a bi-factor model, three factors can be extracted from the Short-scale Eysenck Personality Questionnaire-Revised^16^ neuroticism scale, and that these factors had different effects on health. The first factor, a general factor, reflected a common source of variance across all 12 items. The second and third factors reflected two common sources of variance left over following the extraction of the general factor (the residual variance). These factors—worry/vulnerability and anxiety/tension—are independent of the general factor of neuroticism, and each explained the common remaining variance of a subset of the 12 items. General neuroticism was associated with poorer physical health whereas worry/vulnerability and anxiety/tension are linked to better physical health^17–19^. Importantly, all three are associated with worse mental health^19^.

As with other quantitative traits such as height and intelligence, genetic variation is associated with phenotypic variation in the general factor of neuroticism ${h}_{{snp}}^{2}$ = 10.7%, *se* = 0.49%) and the special factors of worry/vulnerability (${h}_{{snp}}^{2}$ = 6.4%, *se* = 0.28%), and anxiety/tension (${h}_{{snp}}^{2}$ = 5.7%, *se* = 0.32%)^19^. Large scale genetic analyses performed on these three neuroticism phenotypes has indicated a different underlying genetic architecture. For example, whilst each of the three showed associations with genes whose proteins are expressed in the brain, the general factor of neuroticism was associated with genetic variation in genes linked to neurogenesis and the formation of dendrites, whereas the worry/vulnerability factor was associated with genetic variation in genes linked to high-voltage-gated calcium ion channels and the voltage-gated calcium channel complex. Consistent with the reported phenotypic observations^17,18^, genetic correlations between the three factors of neuroticism and health variables also showed differing directions of effect with physical health and mortality. For the general factor of neuroticism negative genetic correlations with self-rated health, and longevity were observed along with positive genetic correlations with coronary artery disease. Whereas, for the two special factors, the direction of these genetic correlations was reversed, consistent with them having a protective effect on health.

Large scale genetic analysis have been performed for these factors of neuroticism^19^, but so far no investigation has examined the contributions of mitochondrial DNA. However, three lines of evidence suggest that mitochondrial variation may be associated with variation in neuroticism. First, GWAS performed on the general factor of neuroticism and the anxiety/tension and worry vulnerability special factors identified genome-wide significant loci linked with autosomal mitochondrial genes. Second, genome-wide significant loci identified in GWAS of neuroticism contain genes and loci that are involved in the expression of the tissues of the brain. As mitochondria are primarily responsible for the production of the chemical energy required for metabolism, and, due to the high energy requirements of the brain^20^, this link between neuroticism and the brain may extend to its source of energy. Third, neuroticism is predictive of Alzheimer’s disease^21^, and mtDNA variation has been shown to be associated with neurodegenerative disorders, including Alzheimer’s disease^22,23^ and Parkinson’s disease^24,25^. This link between neuroticism and both Parkinson’s disease and Alzheimer’s disease may be in part due to pleiotropic variation in the mitochondrial genome.

In the current study, we used molecular genetic data in 269,506 participants of UK Biobank to examine the contributions made to the general factor of neuroticism and the two special factors of anxiety/tension and worry/vulnerability by mitochondrial DNA. To do so, we first performed an association analysis of mitochondrial haplogroup and single variant analysis on each of the three factors of neuroticism. Second, we examine nuclear encoded mitochondrial genes as well as assessing the role that ‘mitonuclear’ combinations play in the phenotypic variance observed in the neuroticism factors using a test of enrichment. Third, we examine if the association between the neuroticism factors with nuclear DNA varies by mitochondrial haplogroup.

## Results

### MT-DNA association analysis

Following quality control (QC) (Supplementary Fig. 1) we explored the associations between haplogroup and mtDNA variation with the three neuroticism factors using an alpha level of 0.005 for haplogroup analysis and 0.001 for MT-GWAS analysis.

The total effect of haplogroup on the general factor of neuroticism was small but significant (*R*^2^ = 0.0001, *P* = 0.001). This effect was consistent with the size of the mitochondrial genome when compared to the effects associated with common autosomal SNP variation (Supplementary Fig. 2 and Supplementary Data 1).

**Fig. 1:**
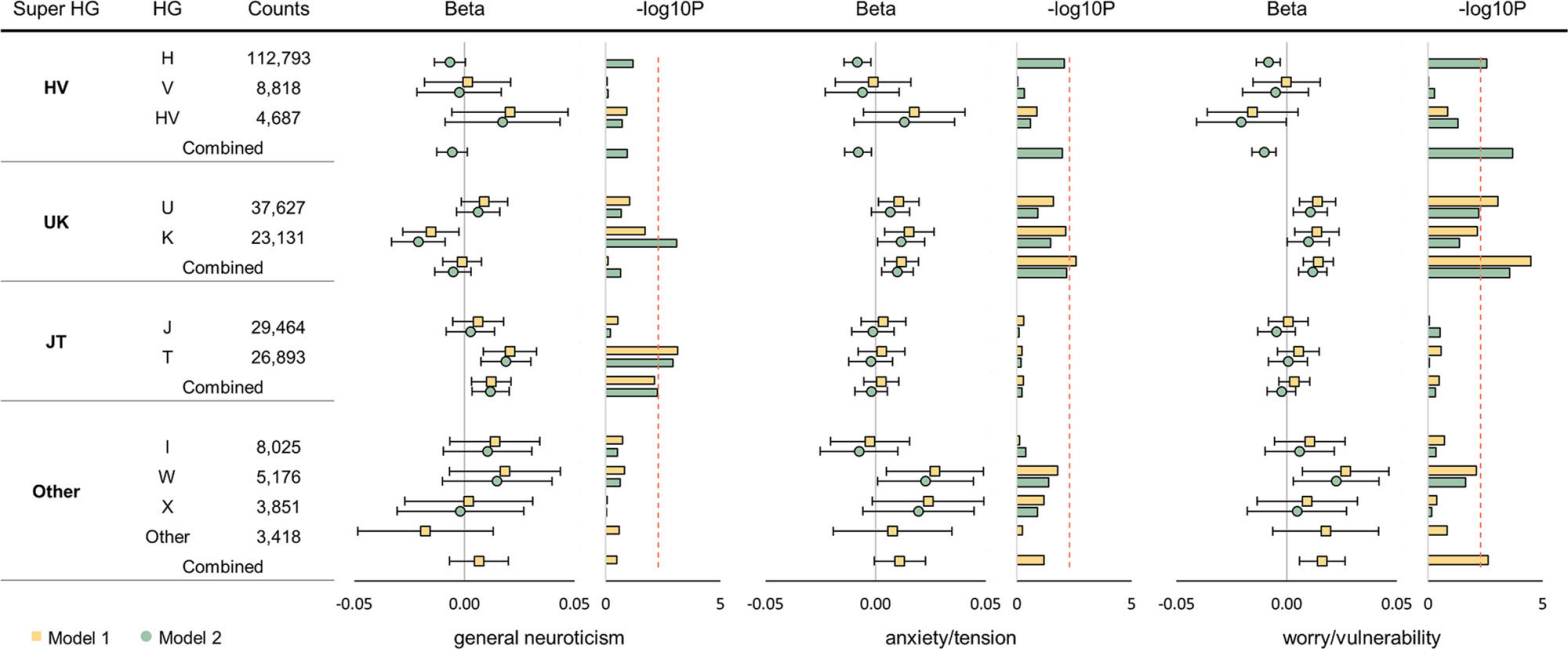
Phenotype-haplogroup association results in 263,883 unrelated white British samples in UK Biobank. Counts pertains to the number of individuals that belong to each haplogroup (HG). Model 1, compares the mean of each group to the mean of the reference group. Haplogroup H and super haplogroup **HV** were used as the reference groups in haplogroup association analysis and super haplogroup association analysis, respectively. Model 2, compares the mean of each haplogroup/super haplogroup to the mean of those from outside that grouping. The rows labelled combined illustrates the association with the three neuroticism phenotypes following the combining of relevant haplogroups and the testing of the super haplogroup. Group ‘Other’ was not tested in model 2 as it was a mixture of haplogroups and was not of interest. Error bars show the 95% confidence interval. The significance threshold (red dotted line) for the ten common haplogroups was 0.05/10. For the super haplogroups, we considered them to be associated with the neuroticism phenotypes if the *P* value (two-sided *t* test) of super haplogroup analysis was lower than the threshold of 0.05/10 and lower than any of their contributing haplogroups.

**Fig. 2:**
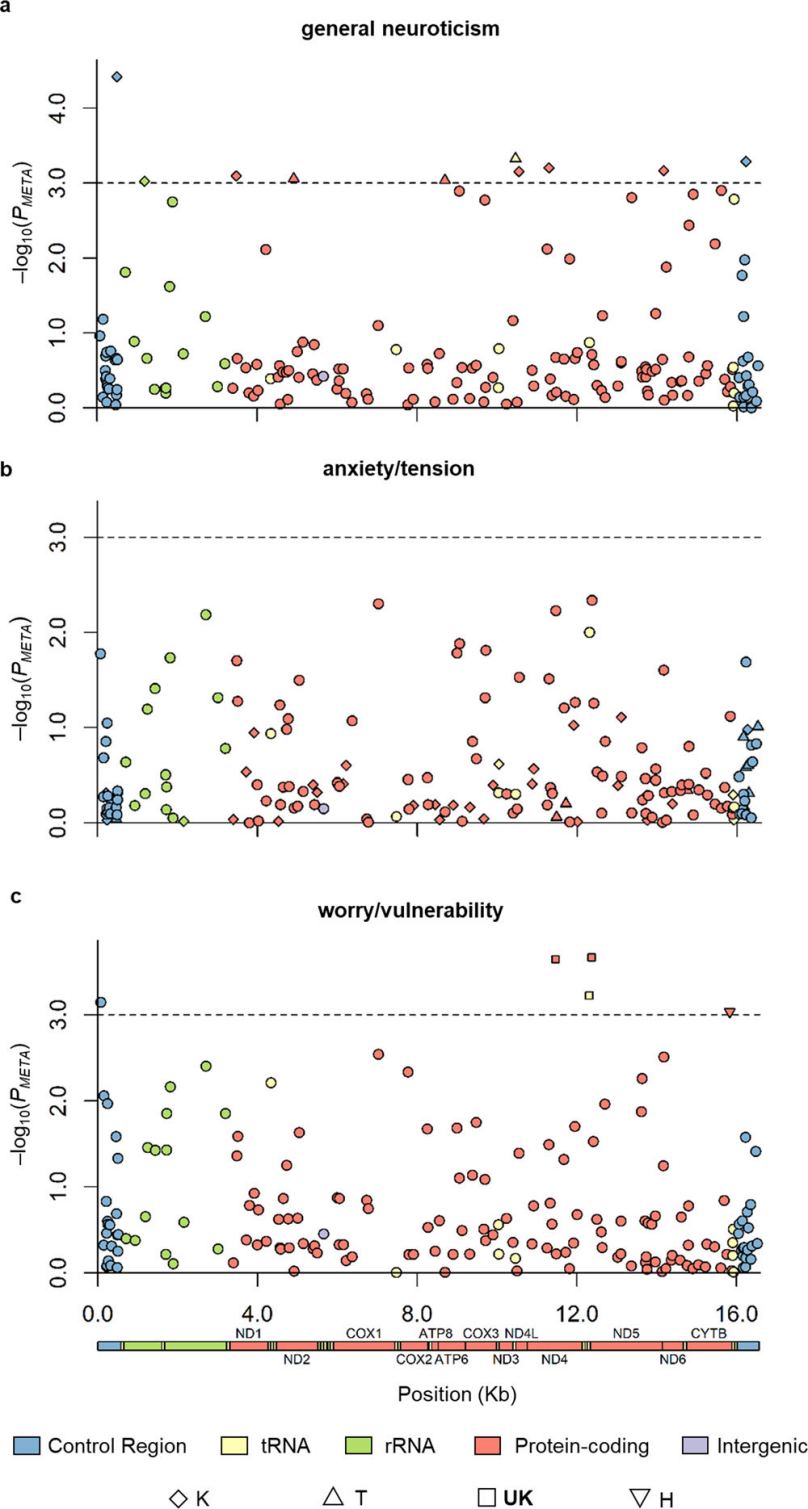
MT-GWAS of neuroticism factors in 269,506 unrelated white British samples in UK Biobank. Maximum sample sizes were 269,506. **a**–**c** present MT-GWAS Manhattan plot of general neuroticism, anxiety/tension, and worry/vulnerability, respectively, *Y* axis: −log10 *P* values of the meta-analysis (two-sided *t* test). Markers are coloured based on their location in the MT genome (*X* axis). Dotted line: −log10 (0.001), significance level. For significant associations, markers were shaped if they were haplogroup-associated markers, e.g., haplogroup-defining markers.

Haplogroup K was associated with a lower level of general neuroticism (Model 2: *N* = 263,883, $\beta=\,-0.021$, ${se}=0.006$, $P=8.19\times {10}^{-4}$) whereas haplogroup T was associated with greater levels (Model 1: *N* = 263,883, $\beta=\,0.021$, ${se}=0.006$, $P=7.29\times {10}^{-4}$; Model 2: *N* = 263,883, $\beta=\,0.019$, ${se}=0.006$, $P=1.17\times {10}^{-3}$) (Fig. 1 and Supplementary Data 2, 3). This is supported by our analysis at the variant level, which identified 10 mtSNPs that reached the significance threshold: seven were associated with haplogroup K and three were associated with haplogroup T (Fig. 2, Supplementary Fig. 3 and Supplementary Data 4). The lead haplogroup K marker was m.497 C > T (rs28660704; *N* = 269,506, $\beta=\,-0.034$, ${se}=0.008$, $P=3.82\times {10}^{-5}$), which is in the control region (*MT-CR*) where the MT *D-loop* is located). Marker m.497 C > T defines the K1a haplogroup and has a frequency of 55.72% in K haplogroup samples and 4.81% in the QCed UK Biobank dataset (correlation with K haplogroup, ${r}^{2}=0.53$). Simulation indicated that the observed haplogroup K associations (i.e., both haplogroup and marker associations) are likely driven by m.497 C > T or other variants in linkage disequilibrium (LD) with m.497 C > T that were not present in the data (Supplementary Fig. 4).

The lead haplogroup T marker was m.10463 T > C (rs28358279) in *MT-TR* (*N* = 269,446, $\beta=\,0.020$, ${se}=0.006,\, P=4.72\times {10}^{-4}$), which is a haplogroup T defining marker with a frequency of 10.63% in the QCed UK Biobank dataset.

Regarding the special factor anxiety/tension, there was a small but significant total effect of haplogroup as estimated using regression (*R*^2^ = 7.27 × 10^−5^, *P* = 0.038). The magnitude of these effects were as predicted by the size of the mitochondrial genome compared to the autosomes (Supplementary Fig. 2 and Supplementary Data 1). We found associations between super haplogroup **UK** with higher anxiety/tension (Model 1: *N* = 263,883, $\beta=0.017$, ${se}=0.004$, $P=0.003$) (Fig. 1), but no marker was associated with anxiety/tension at $P < 0.001$ (Fig. 2, Supplementary Data 2 and Supplementary Data 5).

For worry/vulnerability, the total effect of haplogroup was estimated to be proportional to its size where a small but significant (*R*^2^ = 0.0001, *P* = 0.002) effect was found (Supplementary Fig. 2 and Supplementary Data 1). Super haplogroup **HV** showed association with lower levels (Model 2: *N* = 263,883, $\beta=\,-0.010$, ${se}=0.003$, $P=2.03\times {10}^{-4}$) whereas super haplogroup **UK** was associated with higher worry/vulnerability (Model 1: *N* = 263,883, $\beta=\,0.015$, ${se}=0.004$, $P=3.32\times {10}^{-5}$; Model 2: $\beta=\,0.012$, ${se}=0.003$, $P=2.77\times {10}^{-4}$) (Fig. 1 and Supplementary Data 2, 3). MT-GWAS identified five significant associations, including three markers from super haplogroup **UK**, m.73 A > G, and m.15833 C > T (Fig. 2 and Supplementary Data 6). The lead **UK** marker was m.12372 G > A (rs2853499) in *MT-ND5* (*N* = 269,478, $\beta=\,0.012$, ${se}=0.003$, $P=2.14\times {10}^{-4}$) which has a frequency of 22.58% in the QCed UK Biobank dataset. m.73 A > G (rs869183622), which was associated with lower levels of worry/vulnerability (*N* = 269,499, $\beta=\,-0.009$, ${se}=0.003$, $P=7.15\times {10}^{-4}$), is a homoplastic marker in the *MT-CR* that is spread widely across a few haplogroups including super haplogroup **HV** (Supplementary Fig. 3) (frequency 45.01% in the QCed UK Biobank dataset). In our data, the majority of samples (95.5%) carrying m.73 A > G belonged to super haplogroup **HV**, creating a high correlation between m.73 A > G and super haplogroup **HV** (${r}^{2}=0.84$). Simulation shows the association of m.73 A > G and super haplogroup **HV** with worry/vulnerability is driven by the same underlying signal (Supplementary Fig. 4). The **HV** association was stronger than that for m.73 A > G, suggesting that the association is likely driven by super haplogroup **HV** or other variants in LD that were absent these data. Note, data for the super haplogroup **HV** defining marker m.14766 C > T is only available in the UKBL subset (*N* = 29,690, $\beta=-\!0.002$, ${se}=0.008$, $P=0.82$), and thus remained undetected likely due to statistical power. Finally, marker m.15833 C > T (rs41504845 in *MT-CYB*, *N* = 269,488, $\beta=-\!0.033$, ${se}=0.010$, $P=9.23\times {10}^{-4}$) defines H5a1, (present at 1.97% in our QCed UK Biobank dataset) and only appears in haplogroup H (frequency 4.71%) and haplogroup J (frequency 0.003%). Simulation suggests that m.15833 C > T and super haplogroup **HV** contribute two independent association signals (Supplementary Fig. 4).

Full results for haplogroup analysis and MT-GWAS can be found in Supplementary Data 2–6. No evidence was found that indicated the associations between haplogroup and MT-DNA marker presented above were due to a correlation between nuclear and mitochondrial DNA (see co-segregation analysis in Supplementary Notes).

### Pleiotropic mtDNA associations

A comparison of the associations identified with the three neuroticism variables with the 896 traits examined by Yonova-Doing et al.^26^ showed for worry/vulnerability the lead mtSNP association markers of m.12372 G > A and m.73 A > G were also associated with nine and eleven blood traits respectively (Supplementary Data 7). Of note is the finding that the direction of association was often in the opposite direction when comparing between worry/vulnerability and the blood traits identified. For example, at m.12372 A > G a positive association was detected for worry/vulnerability, but a negative association was found for red cell distribution width (RDW), white blood cell count (WBC), creatinine, and lymphocyte count (Supplementary Data 7). This finding is suggestive of a negative phenotypic correlation between worry/vulnerability and these blood traits. Similar effects were also identified considering the associated haplogroups and their defining markers, but with the additional association of haplogroup K with both the general factor of neuroticism and lymphocyte count (Supplementary Data 8). No mtSNPs that were associated with the general factor of neuroticism were associated with other traits.

The presence of a negative correlation between worry/vulnerability and WBC, RDW, and lymphocyte count was confirmed by examining their phenotypic and genetic correlations (Phenotypic correlations, WBC; *r* = −0.04, *se* = 0.002, *P* = 9.97 × 10^−105^, *r*_*g*_ = −0.11, *se* = 0.03, *P* = 3.42 × 10^−5^, RDW; *r* = −0.01, *se* = 0.002, *P* = 1.81 × 10^−11^, *r*_*g*_ = −0.07, *se* = 0.03, *P* = 0.01, LYMPH; *r* = −0.001, *se* = 0.002, *P* = 3.65 × 10^−6^, *r*_*g*_ = −0.10, *se* = 0.03, *P* = 9.83 × 10^−6^). This directional consistency confirmed that the effect of m.73 A > G and m.12372 G > A on worry/vulnerability and blood traits was consistent with the broader relationship between these traits. A positive phenotypic and genetic correlation was identified between the general factor of neuroticism and lymphocyte count (*r* = 0.01, *se* = 0.002, *P* = 2.21 × 10^−8^, *r*_*g*_ = 0.05, *se* = 0.02, *P* = 4.80 × 10^−3^) consistent with their relationship as predicted by the effect of the K haplogroup (Supplementary Data 9). These differences in the direction of association of RDW, WBC, and lymphocyte count with general neuroticism and worry/vulnerability highlight the different links to biology and health the factors of neuroticism have^19,27^ and show that these links are, in part, likely due to their links with genetic variation of the mitochondrial genome.

### MT-nDNA gene analysis

Using published data on the three factors of neuroticism^19^ a gene-based analysis conducted on 2,099 MT-nDNA genes, also known as N-mt genes (Methods) yielded 54 associations from 48 unique genes at *P* < 0.05/2,099 = 2.38 × 10^−5^. Forty-four were associated with only one neuroticism trait: 25 with the general factor of neuroticism, 13 with anxiety/tension, and 6 with worry/vulnerability (Supplementary Data 10).

We annotated the genes that were associated with each of the three neuroticism traits using FUMA^28^ by fitting all 2,220 MT-nDNA candidate genes as background genes (Supplementary Data 11). For the general factor of neuroticism, two terms (apart from neuroticism itself and items that contributed to it) were significantly associated after correcting for multiple test. These were GWAScatalog: regular attendance at a religious group (*q* = 0.005) and GWAScatalog: (short) sleep duration (*q* = 0.006). Regarding anxiety/tension, three enriched terms were found - GWAScatalog: hypertension (*q* = 0.04), GWAScatalog: autism spectrum disorder or schizophrenia (*q* = 0.04) and position: chr6p21 (*q* = 0.01) (HLA region). No enrichment was found for worry/vulnerability.

Finally, a gene-set analysis using a competitive test of enrichment was performed to examine whether the whole set of 2,099 MT-nDNA candidate genes or its annotated subsets (e.g., gene subsets based on different mitochondrial pathways) were enriched for each of the three neuroticism phenotypes. No enrichment was found in either the whole set or its annotated subsets at *q* < 0.05 (Supplementary Data 12).

### MT-haplogroup-stratified nGWAS and MT-nDNA interaction

Cells harbouring different MT haplogroups are known to have functional differences in mtDNA copy number, gene expression, bioenergetics, etc., and are related to immunity and diseases like type 2 diabetes^29–34^ and that specific combinations of mtDNA/nDNA alleles can modulate disease risk^35,36^. We hypothesised that nuclear DNA associations with neuroticism may differ by MT haplogroup and examined genome-wide, gene-based, and SNP-based MT-nDNA interactions acting on our three neuroticism phenotypes. The significance threshold for each analysis was set to 0.05/10, 0.05/18,337, and 5 × 10^−8^, respectively.

MT-haplogroup-stratified GWAS was performed on nuclear SNPs for the ten common MT-haplogroups. Next, using LDSC, we estimated the genetic correlation between each of the ten haplogroups with the remaining nine haplogroups that were meta-analysed (Methods). The genetic correlation between worry/vulnerability in H-haplogroup-stratified GWAS (*h*^*2*^ = 0.074, *se* = 0.005, *P* = 2.41 × 10^−53^) and non-H-haplogroup-stratified GWAS (*h*^*2*^ = 0.065, *se* = 0.004, *P* = 4.24 × 10^−56^) was significantly lower than 1 (*r*_*g*_ = 0.865, *se* = 0.046, *P* = 3.65 × 10^−3^), suggesting that nuclear DNA contributions to worry/vulnerability differ by MT-haplogroup (Supplementary Data 13). To identify H-haplogroup-specific nuclear SNP-association and remove the common genetic factors shared between H and non-H haplogroup-stratified GWAS of worry/vulnerability, mtCOJO was used (Methods). The heritability of worry/vulnerability that was unique to the H haplogroup was *h*^*2*^ = 0.014, *se* = 0.004, *P* = 4.43 × 10^−4^. No SNPs (*P* < 5 × 10^−8^) or gene-based tests of H-haplogroup specific association (*P* < 0.05/18,337) attained statistical significance.

**Fig. 3:**
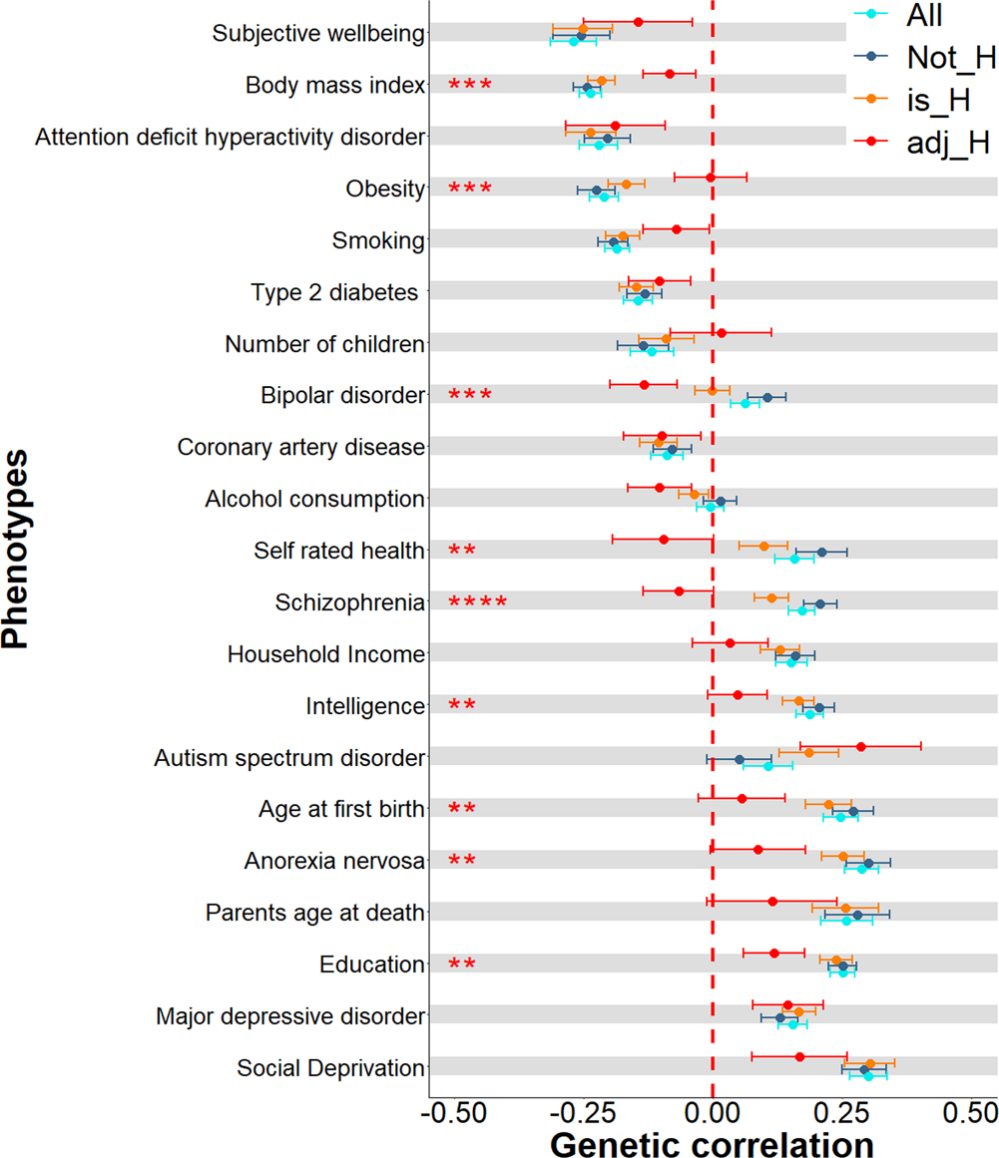
Comparing genetic correlations derived using H (*N* = 112,793) and non-H-haplogroup (*N* = 151,090) stratified worry/vulnerability with 21 traits. All refers to previous publications on worry/vulnerability^19^ where all haplogroups were combined into a single GWAS (*N* = 270,059). *NonH HG* indicates that the GWAS was conducted only in those individuals (*N* = 151,090) who were not carriers of the H haplogroup, whereas *H HG* indicates that the GWAS was conducted on those (*N* = 112,793) who were carriers of the H-haplogroup. *H HG Uni* indicates that the GWAS on worry/vulnerability conducted on H haplogroup individuals was conditioned on the *Non-H HG* group in order to examine the contributions of the H haplogroup without the shared effects found between H and Non H haplogroups. Error bars show one standard error. Asterisks indicate the number of significantly different genetic correlations at *P* < 0.05 (two-sided Z test derived using (2*pnorm(−abs(abs(*r*_*g*_i–*r*_*g*_j)/sqrt(SEi 2 + SEj 2))))) per trait. Full results can be found in Supplementary Data 14.

Performing genetic correlation analysis in LDSC, significantly different genetic correlations were found for nine traits (Fig. 3 and Supplementary Data 14) comparing between H and non-H-haplogroup GWAS. For example, bipolar disorder showed a significant positive genetic correlation with worry/vulnerability in the non-H-haplogroup (*r*_*g*_ = 0.104, *se* = 0.037, *P* = 0.005) and a significantly smaller genetic correlation of around zero in the H-haplogroup (*r*_*g*_ = −0.001, *se* = 0.033, *P* = 0.974, *P*_diff_ = 0.035). However, following adjustment using mtCOJO to control for the overlap with the non-H-haplogroup, the genetic correlation became negative and attained nominal statistical significance (adjusted-H-haplogroup, *r*_*g*_ = −0.134, *se* = 0.065, *P* = 0.040). This effect did not withstand correction for multiple comparisons, but the difference was statistically significant (non-H-haplogroup _vs_ adjusted H-haplogroup *P*_diff_ = 0.002).

Next, we performed a gene-based association analysis using MT-haplogroup-stratified GWAS using MAGMA for each trait. These results were compared with previously published work^19^, which was conducted in all samples without stratification by mitochondrial haplogroup (Methods). We found one gene showing no association when all haplogroups were included, but that was significantly associated with the general factor of neuroticism when the haplogroup V was analysed separately (electron transfer flavoprotein subunit alpha, *ETFA*, with general neuroticism, *P* = 0.21 in the full set with *N* = 270,059 vs. *P* = 4.48 × 10^−7^ in haplogroup-V-stratified GWAS with *N* = 8,818), and three genes that were less significant in the full set compared to the haplogroup-stratified results (glutamate ionotropic receptor kainate type subunit 3, *GRIK3*, with general neuroticism, *P* = 5.50 × 10^−5^ in the full set vs. *P* = 2.36 × 10^−6^ in H-haplogroup-stratified GWAS with *N* = 112,792; defensin beta 132, *DEFB132*, with anxiety/tension, *P* = 7.14 × 10^−4^ in the full set vs. *P* = 6.50 × 10^−7^ in T-haplogroup-stratified GWAS with *N* = 26,893; propionyl-CoA carboxylase subunit beta, *PCCB*, with worry/vulnerability, *P* = 2.40 × 10^−5^ in the full set vs. *P* = 1.46 × 10^−6^ in K-haplogroup-stratified GWAS with *N* = 23,131) (Supplementary Data 15). *PCCB* and *ETFA* are both MT-nDNA candidate genes (Methods) involved in amino acid and fatty acid metabolism. *GRIK3* encodes a glutamate receptor that plays a role in synaptic transmission and has been linked to mental health including neuroticism^37^.

A SNP-based MT-nDNA interaction analysis was then performed. First, nuclear loci were identified that showed a different magnitude of association with each of the three neuroticism traits depending on MT-haplogroup by comparing each SNP effect from H-haplogroup-stratified GWAS with any other-haplogroup-stratified GWAS (Methods). After clumping (*r*^*2*^ > 0.4 within 250 kb of lead SNP), five independent nuclear loci (two for the general factor of neuroticism and three for anxiety/tension) showed a significantly different effect between haplogroups at *P* < 1 × 10^−5^ (Supplementary Data 16). Following a formal interaction analysis (Methods) in the entire population conducted on the five loci attaining statistical significance, two retained genome-wide statistical significance at *P* < 5 × 10^−8^ (Supplementary Data 17). Neither of these markers was found to co-segregate with mitochondrial haplogroup (Supplementary Methods).

rs72771986 was associated with the general factor of neuroticism in the X haplogroup-stratified nGWAS (*β* = −0.198, *se* = 0.033, *P* = 2.28 × 10^−9^, *MAF* = 0.11, *N* = 3,851). rs181210427, was associated with anxiety/tension in T-haplogroup-stratified nGWAS (*β* = −0.152, *se* = 0.026, *P* = 3.31 × 10^−9^, *MAF* = 0.02, *N* = 26,893). Neither of these SNPs was associated with their respective neuroticism trait in either the H haplogroup-stratified GWASs, or when all haplogroups were combined. Furthermore, there was a significantly greater effect of rs72771986 for general neuroticism, and rs181210427 for anxiety/tension compared to the H haplogroup effect (rs72771986 ${P}_{{diff}}$ = 6.60 × 10^−9^, rs181210427 ${P}_{{diff}}=$9.11 × 10^−8^) and for all haplogroups combined (rs72771986 ${P}_{{diff}}$ = 1.58 × 10^−9^, rs181210427 ${P}_{{diff}}$ = 1.97 × 10^−9^). Finally, the formal interaction model, for the general neuroticism factor, rs72771986 showed significant evidence of interaction with X haplogroup (${\beta }_{{interaction}}$ = 0.196, *se* = 0.033, *P* = 3.30 × 10^−9^), and rs181210427 demonstrated a comparable effect for its interaction with the T-haplogroup (${\beta }_{{interaction}}$ = 0.154, *se* = 0.027, *P* = 9.04 × 10^−9^) for anxiety/tension (Supplementary Data 17).

**Fig. 4:**
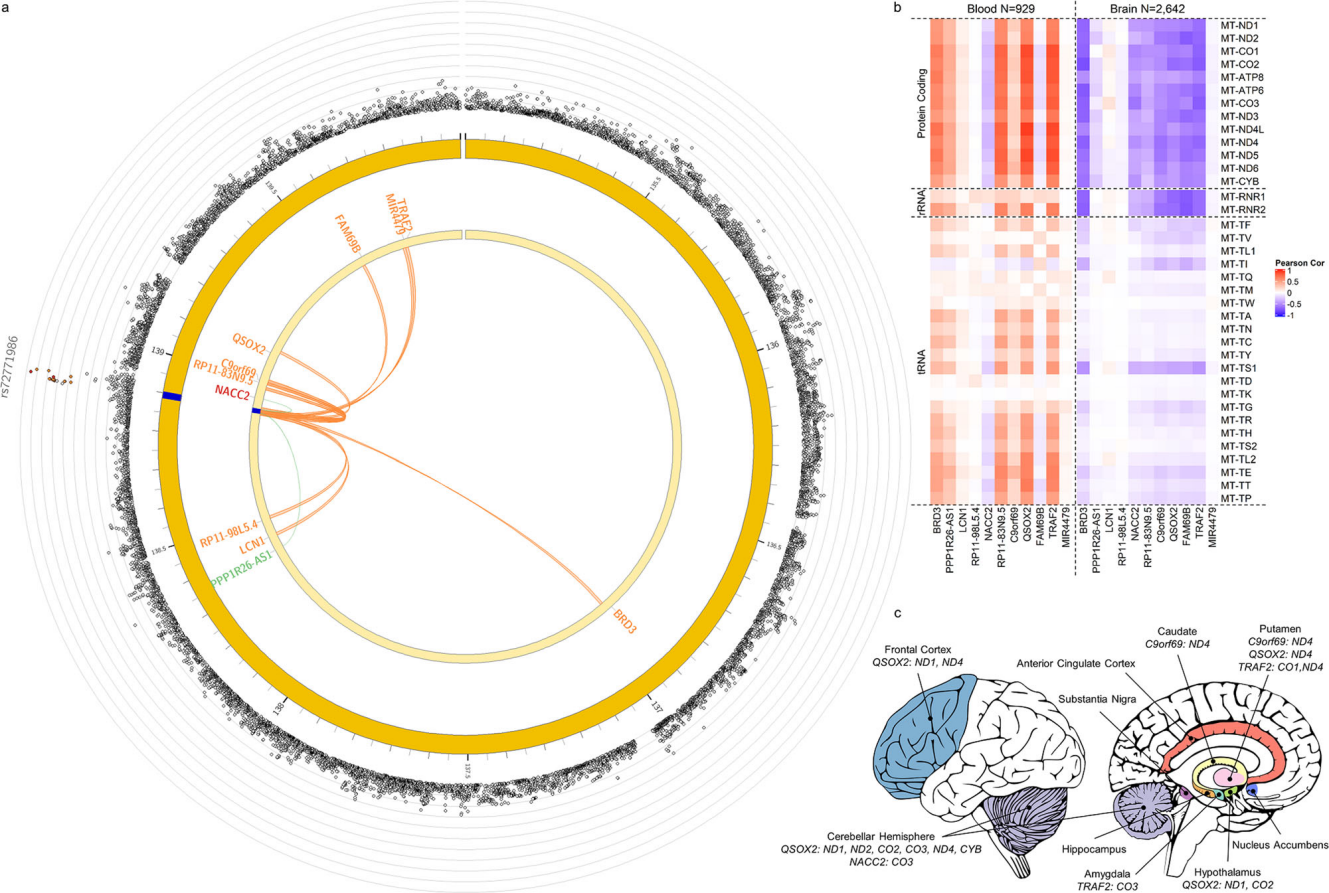
Genes mapped to the general neuroticism locus identified by rs72771986 using FUMA and their co-expression pattern with MT genes in blood and brain tissues in GTEx. **a** shows 11 genes mapped to general neuroticism locus due to chromatin interactions (orange), or eQTLs (green), or both (red). The outer layer shows –log10 *P* values (two-sided t test) of stratified nGWAS results for X haplogroup. SNPs in LD with labelled lead SNP with *r*^2^ > 0.8, >0.6, >0.4 > 0.2 and ≤0.2 are coloured in red, orange, green, blue and grey respectively. The middle and inner layers are chromosome rings with and without coordinate, whereas the blue bar indicates the trait-associated genomic locus. **b** shows the correlation of gene expression between mapped genes and MT genes in blood (*N* = 929) and brain (*N* = 2642) tissues in GTEx. **c** shows the pairs of nDNA-MT genes showing negatively correlated gene expression (*r* < −0.8) in different brain regions (each region is coloured differently), suggesting negative co-expression. Figure plotted using cerebroViz^106^ package in R.

rs72771986 is an intergenic marker and using FUMA^28^ it was annotated to a 17.4 kb region (138,886,227—138,903,635 bp) on chromosome 9 alongside another 14 candidate markers and 11 mapped genes (Supplementary Data 18, 19). Evidence from RNA-seq^38–49^ and Hi-C^46,50–52^ indicate that this locus presents *cis*-eQTLs for two genes and has chromatin interactions with ten genes nearby, including a MT-nDNA candidate gene quiescin sulfhydryl oxidase 2, *QSOX2*, (*q* = 2.18 × 10^−40^ and *q* = 4.56 × 10^−26^ in fetal and adult cortex respectively) (Fig. 4a and Supplementary Data 20, 21). To explore the potential biological cause of observed MT-nDNA interaction, we investigated protein-protein interactions using STRING^53^ and gene co-expression in GTEx v8^38^. We found that the expression of genes from this locus are positively correlated with the expression of most MT genes in blood (*P* < 0.05/56,200 where 56,200 is the number of genes in GTEx) (Fig. 4b). For *MT-ATP8, MT-CO1, MT-CO2, MT-ND4, MT-ND4L*, and *MT-ND5*, the correlation of gene expression between them and *QSOX2* and TNF receptor associated factor 2 (*TRAF2*) from this locus reached 0.8, suggesting that these genes may be under the same co-expression network (Supplementary Fig. 5). In other tissues, such as brain, the expression of genes mapped to this locus is negatively correlated with most MT genes (*P* < 0.05/56,200), although not reaching co-expression threshold of 0.8 (Fig. 4b and all main tissues in Supplementary Figs. 5–31). We examined different brain regions (Supplementary Figs. 32–41) and found that the expression pattern between genes from this locus and MT genes were similar. There is evidence of negative co-expression between *QSOX2* and several MT protein-coding genes in the cerebellar hemisphere, frontal cortex, putamen and hypothalamus (Fig. 4c). Transmembrane protein 250 (*TMEM250*, that used to be known as *C9orf69*), NACC family member 2 (*NACC2*), and *TRAF2* were also negatively correlated with MT gene expression in different brain regions (Fig. 4c). Regarding protein-protein interaction, no evidence was observed between any of these 11 mapped genes and 13 MT protein-coding genes in the STRING database (Supplementary Fig. 42).

We mapped 33 genes to the rs181210427 locus using FUMA (Supplementary Fig. 43). Although none of these genes was an MT-nDNA candidate gene, 1 (astrotactin 2; *ASTN2*) showed the same co-expression pattern as the general neuroticism locus with MT genes in blood and brain. *ASTN2* reached co-expression threshold of 0.8 with *MT-ATP8*, *MT-CO1*, *MT-CO2*, *MT-CO3*, *MT-ND4L*, and *MT-ND5* in a positive direction in blood but did not reach the threshold in brain tissue (Supplementary Figs. 5, 6 and 32, 41). Furthermore, no evidence of a protein-protein interaction was found using STRING (Supplementary Fig. 42).

## Discussion

General neuroticism, anxiety/tension, and worry/vulnerability are genetically and phenotypically associated with physical and mental health outcomes, including longevity. GWAS have shown little overlap in autosomal variation associated with each of these three phenotypes, indicating they each possess a different genetic aetiology which, subsequently, confers different level of risk for disease outcomes. These large scale genetic analyses have omitted variation in the mitochondrial genome as a source of trait variance. The current study addressed this gap in our understanding of the genetic architecture of neuroticism and illustrated the importance of the bi-factor model, and of examining nuclear DNA associations with complex trait variation within mitochondrial haplogroup.

First, we found that, as with nuclear DNA, associations between mtDNA differed for each of the three neuroticism phenotypes and that these different genetic associations may provide a partial explanation as to the risk and protective effects that these neuroticism traits have on health. Neuroticism is a risk factor for Parkinson’s disease^10^. Haplogroup T was associated with general neuroticism and has previously been linked to risk of Parkinson’s disease^25^.

Furthermore, the haplogroups we identified as being associated with general neuroticism (K) and with worry/vulnerability (U and the **UK** super haplogroup) have, along with two super **UK** haplogroup-defining markers m.12372 G > A and m.11467 A > G in *MT-ND4*^54^, been linked to Alzheimer’s disease,^54,55^ which is associated with higher neuroticism.^9^ *MT-ND4* shows lower level of transcription in the temporal cortex of Alzheimer’s disease patients compared with controls^56^. In the current study, two mtDNA SNPs in the *MT-ND4* gene---m.11299 T > C (haplogroup K defining marker) and m.11467 A > G (super **UK** defining marker), although not the lead mtDNA SNPs---were significantly associated with general neuroticism and with worry/vulnerability, respectively, and so may provide a partial explanation for the link between neuroticism and Alzheimer’s disease.

Second, the direction of effect for markers in *MT-ND4* is reversed for the general factor of neuroticism compared to worry/vulnerability. Previous work examining the links between the three neuroticism traits and health outcomes has consistently identified that whilst greater levels of general neuroticism are associated with poorer health outcomes and a reduction in longevity, higher worry/vulnerability and anxiety/tension^27^ levels are associated with better health and longer life. This relationship has been identified at the phenotypic^17,18^ and genetic^19^ level between the three factors of neuroticism with physical health. Our finding that genetic variation in *MT-ND4* is negatively associated with the general factor of neuroticism, but positively associated with worry/vulnerability, may indicate a specific gene in which variation is linked to differential susceptibility to Alzheimer’s disease in addition to its role in neuroticism.

Third, to provide a comprehensive understanding of how mitochondria may influence neuroticism, we examined MT-nDNA genes. These results also indicate differences in the genetic architecture of these three factors of neuroticism. Whilst 48 genes are associated with at least one of the three neuroticism traits, there was little overlap: 44 of the 48 associated MT-nDNA genes are associated with only a single neuroticism trait. Gene set analysis found no evidence that the MT-nDNA gene set, nor any of the subunits assessed, were enriched for any of the three neuroticism traits. This indicates that the MT-nDNA gene associations are unlikely to be related to their role in mitochondria function specifically. Instead, they may be associated with neuroticism due to pleiotropy with the MT-nDNA genes being a part of other biological systems or through their association with other traits. Our finding that the MT-nDNA genes associated with the general factor of neuroticism and anxiety/tension contained an overrepresentation of genes associated with sleep patterns and hypertension respectively would support this.

Fourth, using genome-wide, gene-based, and single variant analysis, we found nuclear DNA associations that are dependent on mitochondrial haplogroup. H-haplogroup-specific genome-wide associations were identified for worry/vulnerability by performing autosomal GWAS on the three factors of neuroticism stratified by mitochondrial haplogroup. Genetic correlations of less than 1 indicated genome-wide differences in the underlying genetic architecture of worry/vulnerability between haplogroups. An examination of the genetic correlations by haplogroup indicated a possible protective effect of the H-haplogroup where, as the contributions to the genetic correlation made by the H-haplogroup increased, the magnitude of the genetic correlations with bipolar disorder decreased from positive in a group composed of all non-H-haplogroup carriers, to zero when examining only individuals with the H-haplogroup, and negative following adjustment to control for the shared genetic factors between H carriers and non-H carriers.

Gene-based analysis stratified by haplogroup identified 14 genes associated with the factors of neuroticism (Supplementary Data 15). Three gene-level associations showed a greater level of association with their respective neuroticism phenotypes than the unstratified GWAS (General factor of neuroticism H-haplogroup, *GRIK3, P* = 2.36 × 10^−6^, anxiety/tension T-haplogroup, *DEFB132*, *P* = 6.50 × 10^−7^, worry/vulnerability K-haplogroup, *PCCB*, *P* = 1.46 × 10^−6^). Furthermore, one association, *ETFA* in the V-haplogroup, was only associated with general neuroticism following stratification by haplogroup. Three of the genes identified by stratifying the GWASs based on haplogroup have evidence as to their biological relevance, both to mitochondria and brain function. Both *PCCB* and *EFTA* are related to mitochondrial activity. Where *PCCB* encodes a protein that is a subunit of the mitochondrial enzyme PCC, *EFTA* plays a role in the first stages of beta-oxidation of the mitochondrial fatty acids. *GRIK3* is linked to the glutamate receptors (the primary excitatory neurotransmitter in the mammalian brain). Specifically, the product of *GRIK3* belongs to the kainite family of glutamate receptors.

Two loci interacted with haplogroup X and T, for general neuroticism and anxiety/tension, respectively. Whilst both loci are on chromosome 9 and show clear evidence of functionality, being eQTLs and showing chromatin interactions with 11 (general factor of neuroticism) and 33 (anxiety/tension) genes, respectively, there was little overlap in the identity of the genes implicated through functional annotation across these two loci, again highlighting heterogeneity between these two factors of neuroticism. Importantly, the nuclear genes identified through functional annotation of the general factor of neuroticism and anxiety/tension interaction loci were found to form brain tissue-specific co-expression networks with protein-coding mitochondrial genes, with negative correlations observed for the brain, and positive correlations for the blood. When examining the general factor of neuroticism, some evidence was found linking negative co-expression between rRNA and tRNA mitochondrial genes with the nDNA genes, the magnitude of these correlations is greater for the protein-coding mitochondrial genes.

Our study highlights the importance of a careful interpretation of MT-GWAS discoveries. In the current study, simulation using genotype and haplogroup allowed us to reduce the number of independent signals, identify the single associated unit (either haplogroup or single variant) most likely responsible for driving the associations with the neuroticism traits, and provided information on the cohort-specific correlation structure in addition to knowledge of phylogenetics. As an example, the homoplasmic (not linked to any haplogroup) marker m.73 A > G is associated with worry/vulnerability but, in our data, this marker is commonly expressed in super haplogroup **HV**, as well as haplogroup B, F, P, and R. However, the majority of our samples carrying m.73 A > G belong to super haplogroup **HV**, and so there is a high correlation (*r*^2^ = 0.84) between m.73 A > G and super haplogroup **HV**. By taking into consideration this relationship specific to our data set, we were able to avoid allocating the association of m.73 A > G with worry/vulnerability.

The mtDNA associations with worry/vulnerability and with general neuroticism indicate that mtSNPs and haplogroups have pleiotropic effects where they also associate with blood traits such as RDW, lymphocyte count, and WBC in UK Biobank. Consistent with previous studies, the special factors of neuroticism had a different direction of phenotypic and genetic correlation with blood traits, such as with RDW and WBC, compared to those derived using general neuroticism. Higher levels of RBW and WBC have been linked to a greater severity of depression symptoms^57^. Depression is strongly phenotypically and genetically associated with general neuroticism, indicating the mtDNA variation may provide a, partial, explanation for the link between depression and blood cell traits.

A limitation of this study is that it was not possible to examine the degree to which the relationships between neuroticism and psychiatric traits^58,59^, or specific/rare diseases^60,61^ that are underrepresented in UK Biobank, was due to pleiotropic mitochondrial DNA. This limitation arose due to the current practice of GWAS omitting the mitochondrial genome from large meta-analytic consortia. The current paper illustrates that the mitochondrial genome is associated with neuroticism, an important risk factor for psychiatric traits^62,63^, at the level of single variant, haplogroup, and through interactions with autosomal regions. The importance of including the mitochondrial genome in future GWAS meta-analyses of psychiatric and health traits is underscored by our finding that the effect sizes for associated mitochondrial variants is of a similar magnitude as autosomal SNPs for the same traits (m.497 C > T, general neuroticism, *β* = −0.03, *se* = 0.008, *P* = 3.82 × 10^−5^, rs113434679, General factor of neuroticism, *β* = 0.02, *se* = 0.002, *P* = 2.55 × 10^−21^ ^19^).

In our MT-nDNA co-segregation GWAS, we found multiple nDNA loci co-segregating with different MT haplogroups. Considering that the associations found in MT haplogroup and marker association studies are weak at the magnitude of *P*s = 10^−4^ to 10^−5^, we explored whether our haplogroup and MT-GWAS association findings were confounded by these co-segregating loci. We found no evidence that these co-segregating nDNA loci were strongly associated with our neuroticism traits and thus ruled out the possibility of confounding. Guarding against co-segregation of nuclear and mitochondrial variants can be implemented as a quality control step while conducting future MT studies on other traits and diseases. Whether the association between complex traits or diseases and MT-nDNA co-segregation loci has resulted in false discoveries in previous MT studies needs further exploration. In the current study, only common variants were examined for their association with the three neuroticism traits. Future work should also examine the contributions made by rare and heterplasmic variants in the mitochondrial genome. Additionally, the inclusion of samples composed of a broader array of haplogroups---or sequence data---would allow additional sources of genetic variation to be examined for association with neuroticism.

Large scale GWAS have begun to identify reliably the molecular genetic aetiology of neuroticism phenotypes: traits that are phenotypically and genetically associated with health, financial, and psychiatric differences^17,19^. However, critics of GWAS note that the effect sizes identified for individual variants are low and the heritability captured by array data consists of a subset of the total genetic effect, and that this limits the degree to which GWAS results may benefit human health and wellbeing^64^. Critics also contend that, GWAS does not necessarily identify causal variants due to factors such as linkage disequilibrium^64^. However, GWAS have contributed insights into disease biology that have been supported by clinical translation,^65^ have begun to show their potential in risk stratification at the population level,^66^ and in identifying targets for pharmacological intervention^67^. Furthermore GWAS data can be used to examine potentially causal relationships between traits, for example, by Mendelian randomisation^68^. These contributions have been made using GWAS conducted on autosomes and in some instances the X chromosome^69^ with mitochondrial DNA being absent from such interrogation. In the current study we perform that initial interrogation to show the general factor of neuroticism and the special factors of anxiety/tension and worry/vulnerability are genetically distinct with different single marker, haplogroup, and nDNA by MT-DNA interactions contributing to trait variation and their links to health outcomes.

## Methods

### Ethical compliance

This UK Biobank project (10279) was approved by the National Research Ethics Service Committee North West-Haydock (REC reference: 11/NW/0382). An electronic signed consent was obtained from all participants.

### Samples

Individuals in this study were drawn from the UK Biobank^70^. UK Biobank is composed of 502,655 individuals recruited between 2006 and 2010 and who are 39-73 years of age (mean = 56.9, SD = 8.0). These participants were asked to provide detailed information regarding their background, health and lifestyle, as well as being asked to undergo cognitive and physical testing with blood, urine, and saliva samples being stored upon consent. Participants were genotyped for more than 800,000 genome-wide genetic markers using stored blood samples and either the UK BiLEVE Axiom™ or the UK Biobank Axiom™ array. A comprehensive description of the full range of phenotype measurements, the array design, blood sample collection, DNA extraction and the initial data quality control have been published^70,71^.

### Phenotype derivation and quality control (QC)

A general factor, and two special factors, of neuroticism were studied here. They were derived in the same manner as Gale et al.^17^ and described as follows. Neuroticism was assessed in UK Biobank using a 12 item scale from the Short-scale Eysenck Personality Questionnaire-Revised^16^. UK Biobank participants were presented with each of the 12 items (e.g., Do you ever feel ‘just miserable’ for no reason?) on a touchscreen. Participants were instructed to “Work quickly and do not think about the exact meaning of the question.” and were given the option to respond in one of four ways: “Yes” (coded ‘1’), “No” (coded ‘2’), “Do not know” (coded ‘−1’), and “Prefer not to answer” (coded ‘−3’). A total of 100,981 did not answer “Yes” or “No” to all 12 questions, and so were excluded from further analyses, leaving 401,674 participants.

To investigate the associations between factors of personality with health and other outcomes it is often best to use a bi-factor model^72^. A bi-factor model distributes variance from a set of items into a smaller number of independent factors. Specifically, the variance for items is separated into that which is related to a general factor and that which is related to one or more special factors that are independent of the general factor.

The general and special factors were derived by applying exploratory structural equation modelling analysis to the 12 items and applying an oblique bi-factor Geomin rotation^73,74^. A Geomin rotation of a *p* × *k* factor loading matrix *Λ*, where *p* is the number of items (rows), *k* refers to the number of factors (columns), and *ε* is a constant equal to 0.01^75^, is one in which correlated axes are rotated to minimise the criterion:

1 $${{{{{\rm{geomin}}}}}}({\wedge })={\mathop{\sum }\limits_{i=1}^{p}\left(\mathop{\prod }\limits_{r=1}^{k}({\lambda }_{ir}^{2}+\varepsilon )\right)}^{(1/k)}$$

The criterion is therefore at its minimum when every item loads on a single factor. In the case of a bi-factor Geomin rotation, this criterion is only applied to the last *k*−1 factors (the special factors), so the criterion is minimised when each item loads on only one of these factors.

**Fig. 5:**
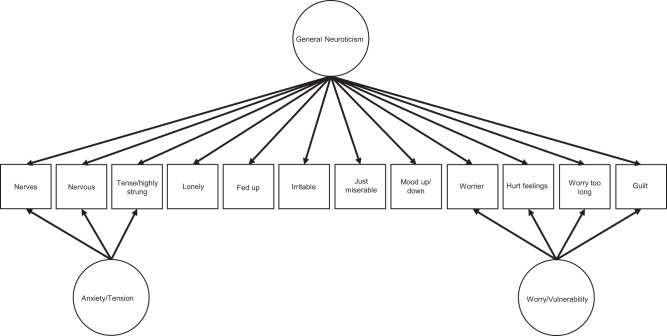
Bi-factor model explaining the relationship between neuroticism factors and 12 personality measurements. Schematic showing the exploratory bi-factor analysis of the 12 items in the Short-scale Eysenck Personality Questionnaire-Revised^16^ with factors shown using circles and items shown in squares. As illustrated, a general factor, labelled general neuroticism, can be extracted from the variance shared by each 12 items. Two special factors, anxiety/tension and worry/vulnerability, could also be extracted from the variance that remained following the extraction of the general factor of neuroticism. These special factors do not share variance with the general factor, and so allows for the identification of genetic variants that are distinct from the general factor. The figure illustrates only the items that each of the special factors draw most heavily from, with the path coefficients presented in full in Supplementary Data 22.

Three measures of neuroticism were derived using this method: a general factor of neuroticism that is composed of variance drawn from across each of the 12 items and two special factors of neuroticism that are composed of the variance that remained once the general factor was removed. These special factors are aspects of neuroticism (anxiety/tension and worry/vulnerability), that whilst captured by The Short-scale Eysenck Personality Questionnaire-Revised^16^ are uncorrelated with the general factor. The anxiety/tension special factor primarily explains variance from the items of “Would you call yourself a nervous person?” (*λ* = 0.60), “Do you suffer from ‘nerves’?” (*λ* = 0.49), and “Would you call yourself tense or ‘highly strung’?” (*λ* = 0.35). Worry/vulnerability explains variance from the items of “Do you worry too long after an embarrassing experience?” (*λ* = 0.57), “Are your feelings easily hurt?” (*λ* = 0.40), “Are you a worrier?” (*λ* = 0.31), and “Are you often troubled by feelings of guilt?” (*λ* = 0.31) (see Fig. 5 and Supplementary Data 22). The correlations between the general factor and the two special factors were defined as zero in the model whereas the correlation between the two special factors was *r* = 0.311 (*P* < 0.0001). Each participant’s score on each of the three factors was then estimated using the regression scoring method. The correlation between the estimates of the latent score of the general factor of neuroticism with anxiety/tension and worry/vulnerability were *r* = 0.07, and *r* = 0.12 (*P* < 2.220 × 10^−16^) respectively. The correlation between the estimated scores on the two special factors was *r* = 0.427 (*P* < 2.220 × 10^−16^). Small differences between the correlations derived using the factors within the model and those derived using the participants’ estimates are expected^76^.

Following the extraction of the three factors of neuroticism, the effects of sex, age, genotyping array, batch, assessment centres, and population stratification (estimated using the first 40 autosomal genetic principal components) were removed from each phenotype at cohort level using linear regression in R v3.6.1^77^. The residuals derived from the linear regression of each of the three neuroticism phenotypes were used as the phenotype in subsequent analyses.

### Mitochondrial DNA (mtDNA) processing and analysis

An overview of the sample size at QC and used for association analysis can be found in the flowchart in Supplementary Fig. 1.

### MT genotype data QC

This study was restricted to 332,047 unrelated White British UK Biobank samples that were previously used in an autosomal GWAS project on neuroticism^78^. Detailed sample-quality control information is available elsewhere^78^. In brief, samples who withdrew consent, harbour extreme heterozygosity or missingness on autosomes, have sex chromosome aneuploidies, have mismatched self-reported and genetically inferred sex, or have more than ten putative third degree relatives were removed. Samples with non-British ancestry based on self-report and a principal components analysis and related individuals based on genomic relationship cut-off of 0.025 were also removed.

Based on the genotyping array, the UK Biobank dataset was separated into two groups, named UKBL (36,292 samples) for UK BiLEVE Axiom™ and UKBB (295,755 samples) for UK Biobank Axiom™. Quality control of the mtDNA genotypes was conducted in PLINKv1.90b6.20^79^ and was performed for each subset separately as two genotyping arrays share less than 90% mtDNA content (180 markers on UK BiLEVE Axiom™ and 243 markers on UK Biobank Axiom™ with an overlap of 158). Markers that failed QC in any of the batches (95 batches for UKBB and 11 batches for UKBL), monomorphic markers, extremely rare markers (MAF < 0.0001) and markers with a call rate <0.98 were removed. Samples with missingness > 0.05 were also removed. After QC, 295,120 samples and 205 markers and 36,237 samples and 151 markers were retained in UKBB subset and UKBL subset, respectively. No putative heteroplasmies (i.e., variants called as heterozygous) were removed, leaving only homoplasmic calls for mtDNA markers^71^.

### MT imputation and imputed data QC

Imputation and quality control were performed separately by genotyping array (UKBB and UKBL). Following quality control, genotype data were first transformed from PLINK format into OXFORD format using SHAPEIT2^80^. Second, imputation of mtDNA variants was performed using IMPUTE2^81^ with a window size of 16.6 kb (i.e., full length of mtDNA) using 2,504 samples from MT-1000G Phase 3^82^ as a reference panel. Third, hard calls were made from genotype probabilities in PLINKv1.90b6.20^79^ using default certainty threshold of 0.9. Very few heterozygous calls (less than 0.001%) were produced by imputation due to putative heteroplasmies and these markers were set to missing. Fourth, quality control was applied to the imputed data. Markers with missing rate >0.05 (including those set to missing due to putative heteroplasmies), MAF < 0.01 (including monomorphic markers), MAF difference >0.02 between UKBB and UKBL subsets, MAF difference >0.1 between UKBB + UKBL and 1000 G GBR (91 British samples), call rate <0.95 and imputation info <0.3 were removed. Samples with missingness >0.05 were also removed.

Following QC, 295,120 samples and 162 mtDNA markers (106 genotyped + 56 imputed) passed QC in the UKBB subset. In UKBL, 36,237 samples and 134 mtDNA markers (92 genotyped + 42 imputed) passed final QC. There were 121 markers shared between UKBB and UKBL and 175 different markers in total.

### MT haplogroup and super haplogroup derivation

Post-QC MT genotype data was transformed from PLINK format to hsd format using R v3.6.1^77^. Afterwards, MT haplogroup was derived using haplogrep2^83,84^ based on Phylotree build 17^85^. Haplogroup was set to missing for individuals with a quality score lower than 0.9, resulting in final sample size of 324,488.

To increase group size for statistical power, haplogroups were clustered into haplogroups A-Z and HV according to the first/two uppercase English letter (e.g., J1ab1 = J, H5a = H). Using Phylotree^85^, and similar to previous work^86^, V is a subgroup of HV0a, H is a subgroup of HV, K is a subgroup of U8b, J and T is subgroup of JT. We further clustered phylogenetically linked haplogroups into ‘super’ haplogroups (e.g, H, HV, and V into **HV**, J and T into **JT**, finally U and K were clustered into **UK)**. Super haplogroup is presented in bold to distinguish it from haplogroups.

### MT-GWAS and MT-haplogroup association analysis

MT-GWAS was performed separately in UKBL and UKBB and subsequently meta-analysed using the default fixed-effect inverse-variance weighted method in PLINK v1.90b6.20^79^. Based on prior work using permutation, an alpha level of 0.001 within each of the three neuroticism traits was used to determine the statistical significance of any associations identified^86^. A total of 175 MT-markers were examined in 269,506 samples with phenotype information (UKBB: 239,816 + UKBL: 29,690). Haplogroup association analysis was performed in R v3.6.1^77^ at cohort level (i.e., UKBB and UKBL combined) using the phenotypic residuals and the haplogroups derived from previous steps in 263,883 samples with neuroticism phenotype and having a haplogroup quality score over 0.9. Ten common haplogroups with frequency over 0.01 were examined (H, HV, V, U, K, J, T, I, W, and X) using two models.

The first model examines the effect that deviation from the most common haplogroup, haplogroup H, has on the three neuroticism phenotypes. Haplogroups with frequencies lower than 0.01 (A-G, L-N, P-R, and Y-Z) were combined into one group termed ‘Other’, resulting in 11 categories. Next, linear regression was performed on the phenotypic residuals fitting the haplogroups as a categorical variable using the most common haplogroup, haplogroup H, as the reference. The significance threshold was set to 0.05/10 as there were 10 comparisons in total.

The second model examined the effect of belonging to a specific haplogroup when compared to the rest of the population (e.g., H v Non H). Each haplogroup was tested sequentially against the sum of the remaining, where the tested haplogroup was re-coded as 1, and the non-tested haplogroups were pooled into one group and re-coded as 0. The phenotypic residuals were then regressed on the re-coded haplogroups by fitting non-tested haplogroups as the reference. The significance threshold was set to 0.05/10 as there were 10 common haplogroups tested.

Super haplogroup association analysis was conducted as above, using **HV** as the reference group in the first model. We considered a super haplogroup, rather than its contributing haplogroups, associated with the phenotype if the *P* value of super haplogroup analysis was lower than the threshold of 0.005 and lower than any of its contributing haplogroups.

In instances where the associated haplogroup and the associated mtDNA SNP are incompletely correlated with each other (i.e., not haplogroup-defining markers) simulations were performed to investigate the number of independent associations and the associated unit (mtDNA SNP or haplogroup) that most likely drove the signal (Supplementary Methods). To ensure that any associated MT-GWAS variant or any haplogroup associations with the three factors of neuroticism were not capturing nuclear DNA variation, co-segregation analyses were conducted (Supplementary Methods).

### Variance explained by haplogroup

Variance in each of the three neuroticism factors explained by MT-haplogroups was estimated using ANOVA by comparing the full model (i.e., with the ten common haplogroups fitted as covariates) and the reduced model (i.e., without fitting haplogroups). This is likely to be an underestimation of the total effect of the mitochondrial genome as subgroups and homoplasmic SNPs can provide additional genetic information and coverage of the mitochondrial genome, even with imputation is sparse, however, it does provide a lower level estimate. To provide a comparison with the heritability captured by autosomal SNPs, per-chromosome heritability was estimated in SLDSC^87,88^ using 1000 G European reference panel. A partitioned heritability model with 23 binary annotations was created to capture the total heritability and to estimate the proportion of total heritability captured by each chromosome. The per-chromosome heritability was calculated as the proportion of total heritability explained by each chromosome times the total heritability estimated in a non-partitioned model implemented in Hill et al. (2020)^19^.

### PheWAS scan

A PheWAS scan was performed using the data generated by Yonova-Doing et al.^26^ to examine the pleiotropic effects of neuroticism-associated mtDNA with 896 traits in UK Biobank. These traits including 594 non-cancer illness from hospital records in ICD10, 166 self-reported illness, 11 binary traits, and 125 quantitative traits, classified into nine categories, which are anthropometric, cardiovascular, endocrine/diabetes, fitness/longevity, gastrointestinal, haematology/dermatology/immune, infection, musculoskeletal/trauma, neurology/eye/psychiatry, renal/urology, reproductive, respiratory/ear-nose-throat, and others.

For the lead mtDNA SNPs that were associated with neuroticism, a look-up was performed where, for mtDNA haplogroup associations, look-ups were performed for all haplogroup-defining markers on the phylogenetic tree^89^ that were available in Yonova-Doing, et al.^26^ Haplogroup-trait associations were defined as the strongest SNP-trait association for that haplogroup. Multiple testing was conducted across number of markers and traits and the significant threshold was set to FDR < 0.05.

Where mtDNA pleiotropic associations were found for the neuroticism traits, phenotypic and genetic correlation were estimated. The phenotypic correlation was estimated in R using Pearson correlation, whereas the genetic correlation was estimated in LDSC^90^ using pre-calculated LD scores in 1000 G EUR samples.

### Nuclear DNA (nDNA) processing and analysis

#### nDNA imputed data QC

Imputation of nuclear DNA markers was performed by UK Biobank^71^. We extracted the imputed data for our initial 332,047 unrelated white British samples and performed chromosome-wise quality control in PLINK2^79^ by removing markers with INFO < 0.3, MAF < 0.0005, call rate <0.95, Hardy-Weinberg equilibrium *P* value < 10^−50^ and removing samples with genotyping missingness >0.1. Multi-allelic markers in the remaining data were also removed. A total of 19,651,021 markers and 332,042 samples passed QC.

#### MT-haplogroup-stratified nDNA GWAS and MT-nDNA interaction analysis

To examine if the effect of autosomal variation on the three neuroticism phenotypes differed by mitochondrial haplogroup, an interaction analysis was conducted. To reduce the statistical burden, we first ran haplogroup-stratified GWAS of nuclear DNA markers within each of the ten common MT-haplogroups (H, HV, V, J, T, U, K, I, W, and X). Haplogroup-stratified GWASs (i.e., restricting the samples according to their haplogroups before performing an autosomal GWAS) were conducted on the three neuroticism traits using phenotypic residuals and 8,912,253 post-QC nDNA imputed markers with MAF > 0.01 in 270,055 samples with neuroticism phenotype in PLINK2^79^. This is to ensure the expected minimum frequency of interaction to be over 0.0001 (nDNA MAF 0.01 × MT MAF 0.01). We then tested whether the effect of the same nuclear allele varies between haplogroups as an indication of MT-nDNA interaction by comparing the beta estimates of the same nuclear marker between two stratified GWASs. We used haplogroup H as a reference for all comparisons. Z-statistics for testing the difference in SNP beta estimates was R v3.6.1^77^ as follows,

2 $$Z=\frac{{b}_{1}-{b}_{2}}{\sqrt{{{se}}_{1}^{2}+{{se}}_{2}^{2}}}$$

where ${b}_{1}$ and ${b}_{2}$ are the effect sizes of the same SNP in two GWASs and ${{se}}_{1}$ and ${{se}}_{2}$ are the corresponding standard errors of the estimates. We considered markers to be statistically significant if the interaction *P* value was lower than 1 × 10^−5^, and the association *P* value was lower than 5 × 10^−8^ in any one of the two GWASs under comparison.

For those nuclear loci showing evidence of differing association with the neuroticism traits between haplogroups, formal MT-nDNA interaction model (where the effects of nDNA marker, MT-haplogroup and nDNA-by-MT interaction were evaluated jointly in the model) was conducted using PLINK2^79^ in the whole UK Biobank data set (Supplementary Methods). To ensure that any interactions identified were not better explained as a main effect of nuclear DNA captured by haplogroup, co-segregation analysis was performed (Supplementary Methods).

### Follow-up analysis

#### MT-nDNA candidate genes

MT-nDNA candidate genes were downloaded from IMPI 2020Q3^91^ and Mito Carta 3.0^92^. Both datasets were mapped to GRCh37 using ENSEMBL (version 102)^93^ to link the two databases and take account of the difference in gene IDs and symbols due to different genome build. In instances where no match was obtained, mapping of IMPI data was based on its reported ENSEMBL gene ID or gene symbol, whereas mapping of MitoCarta data was based on its reported gene symbol or WikiGene ID. This resulted in 2,220 MT-nDNA candidate genes, 1085 of which were reported in both data base, 1,084 were reported in IMPI exclusively and the remaining 51 were reported in Mito Carta exclusively (Supplementary Data 23).

#### Gene-based and gene-set analysis

Three data sources were examined using MAGMA (version v1.09)^94^ to perform gene-based/gene-set analysis. These were the GWAS summary statistics of the three neuroticism traits from Hill et al.^19^ of the haplogroup-stratified nGWAS, and of the co-segregation GWAS.

Regarding gene-based analysis for MT-nDNA candidate genes, 2,099 autosomal MT-nDNA candidate genes were extracted and tested using the full GWAS^19^ where traditional GWAS method was applied without stratifying on haplogroups. MT-nDNA candidate genes associated with any neuroticism traits at *P* < 0.05/2,099 = 2.38 × 10^−5^ were reported. As for haplogroup-stratified nGWAS and co-segregation GWAS, 18,337 autosomal protein-coding genes available in the GWAS summary data were examined, resulting in a significance threshold of 0.05/18,337 = 2.73 × 10^−6^. Note, while comparing gene-based results from haplogroup-stratified nGWAS with that from the full GWAS, we restricted the input SNPs to those shared between two GWASs, keeping the same number of SNPs for each gene to maintain the same statistical power. Afterwards, we looked at the gene-based associations from the full GWAS for significant associations identified in the haplogroup-stratified for comparison.

For gene-set analysis, we first tested whether there was enrichment of neuroticism-associated genes in the whole set of 2,099 MT-nDNA candidates. The IMPI dataset was further categorised into three non-overlapping categories: genes known to encode mitochondrial proteins, protein-coding genes predicted to physically interact with mitochondria (IMPI mitochondrial localisation score ≥ 0.7), and protein-coding genes associated with or having an ancillary effect on mitochondria^91^. The MitoCarta gene set was divided into 14 overlapping categories according to mouse tissue-specific mitochondrial protein localisation (cerebrum, cerebellum, brainstem, spinal cord, liver, kidney, heart, skeletal muscle, adipose, stomach, small intestine, large intestine, testis, and placenta)^92^. For neuroticism traits, we also tested 149 gene-sets of mitochondrial pathways from MitoCarta. In gene-set analysis, all protein-coding genes were fitted as background genes, in addition we examined the enrichment of the MitoCarta gene set following the removal of all potential mt-nDNA genes in the control gene set in order to guard against confounding by including nuclear mitochondrial genes in the control set. As translocations of mtDNA to the nuclear genome are relatively common^82^, we additionally blasted^95^ the entire MT-genome to the nuclear genome and extracted all nDNA genes which overlapped with those MT-nDNA aligned regions. This gene-set was tested for MT-nDNA co-segregation. We applied multiple testing correction within trait and reported gene-sets with an FDR < 0.05.

#### Genome-wide haplogroup specific neuroticism associations

To examine if the nuclear DNA polygenic signal associated with the neuroticism factors varied by MT haplogroup, genetic correlations were derived using LDSC (version v1.01)^90^ using pre-calculated LD scores in 1000 G EUR samples. Genetic correlations were derived between each GWAS stratified by haplogroup (for example, a GWAS on the general factor of neuroticism only in those individuals who belonged to the H haplogroup) with the meta-analysed GWASs conducted on each of the other haplogroups (For example, a meta-analysis of the GWASs on the general factor of neuroticism conducted in the nine remaining haplogroups). Evidence of a different nuclear polygenic signal by MT haplogroup was indicated by a genetic correlation that was significantly lower than 1. In these instances mtCOJO analysis^96^, performed in GCTA (version v1.92.1)^97^, was used to remove overlapping genetic factors, leaving the nuclear polygenic signal associated with the neuroticism phenotypes that is specific to any one mitochondrial haplogroup. mtCOJO was run using pre-calculated LD scores in 1000 G EUR samples, assuming the shared genetic components were mainly due to horizontal pleiotropy (Supplementary Method). In addition, genetic correlations were performed on the haplogroup-stratified GWAS examining traits previously found to be associated with worry/vulnerability^19^ (Supplementary Data 24).

#### Functional annotation

For MT-haplogroup-stratified nDNA GWAS, determination of genomic risk locus, gene-mapping and annotation were performed in FUMA (version v1.3.6a)^28^ using the SNP2GENE function. Briefly, we uploaded summary statistics and pre-defined lead SNPs (haplogroup-X-stratified nGWAS and rs72771986 for general neuroticism, and haplogroup-T-stratified nGWAS and rs181210427 for anxiety/tension) into the FUMA web interface. FUMA searched for other SNPs in 1000 G Phase3 EUR^82^ database within a 250 kb window of and in LD (*r*^*2*^ > 0.6) with our lead SNPs. Genes were mapped to the genomic risk locus by three methods: positional mapping (genes within 10 kb window of the locus based on ANNOVAR^98^); cis-eQTL mapping (genes within 1 mb window of the locus of which the expression level is mediated by any of the candidate SNPs at FDR < 0.05 in any tissue or cell type in the following database^38–49^); and 3D chromatin interaction mapping (genes with promoters located in regions that interact with the genomic risk locus at FDR < 1 × 10^−6^ in any tissue or cell type in the following databases^46,50–52^).

In addition to ANNOVAR, eQTL, and chromatin status, SNPs were annotated for combined annotation dependent depletion (CADD) scores^99^, regulome database (RDB) scores^100^, and genes were annotated for the probability of being loss-of-function intolerant (pLI)^101^ and non-coding residual variation intolerance scores (ncRVIS)^102^.

For MT-nDNA candidate genes associated with our neuroticism traits, we performed functional annotation enrichment analysis in FUMA^28^ using the GENE2FUNC function, by comparing our gene lists to all functional-annotated gene-sets in the following database^103–105^. All 2,220 MT-nDNA candidate genes were set as background genes to remove enrichment due to MT-related functions and pathways. Gene sets with an FDR < 0.05 were reported.

Protein-protein interactions between 44 mapped nDNA genes and 13 MT protein-coding genes were examined in STRING^53^.

Gene co-expression analysis was performed in R v3.6.1^77^ using GTEx v8 TPM data^38^. Within each tissue with sample size over 50, we calculated the Pearson correlation of gene TPM between each pair of 44 mapped nDNA genes and 37 MT-genes. The significance threshold was set to 0.05/56,200 as there are 56,200 genes in total. We additionally applied the same analysis for all brain sub-regions.

#### Co-segregation GWAS

To explore if co-segregation between MT-haplogroup and nuclear DNA was driving the association between haplogroup identity and the three neuroticism phenotypes, we performed nDNA GWAS of MT haplogroups. We did not find evidence that our MT associations were driven by nuclear DNA. Details of co-segregation GWAS are in Supplementary Note, Supplementary Figs. 44–56, and Supplementary Data 25–27.

#### Power calculation

Power of co-segregation GWAS and MT-nDNA interaction GWAS was derived and validated using simulation. Details of power calculation are in Supplementary Method, Supplementary Figs. 57–63, and Supplementary Table 1.

### Reporting summary

Further information on research design is available in the Nature Portfolio Reporting Summary linked to this article.

## Supplementary information

Supplementary Information

Description of Additional Supplementary Files

Supplementary Data 1

Supplementary Data 2

Supplementary Data 3

Supplementary Data 4

Supplementary Data 5

Supplementary Data 6

Supplementary Data 7

Supplementary Data 8

Supplementary Data 9

Supplementary Data 10

Supplementary Data 11

Supplementary Data 12

Supplementary Data 13

Supplementary Data 14

Supplementary Data 15

Supplementary Data 16

Supplementary Data 17

Supplementary Data 18

Supplementary Data 19

Supplementary Data 20

Supplementary Data 21

Supplementary Data 22

Supplementary Data 23

Supplementary Data 24

Supplementary Data 25

Supplementary Data 26

Supplementary Data 27

Reporting Summary

## Data Availability

UK Biobank data used in this study are available via the UK Biobank data access process (see http://www.ukbiobank.ac.uk/register-apply/). The Summary statistics for MT associations are available in Supplementary Data 4-6, Summary statistics for the autosomal associations are available through GWAS catalog at https://www.ebi.ac.uk/gwas/ under accession codes GCST90264121, GCST90264122, GCST90264123, GCST90264124, GCST90264125, GCST90264126, GCST90264127, GCST90264128, GCST90264129, GCST90264130, GCST90264131, GCST90264132, GCST90264133, GCST90264134, GCST90264135, GCST90264136, GCST90264137, GCST90264138, GCST90264139, GCST90264140, GCST90264141, GCST90264142, GCST90264143, GCST90264144, GCST90264145, GCST90264146, GCST90264147, GCST90264148, GCST90264149, and GCST90264150.

## Code availability

Codes see Supplementary Method.
