# Supplementary material for: The contributions of mitochondrial and nuclear mitochondrial genetic variation to neuroticism
Source: Nat Commun. 2023 May 30;14:3146. doi: 10.1038/s41467-023-38480-y (PMC10229642; doi:10.1038/s41467-023-38480-y)
Supplement: Supplementary file 1 — Supplementary Information [file 41467_2023_38480_MOESM1_ESM.pdf]

Supporting Information

The contributions of mitochondrial and nuclear mitochondrial genetic variation to neuroticism

Charley Xia[1,2], Sarah J. Pickett[3], David C. M. Liewald[1,2], Alexander Weiss[2], Gavin Hudson[4], W. David Hill[1,2*].

1 Lothian Birth Cohort studies, University of Edinburgh, 7 George Square, Edinburgh EH8 9JZ, UK

2 School of Philosophy, Psychology and Language Sciences, Department of Psychology, University of Edinburgh, 7 George Square, Edinburgh, EH8 9JZ, UK

3 Wellcome Centre for Mitochondrial Research and Translational and Clinical Research Institute, The Medical School, Newcastle University, Newcastle upon Tyne NE2 4HH, UK.

4 Wellcome Centre for Mitochondrial Research and Biosciences Institute, Newcastle University, Newcastle upon Tyne NE2 4HH, UK.

*Correspondence should be addressed to Dr W. David Hill (David.Hill@ed.ac.uk)

Table of Contents

Table of Contents 2

Supplementary Figures 7

Supplementary Figure 1. Flowchart of MT/nuclear DNA processing, and sample size at each QC and analysis. 7

Supplementary Figure 2. Showing the phenotypic variance explained by chromosome length for the General Factor of Neuroticism, Anxiety/Tension, and Worry/Vulnerability. 8

Supplementary Figure 3. Reference allele frequency of mitochondrial markers across haplogroups, their correlation with selected haplogroups and the pairwise marker LD between them in UK Biobank. 9

Supplementary Figure 4. Results of 1000 simulations. 10

Supplementary Figure 5. Pearson correlations of gene expression between 37 MT genes and 11 genes mapped from the general neuroticism locus and 33 mapped genes from anxiety/tension locus on chromosome 9 in 929 GTEx v8 blood tissue samples. 12

Supplementary Figure 6. Pearson correlations of gene expression between 37 MT genes and 11 genes mapped from the general neuroticism locus and 33 mapped genes from anxiety/tension locus on chromosome 9 in 2,642 GTEx v8 brain tissue samples. 14

Supplementary Figure 7. Pearson correlations of gene expression between 37 MT genes and 11 genes mapped from the general neuroticism locus and 33 mapped genes from anxiety/tension locus on chromosome 9 in 258 GTEx v8 adrenal gland tissue samples. 16

Supplementary Figure 8. Pearson correlations of gene expression between 37 MT genes and 11 genes mapped from the general neuroticism locus and 33 mapped genes from anxiety/tension locus on chromosome 9 1,204 GTEx v8 adipose tissue samples. 18

Supplementary Figure 9. Pearson correlations of gene expression between 37 MT genes and 11 genes mapped from the general neuroticism locus and 33 mapped genes from anxiety/tension locus on chromosome 9 1,335 GTEx v8 blood vessel tissue samples. 19

Supplementary Figure 10. Pearson correlations of gene expression between 37 MT genes and 11 genes mapped from the general neuroticism locus and 33 mapped genes from anxiety/tension locus on chromosome 9 in 459 GTEx v8 breast tissue samples. 20

Supplementary Figure 11. Pearson correlations of gene expression between 37 MT genes and 11 genes mapped from the general neuroticism locus and 33 mapped genes from anxiety/tension locus on chromosome 9 in 779 GTEx v8 colon tissue samples. 22

Supplementary Figure 12. Pearson correlations of gene expression between 37 MT genes and 11 genes mapped from the general neuroticism locus and 33 mapped genes from anxiety/tension locus on chromosome 9 in 1,445 GTEx v8 esophagus tissue samples. 24

Supplementary Figure 13. Pearson correlations of gene expression between 37 MT genes and 11 genes mapped from the general neuroticism locus and 33 mapped genes from anxiety/tension locus on chromosome 9 in 861 GTEx v8 heart tissue samples. 26

Supplementary Figure 14. Pearson correlations of gene expression between 37 MT genes and 11 genes mapped from the general neuroticism locus and 33 mapped genes from anxiety/tension locus on chromosome 9 in 89 GTEx v8 kidney tissue samples. 28

Supplementary Figure 15. Pearson correlations of gene expression between 37 MT genes and 11 genes mapped from the general neuroticism locus and 33 mapped genes from anxiety/tension locus on chromosome 9 in 226 GTEx v8 liver tissue samples. 30

Supplementary Figure 16. Pearson correlations of gene expression between 37 MT genes and 11 genes mapped from the general neuroticism locus and 33 mapped genes from anxiety/tension locus on chromosome 9 in 578 GTEx v8 lung tissue samples. 31

Supplementary Figure 17. Pearson correlations of gene expression between 37 MT genes and 11 genes mapped from the general neuroticism locus and 33 mapped genes from anxiety/tension locus on chromosome 9 in 803 GTEx v8 muscle tissue samples. 32

Supplementary Figure 18. Pearson correlations of gene expression between 37 MT genes and 11 genes mapped from the general neuroticism locus and 33 mapped genes from anxiety/tension locus on chromosome 9 in 619 GTEx v8 nerve tissue samples. 33

Supplementary Figure 19. Pearson correlations of gene expression between 37 MT genes and 11 genes mapped from the general neuroticism locus and 33 mapped genes from anxiety/tension locus on chromosome 9 in 180 GTEx v8 ovary tissue samples. 34

Supplementary Figure 20. Pearson correlations of gene expression between 37 MT genes and 11 genes mapped from the general neuroticism locus and 33 mapped genes from anxiety/tension locus on chromosome 9 in 328 GTEx v8 pancreas tissue samples. 35

Supplementary Figure 21. Pearson correlations of gene expression between 37 MT genes and 11 genes mapped from the general neuroticism locus and 33 mapped genes from anxiety/tension locus on chromosome 9 in 283 GTEx v8 pituitary tissue samples. 36

Supplementary Figure 22. Pearson correlations of gene expression between 37 MT genes and 11 genes mapped from the general neuroticism locus and 33 mapped genes from anxiety/tension locus on chromosome 9 in 245 GTEx v8 prostate tissue samples. 37

Supplementary Figure 23. Pearson correlations of gene expression between 37 MT genes and 11 genes mapped from the general neuroticism locus and 33 mapped genes from anxiety/tension locus on chromosome 9 in 162 GTEx v8 salivary gland tissue samples. 39

Supplementary Figure 24. Pearson correlations of gene expression between 37 MT genes and 11 genes mapped from the general neuroticism locus and 33 mapped genes from anxiety/tension locus on chromosome 9 in 1,809 GTEx v8 skin tissue samples. 41

Supplementary Figure 25. Pearson correlations of gene expression between 37 MT genes and 11 genes mapped from the general neuroticism locus and 33 mapped genes from anxiety/tension locus on chromosome 9 in 187 GTEx v8 small intestine tissue samples. 42

Supplementary Figure 26. Pearson correlations of gene expression between 37 MT genes and 11 genes mapped from the general neuroticism locus and 33 mapped genes from anxiety/tension locus on chromosome 9 in 241 GTEx v8 spleen tissue samples. 43

Supplementary Figure 27. Pearson correlations of gene expression between 37 MT genes and 11 genes mapped from the general neuroticism locus and 33 mapped genes from anxiety/tension locus on chromosome 9 in 359 GTEx v8 stomach tissue samples. 45

Supplementary Figure 28. Pearson correlations of gene expression between 37 MT genes and 11 genes mapped from the general neuroticism locus and 33 mapped genes from anxiety/tension locus on chromosome 9 in 361 GTEx v8 testis tissue samples. 47

Supplementary Figure 29. Pearson correlations of gene expression between 37 MT genes and 11 genes mapped from the general neuroticism locus and 33 mapped genes from anxiety/tension locus on chromosome 9 in 653 GTEx v8 thyroid tissue samples. 48

Supplementary Figure 30. Pearson correlations of gene expression between 37 MT genes and 11 genes mapped from the general neuroticism locus and 33 mapped genes from anxiety/tension locus on chromosome 9 in 142 GTEx v8 uterus tissue samples. 49

Supplementary Figure 31. Pearson correlations of gene expression between 37 MT genes and 11 genes mapped from the general neuroticism locus and 33 mapped genes from anxiety/tension locus on chromosome 9 in 156 GTEx v8 vagina tissue samples. 50

Supplementary Figure 32. Pearson correlations of gene expression between 37 MT genes and 11 genes mapped from the general neuroticism locus and 33 mapped genes from anxiety/tension locus on chromosome 9 in 152 GTEx v8 brain - amygdala tissue samples. 52

Supplementary Figure 33. Pearson correlations of gene expression between 37 MT genes and 11 genes mapped from the general neuroticism locus and 33 mapped genes from anxiety/tension locus on chromosome 9 in 176 GTEx v8 brain – anterior cingulate cortex tissue samples. 54

Supplementary Figure 34. Pearson correlations of gene expression between 37 MT genes and 11 genes mapped from the general neuroticism locus and 33 mapped genes from anxiety/tension locus on chromosome 9 in 246 GTEx v8 brain - caudate tissue samples. 55

Supplementary Figure 35. Pearson correlations of gene expression between 37 MT genes and 11 genes mapped from the general neuroticism locus and 33 mapped genes from anxiety/tension locus on chromosome 9 in 215 GTEx v8 cerebellar hemisphere tissue samples. 57

Supplementary Figure 36. Pearson correlations of gene expression between 37 MT genes and 11 genes mapped from the general neuroticism locus and 33 mapped genes from anxiety/tension locus on chromosome 9 in 209 GTEx v8 brain – frontal cortex tissue samples. 58

Supplementary Figure 37. Pearson correlations of gene expression between 37 MT genes and 11 genes mapped from the general neuroticism locus and 33 mapped genes

from anxiety/tension locus on chromosome 9 in 197 GTEx v8 brain - hippocampus tissue samples. 59

Supplementary Figure 38. Pearson correlations of gene expression between 37 MT genes and 11 genes mapped from the general neuroticism locus and 33 mapped genes from anxiety/tension locus on chromosome 9 in 202 GTEx v8 brain - hypothalamus tissue samples. 60

Supplementary Figure 39. Pearson correlations of gene expression between 37 MT genes and 11 genes mapped from the general neuroticism locus and 33 mapped genes from anxiety/tension locus on chromosome 9 in 246 GTEx v8 brain – nucleus accumbens tissue samples. 61

Supplementary Figure 40. Pearson correlations of gene expression between 37 MT genes and 11 genes mapped from the general neuroticism locus and 33 mapped genes from anxiety/tension locus on chromosome 9 in 205 GTEx v8 brain - putamen tissue samples. 62

Supplementary Figure 41. Pearson correlations of gene expression between 37 MT genes and 11 genes mapped from the general neuroticism locus and 33 mapped genes from anxiety/tension locus on chromosome 9 in 139 GTEx v8 substantia nigra tissue samples. 63

Supplementary Figure 42. STRING multiple protein search results. 65

Supplementary Figure 43. Genes mapped to the anxiety/tension locus on chromosome 9 by FUMA. 66

Supplementary Figure 44. Manhattan plot for H-haplogroup co-segregation GWAS. 67

Supplementary Figure 45. Manhattan plot for HV-haplogroup co-segregation GWAS. 68

Supplementary Figure 46. Manhattan plot for V-haplogroup co-segregation GWAS. 69

Supplementary Figure 47. Manhattan plot for J-haplogroup co-segregation GWAS. 70

Supplementary Figure 48. Manhattan plot for T-haplogroup co-segregation GWAS. 71

Supplementary Figure 49. Manhattan plot for U-haplogroup co-segregation GWAS. 72

Supplementary Figure 50. Manhattan plot for K-haplogroup co-segregation GWAS. 73

Supplementary Figure 51. Manhattan plot for I-haplogroup co-segregation GWAS. 74

Supplementary Figure 52. Manhattan plot for W-haplogroup co-segregation GWAS. 75

Supplementary Figure 53. Manhattan plot for X-haplogroup co-segregation GWAS. 76

Supplementary Figure 54. Manhattan plot for Super-**HV**-haplogroup co-segregation GWAS. 77

Supplementary Figure 55. Manhattan plot for Super-**JT**-haplogroup co-segregation GWAS. 78

Supplementary Figure 56. Manhattan plot for Super-**UK**-haplogroup co-segregation GWAS. 79

Supplementary Notes 80

Co-segregation GWAS 80

Supplementary Method 81

MT Genotype Data QC 81

MT Genotype “Pre-phasing” and Imputation 83
MT Imputation QC 85
MT GWAS 86
MT Haplogroup Derivation 87
MT Haplogroup Association Analysis 89
Simulations 90
Nuclear DNA Imputation QC 91
Nuclear DNA GWAS (nGWAS) 93
MAGMA 93
LD Score regression (LDSC) 94
Multi-Trait-based Conditional and Joint (mtCOJO) 95
Power calculation for co-segregation GWAS 95
Supplementary Table 1. 2 x 3 contingency table under co-segregation GWAS assumptions. 96
Supplementary Figure 57. Comparing rxy. Expected and observed values converged at line $y = x$ (red dotted). 98
Supplementary Figure 58. Comparing t. Expected and observed values converged at line $y = x$ (red dotted). 98
Supplementary Figures 59. Power calculation for co-segregation GWAS. 100
Power calculation for interaction GWAS 104
Supplementary Figure 60. Comparing β. Expected and observed values converged at line $y = x$ (red dotted) 105
Supplementary Figure 61. Comparing $se(\beta)$. Expected and observed values converged at line $y = x$ (red dotted). 106
Supplementary Figure 62. Comparing Z statistics of testing the difference in β between two groups. Expected and observed values converged at line $y = x$ (red dotted) 106
Supplementary Figures 63. Power calculation for interaction GWAS. 108
Supplementary References 113

Supplementary Figures

Supplementary Figure 1. Flowchart of MT/nuclear DNA processing, and sample size at each QC and analysis.

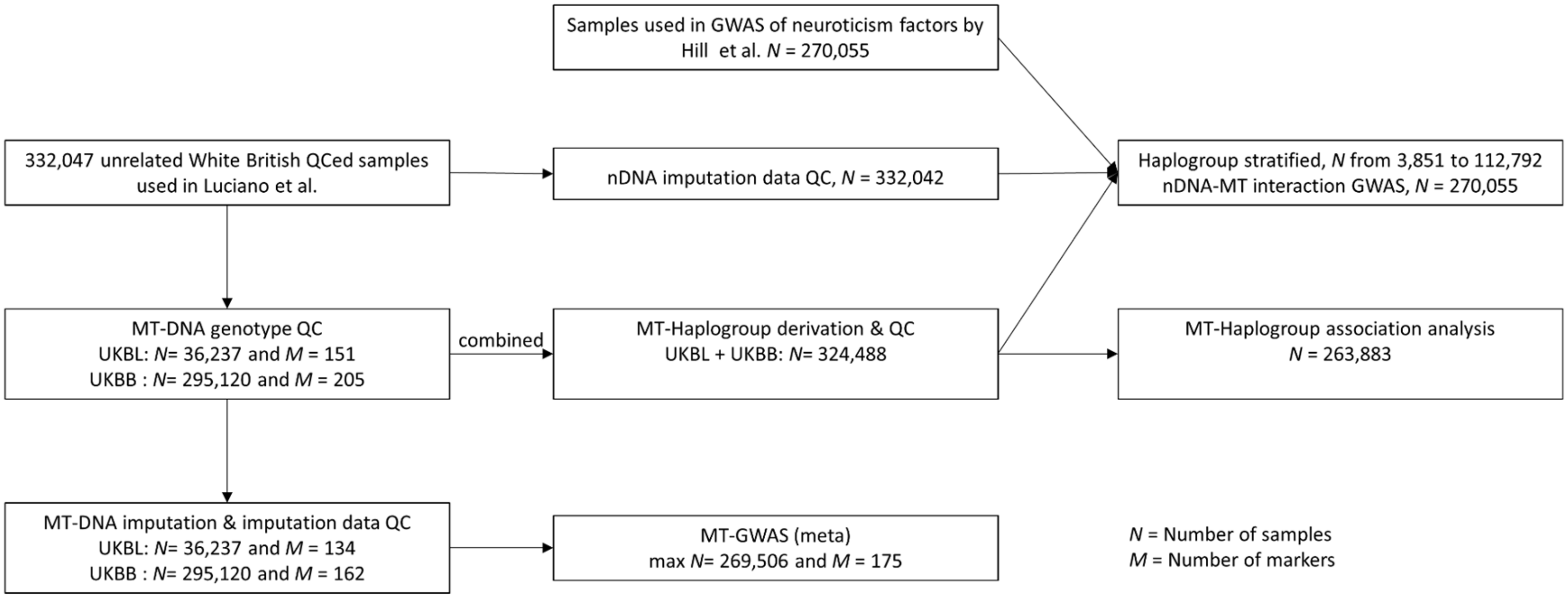

Supplementary Figure 2. Showing the phenotypic variance explained by chromosome length for the General Factor of Neuroticism, Anxiety/Tension, and Worry/Vulnerability.

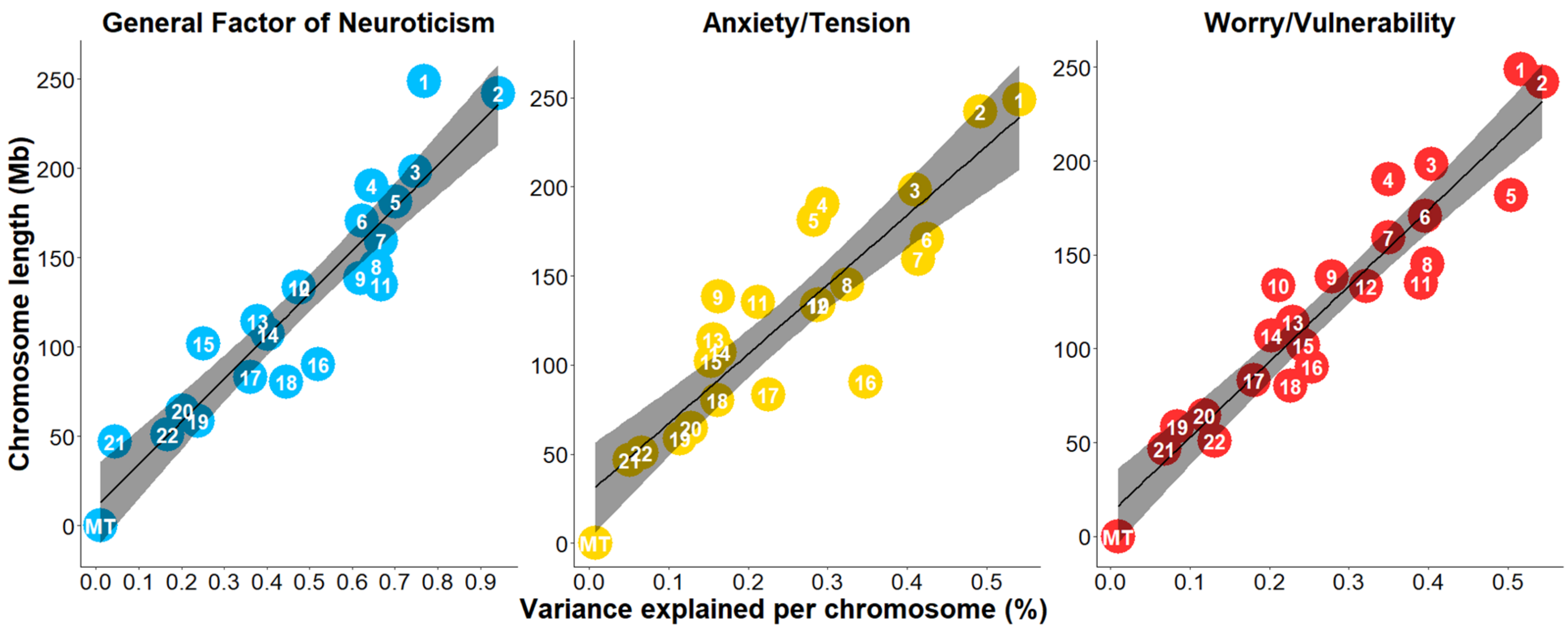

This figure shows that the variance explained by common autosomal SNPs and the variance explained by the mitochondrial haplogroup for each of the three factors of neuroticism is proportional to the length of the chromosome. Y-axis: chromosome length (Mb). Y-axis: variance explained (%). Black line: fitted linear regression line. Grey area (error bands): 95% confidence intervals of the regression slope.

Supplementary Figure 3. Reference allele frequency of mitochondrial markers across haplogroups, their correlation with selected haplogroups and the pairwise marker LD between them in UK Biobank.

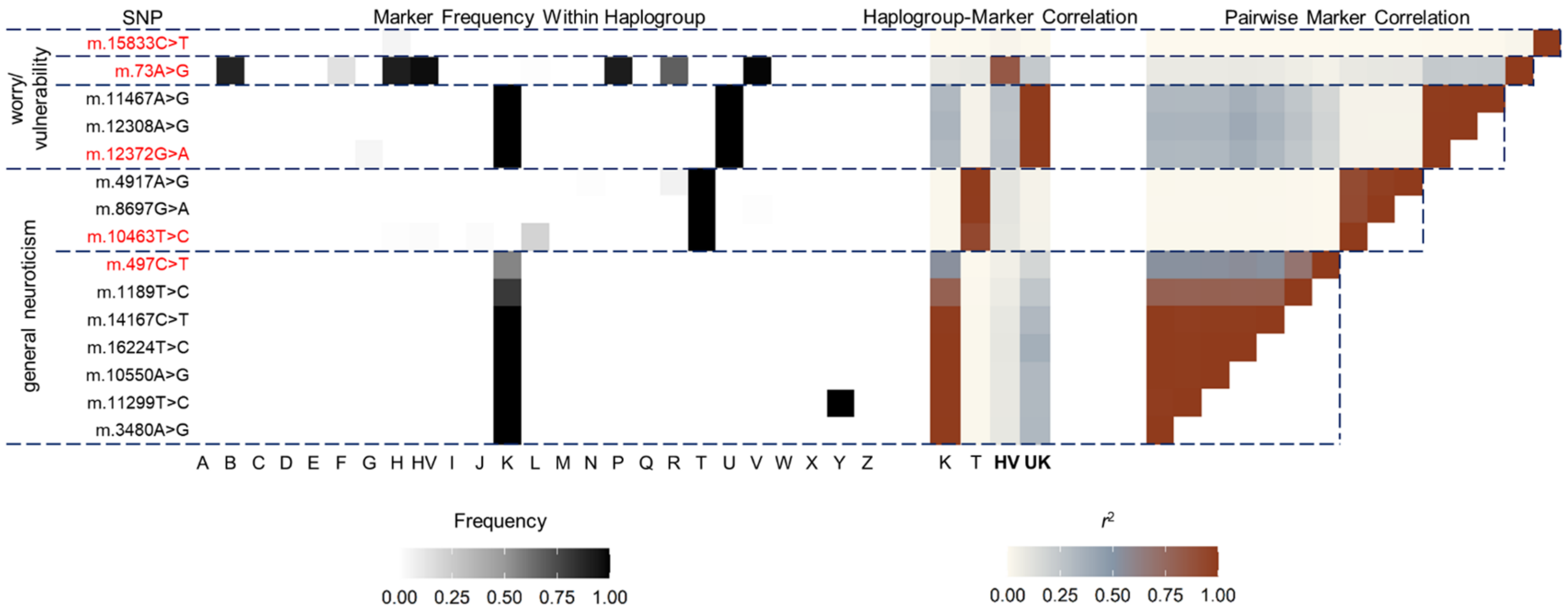

In MT-GWAS, 15 mitochondrial markers (10 for general neuroticism and five for worry/vulnerability) were found to be associated at α = 0.001. Single marker allele frequencies within-haplogroup are shown on the bottom left with the correlations between markers with haplogroup K, T, super HV (**HV**), and super UK (**UK**) being shown in the centre. Population level (i.e. across all haplogroups found in UK Biobank) pair-wise marker correlations are shown on the bottom right. Correlation between markers was measured in r^2. The single marker with the lowest P-value describing its association with one of the neuroticism traits is shown in red. The figure shows collinearity for the associated 15 markers and suggests that some of the markers were contributing to the same underlying association signal. For example, for the 10 markers associated with general neuroticism, seven of them were highly correlated with each other and with haplogroup K, whereas three were correlated with each other and haplogroup U. This pattern of correlations between groups of single markers and haplogroups indicates that there are two independent signals associated with the general factor of neuroticism, one from haplogroup K and one from haplogroup U.

Supplementary Figure 4. Results of 1000 simulations.

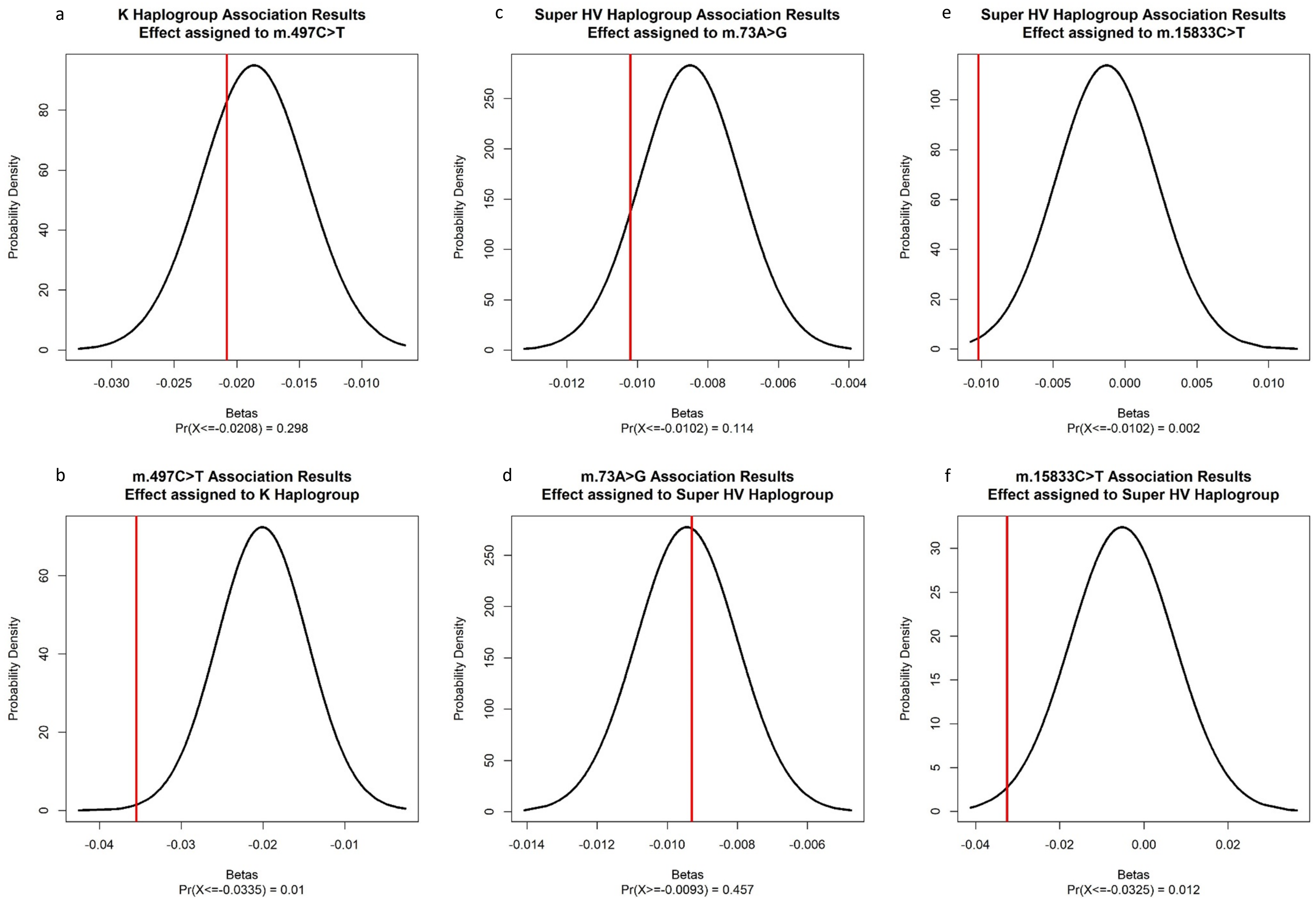

In each scenario we assigned an effect to a genetic marker (either a SNP or a haplogroup). The assigned effect was the same as the observed effect for that marker in the real data. We then simulate an uncorrelated environmental variable to account for all unmeasured effects. The simulated phenotype was computed as the sum of the genetic and environmental effects, which has mean of 0 and an SD of 1. We performed linear regression for the alternative genetic marker, that is, when the effect was assigned to a SNP, we performed association for the haplogroup and vice versa. An empirical distribution of the betas was made by 1000 replicas. An empirical p value was calculate by comparing the observed beta in the real data with the empirical distribution (one-sided). **Panel a** and **b** show that, the genetic marker driven the observed signal for general neuroticism is likely due to m.497C>T because when the causal effect was assigned to m.497C>T, it can replicate the effect observed for K haplogroup, but not the other way around. **Panel c** and **d** show that, the observed associations of worry/vulnerability with m.73A>G and super HV haplogroup are likely driven by the same signal. **Panel e** and **f** show that, both m.15833C>T and super HV haplogroup contribute independent signals to the observed worry/vulnerability associations.

Supplementary Figure 5. Pearson correlations of gene expression between 37 MT genes and 11 genes mapped from the general neuroticism locus and 33 mapped genes from anxiety/tension locus on chromosome 9 in 929 GTEx v8 blood tissue samples.

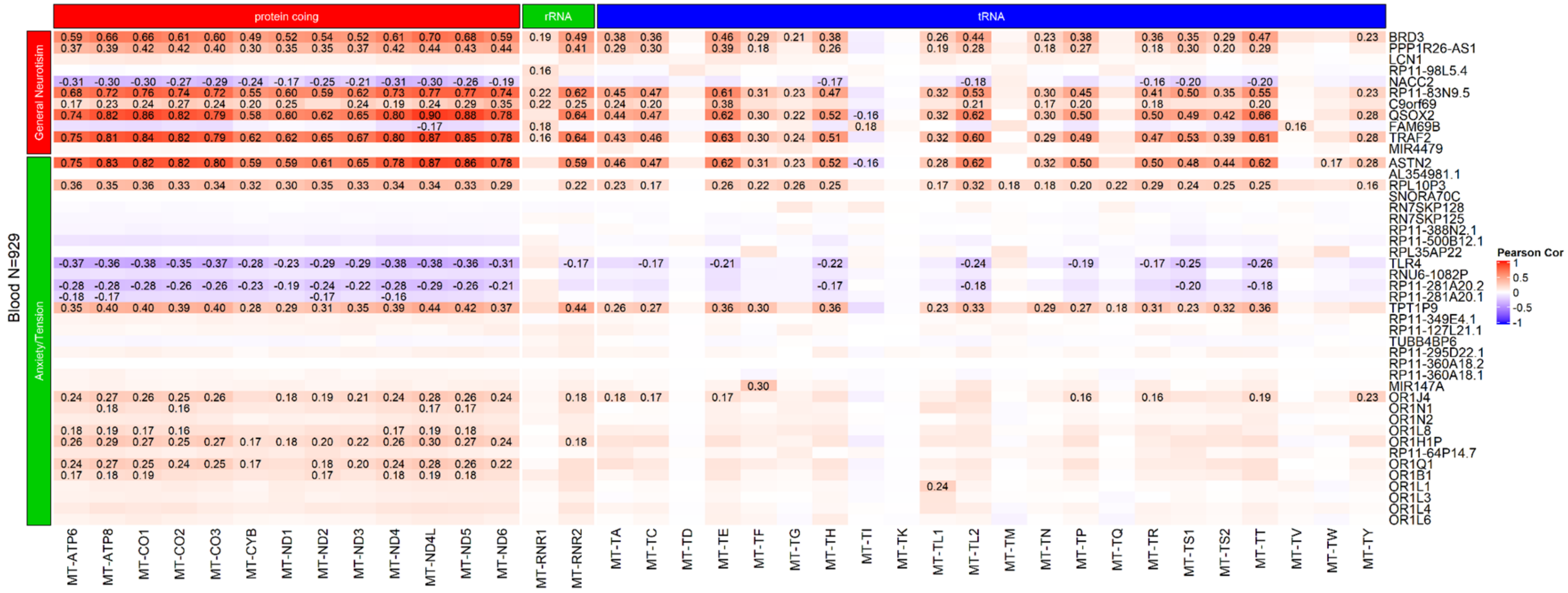

Colour in cells indicates the direction of a Pearson correlation and when labelled, indicate a statistically significant correlation at P < 0.05/56,200 (two-sided) where 56,200 is the total number of genes in GTEx v8. Rows are split by traits where general neuroticism is in red and anxiety/tension is in green. Columns are split by gene type, where protein-coding is in red, rRNA is in green, and tRNA is in blue.

Supplementary Figure 6. Pearson correlations of gene expression between 37 MT genes and 11 genes mapped from the general neuroticism locus and 33 mapped genes from anxiety/tension locus on chromosome 9 in 2,642 GTEx v8 brain tissue samples.

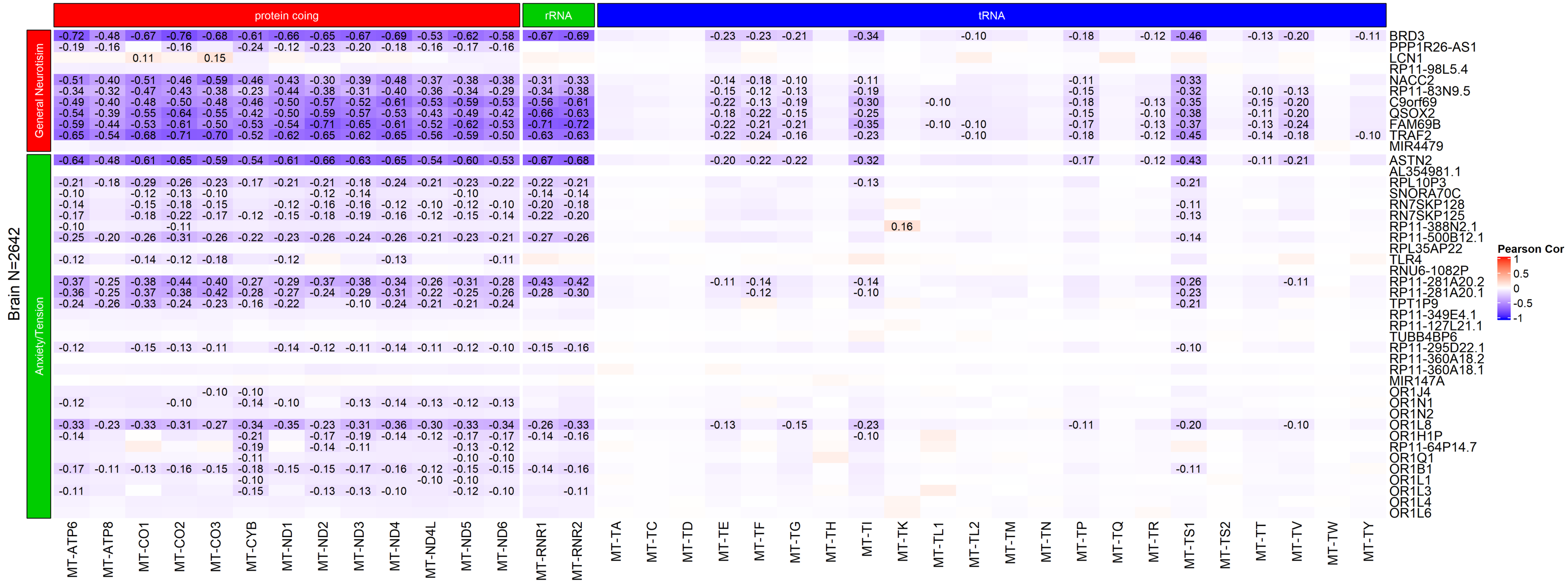

Colour in cells indicates the direction of a Pearson correlation and when labelled, indicate a statistically significant correlation at P < 0.05/56,200 (two-sided) where 56,200 is the total number of genes in GTEx v8. Rows are split by traits where general neuroticism is in red and anxiety/tension is in green. Columns are split by gene type, where protein-coding is in red, rRNA is in green, and tRNA is in blue.

Supplementary Figure 7. Pearson correlations of gene expression between 37 MT genes and 11 genes mapped from the general neuroticism locus and 33 mapped genes from anxiety/tension locus on chromosome 9 in 258 GTEx v8 adrenal gland tissue samples.

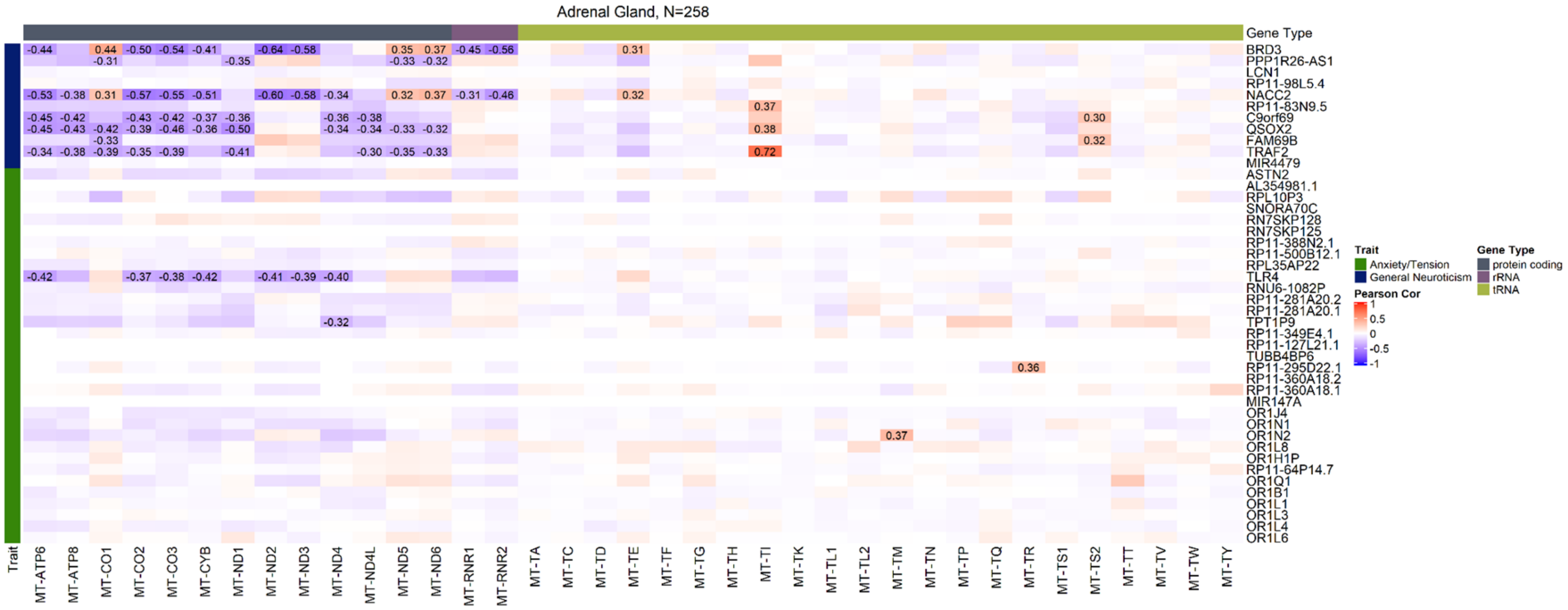

Colour in cells indicates the direction of a Pearson correlation and when labelled, indicate a statistically significant correlation at P < 0.05/56,200 (two-sided) where 56,200 is the total number of genes in GTEx v8. Rows are split by traits where general neuroticism is in dark blue and anxiety/tension is in green. Columns are split by gene type, where protein-coding is in grey, rRNA is in purple, and tRNA is in light green.

Supplementary Figure 8. Pearson correlations of gene expression between 37 MT genes and 11 genes mapped from the general neuroticism locus and 33 mapped genes from anxiety/tension locus on chromosome 9 1,204 GTEx v8 adipose tissue samples.

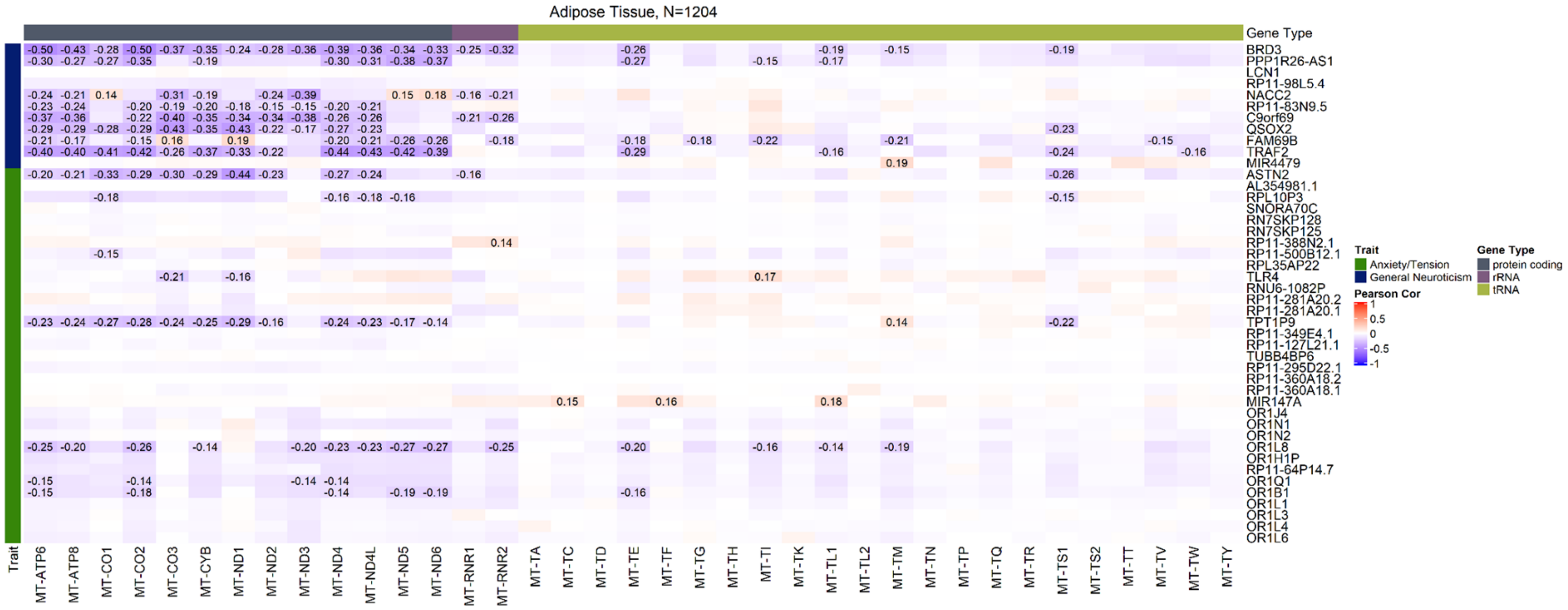

Colour in cells indicates the direction of a Pearson correlation and when labelled, indicate a statistically significant correlation at P < 0.05/56,200 (two-sided) where 56,200 is the total number of genes in GTEx v8. Rows are split by traits where general neuroticism is in dark blue and anxiety/tension is in green. Columns are split by gene type, where protein-coding is in grey, rRNA is in purple, and tRNA is in light green.

Supplementary Figure 9. Pearson correlations of gene expression between 37 MT genes and 11 genes mapped from the general neuroticism locus and 33 mapped genes from anxiety/tension locus on chromosome 9 1,335 GTEx v8 blood vessel tissue samples.

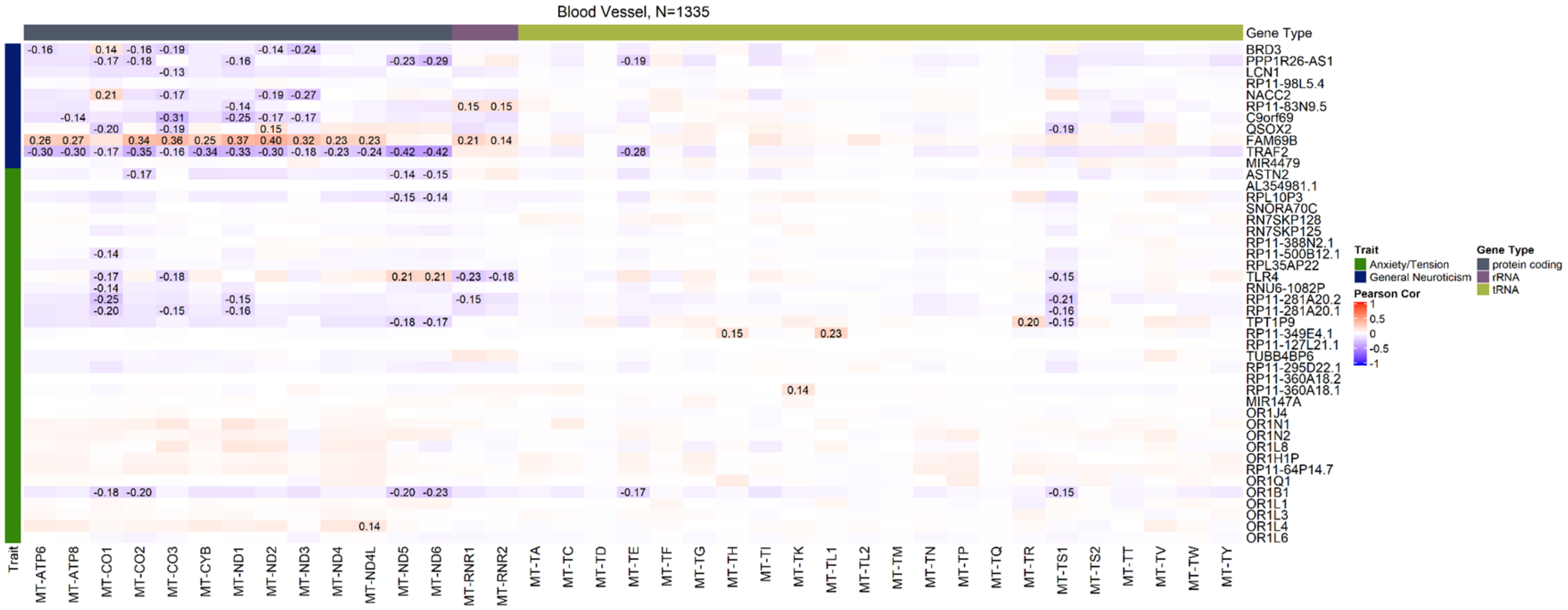

Colour in cells indicates the direction of a Pearson correlation and when labelled, indicate a statistically significant correlation at P < 0.05/56,200 (two-sided) where 56,200 is the total number of genes in GTEx v8. Rows are split by traits where general neuroticism is in red and anxiety/tension is in green. Columns are split by gene type, where protein-coding is in red, rRNA is in green, and tRNA is in blue.

Supplementary Figure 10. Pearson correlations of gene expression between 37 MT genes and 11 genes mapped from the general neuroticism locus and 33 mapped genes from anxiety/tension locus on chromosome 9 in 459 GTEx v8 breast tissue samples.

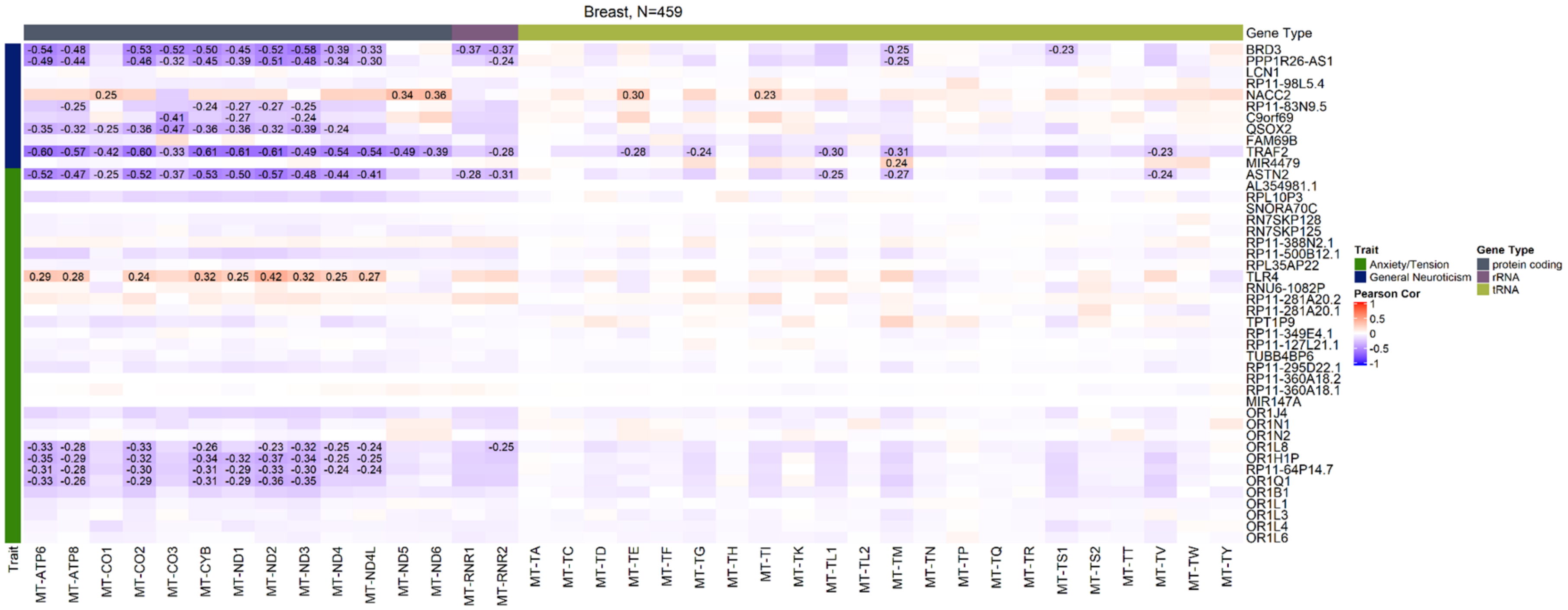

Colour in cells indicates the direction of a Pearson correlation and when labelled, indicate a statistically significant correlation at P < 0.05/56,200 (two-sided) where 56,200 is the total number of genes in GTEx v8. Rows are split by traits where general neuroticism is in dark blue and anxiety/tension is in green. Columns are split by gene type, where protein-coding is in grey, rRNA is in purple, and tRNA is in light green.

Supplementary Figure 11. Pearson correlations of gene expression between 37 MT genes and 11 genes mapped from the general neuroticism locus and 33 mapped genes from anxiety/tension locus on chromosome 9 in 779 GTEx v8 colon tissue samples.

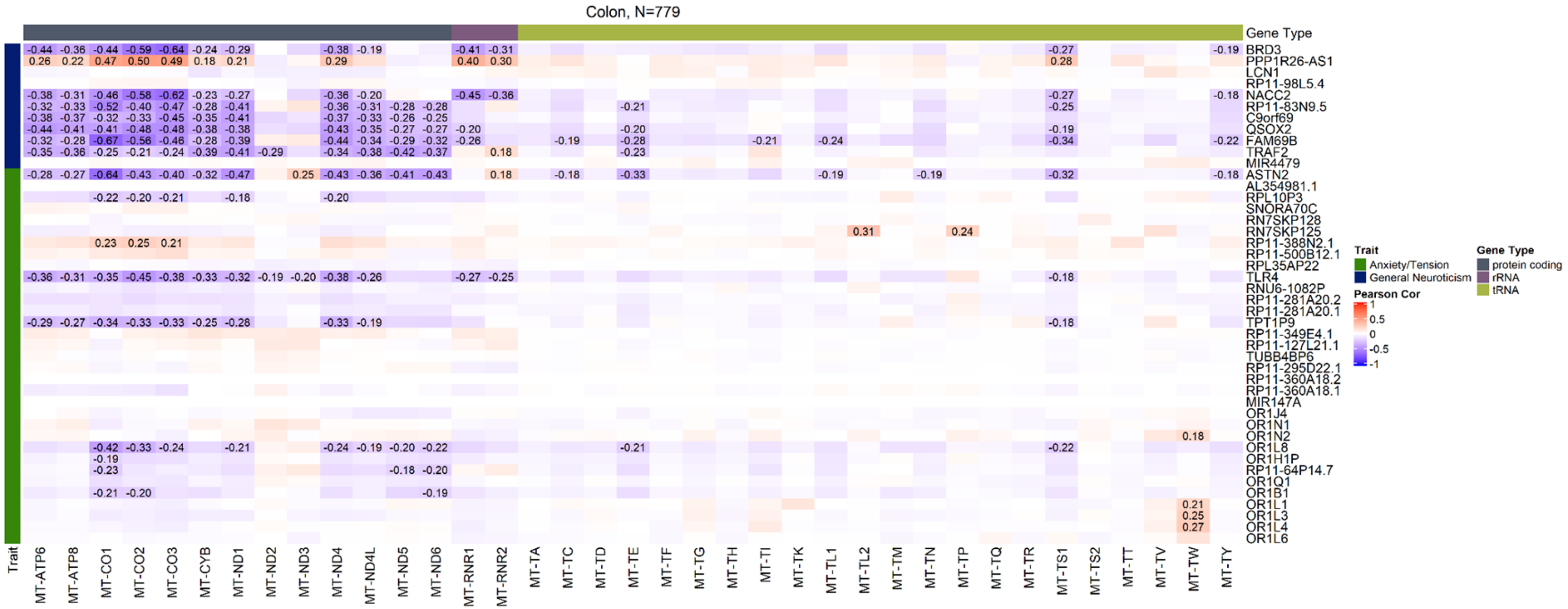

Colour in cells indicates the direction of a Pearson correlation and when labelled, indicate a statistically significant correlation at P < 0.05/56,200 (two-sided) where 56,200 is the total number of genes in GTEx v8. Rows are split by traits where general neuroticism is in dark blue and anxiety/tension is in green. Columns are split by gene type, where protein-coding is in grey, rRNA is in purple, and tRNA is in light green.

Supplementary Figure 12. Pearson correlations of gene expression between 37 MT genes and 11 genes mapped from the general neuroticism locus and 33 mapped genes from anxiety/tension locus on chromosome 9 in 1,445 GTEx v8 esophagus tissue samples.

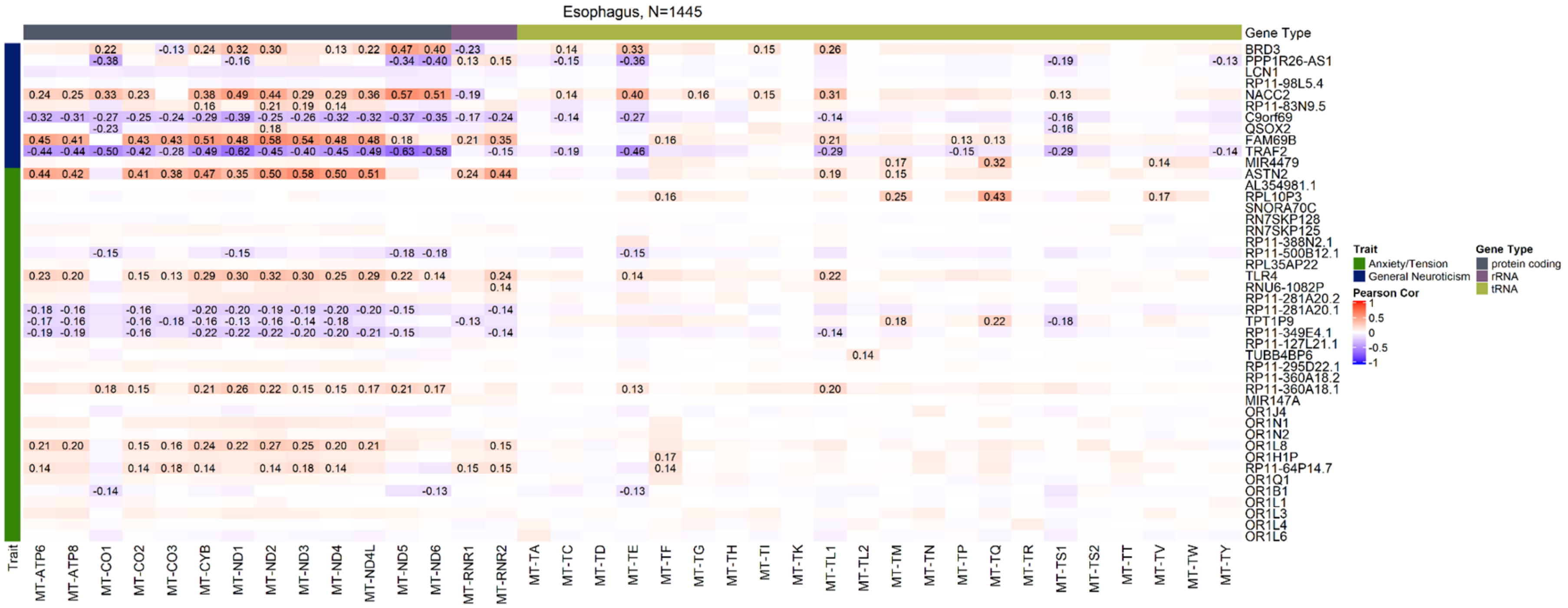

Colour in cells indicates the direction of a Pearson correlation and when labelled, indicate a statistically significant correlation at P < 0.05/56,200 (two-sided) where 56,200 is the total number of genes in GTEx v8. Rows are split by traits where general neuroticism is in dark blue and anxiety/tension is in green. Columns are split by gene type, where protein-coding is in grey, rRNA is in purple, and tRNA is in light green.

Supplementary Figure 13. Pearson correlations of gene expression between 37 MT genes and 11 genes mapped from the general neuroticism locus and 33 mapped genes from anxiety/tension locus on chromosome 9 in 861 GTEx v8 heart tissue samples.

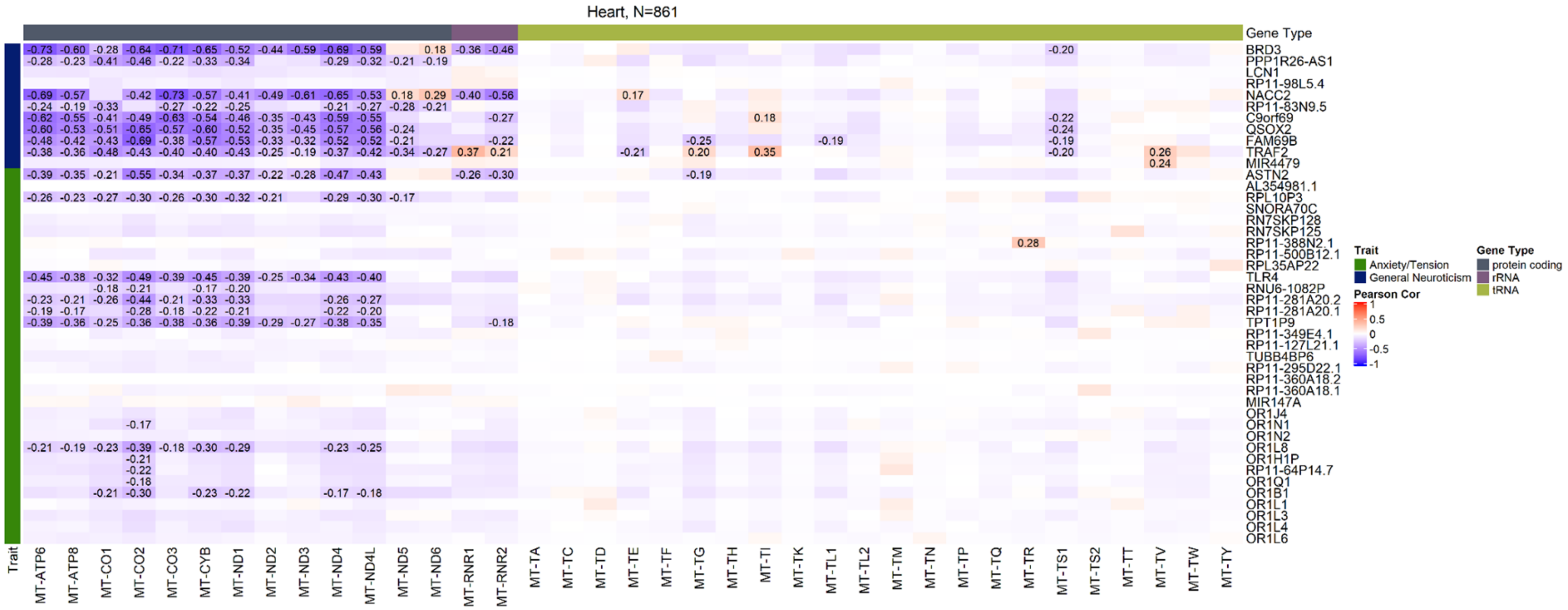

Colour in cells indicates the direction of a Pearson correlation and when labelled, indicate a statistically significant correlation at P < 0.05/56,200 (two-sided) where 56,200 is the total number of genes in GTEx v8. Rows are split by traits where general neuroticism is in dark blue and anxiety/tension is in green. Columns are split by gene type, where protein-coding is in grey, rRNA is in purple, and tRNA is in light green.

Supplementary Figure 14. Pearson correlations of gene expression between 37 MT genes and 11 genes mapped from the general neuroticism locus and 33 mapped genes from anxiety/tension locus on chromosome 9 in 89 GTEx v8 kidney tissue samples.

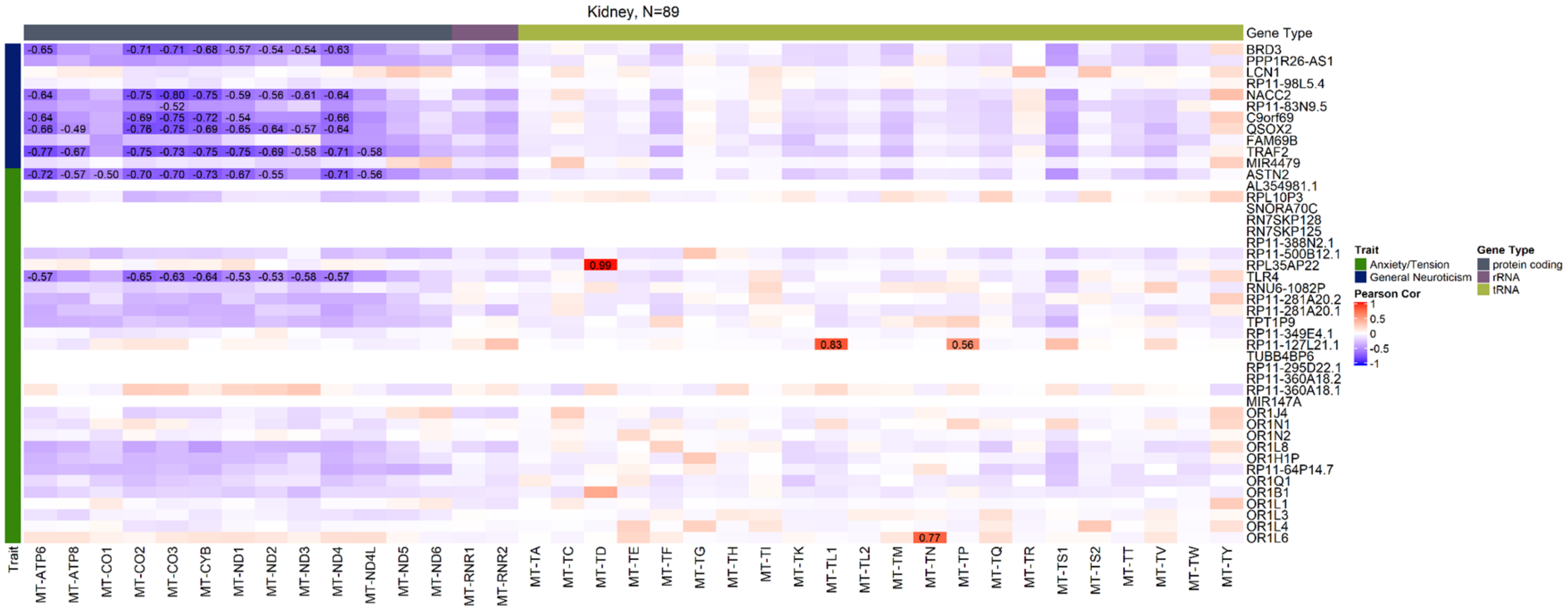

Colour in cells indicates the direction of a Pearson correlation and when labelled, indicate a statistically significant correlation at P < 0.05/56,200 (two-sided) where 56,200 is the total number of genes in GTEx v8. Rows are split by traits where general neuroticism is in dark blue and anxiety/tension is in green. Columns are split by gene type, where protein-coding is in grey, rRNA is in purple, and tRNA is in light green.

Supplementary Figure 15. Pearson correlations of gene expression between 37 MT genes and 11 genes mapped from the general neuroticism locus and 33 mapped genes from anxiety/tension locus on chromosome 9 in 226 GTEx v8 liver tissue samples.

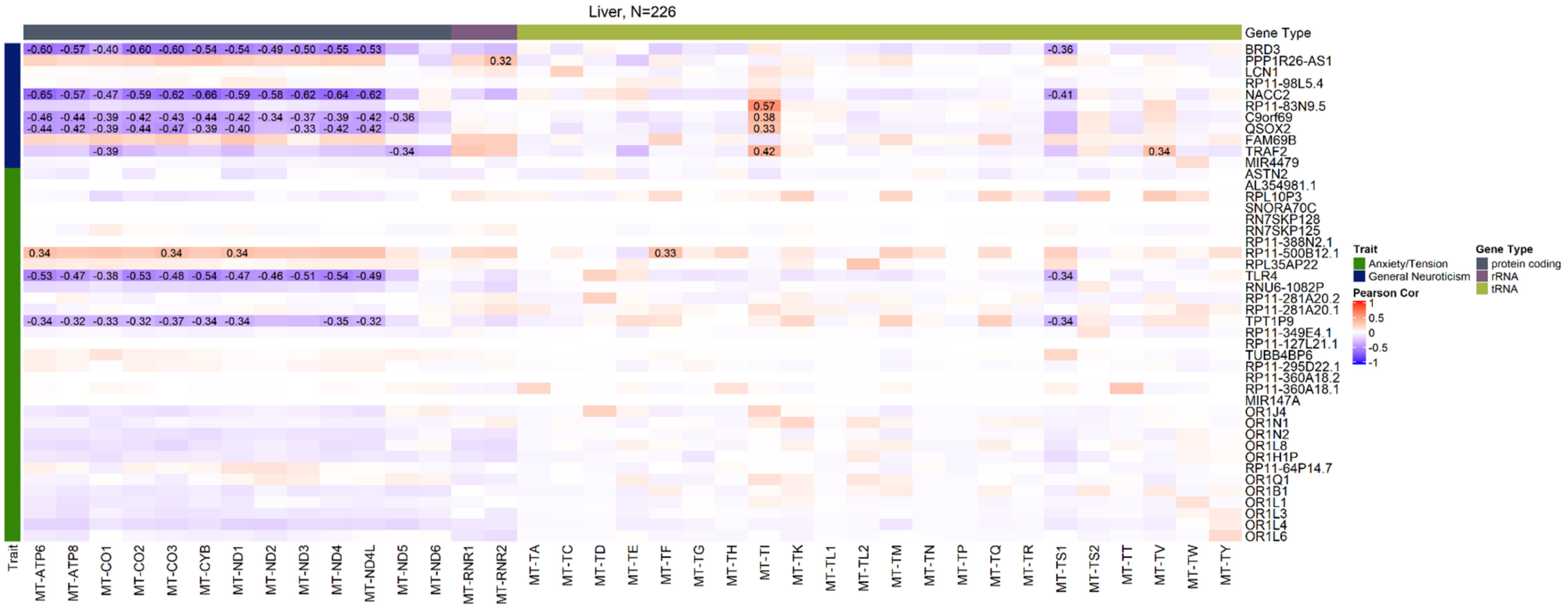

Colour in cells indicates the direction of a Pearson correlation and when labelled, indicate a statistically significant correlation at P < 0.05/56,200 (two-sided) where 56,200 is the total number of genes in GTEx v8. Rows are split by traits where general neuroticism is in dark blue and anxiety/tension is in green. Columns are split by gene type, where protein-coding is in grey, rRNA is in purple, and tRNA is in light green.

Supplementary Figure 16. Pearson correlations of gene expression between 37 MT genes and 11 genes mapped from the general neuroticism locus and 33 mapped genes from anxiety/tension locus on chromosome 9 in 578 GTEx v8 lung tissue samples.

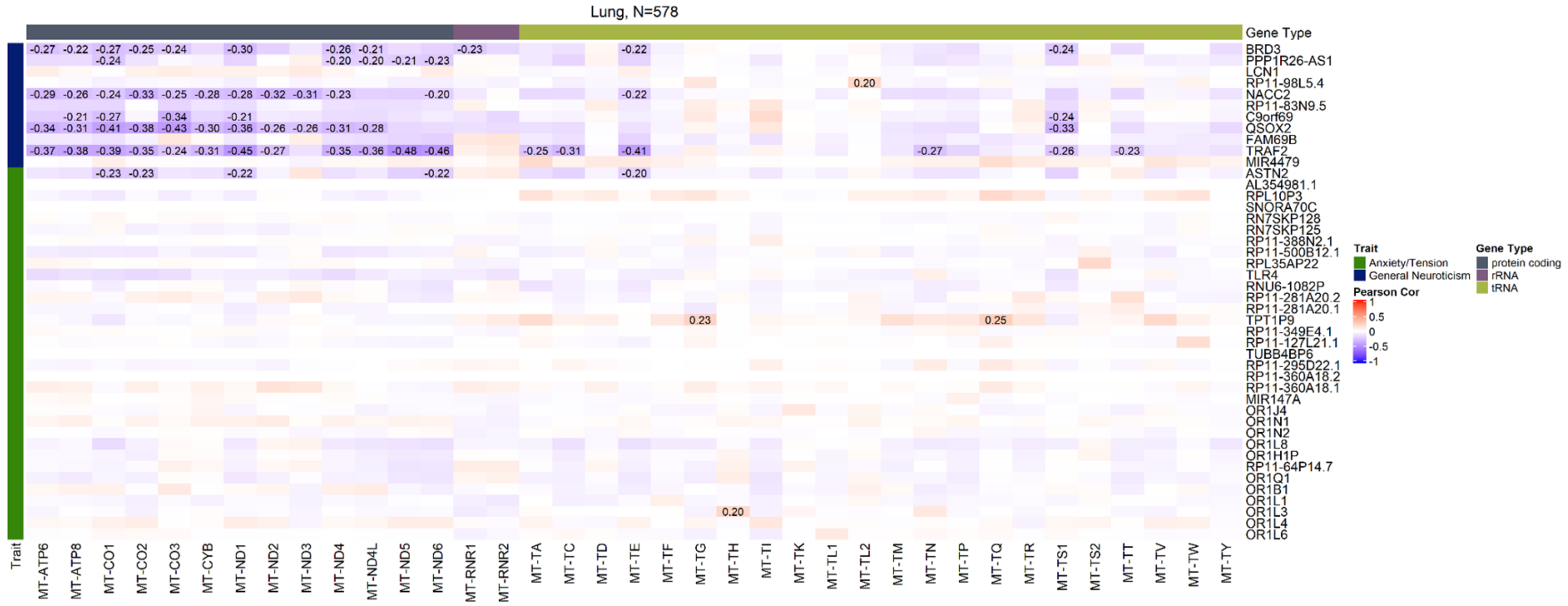

Colour in cells indicates the direction of a Pearson correlation and when labelled, indicate a statistically significant correlation at P < 0.05/56,200 (two-sided) where 56,200 is the total number of genes in GTEx v8. Rows are split by traits where general neuroticism is in dark blue and anxiety/tension is in green. Columns are split by gene type, where protein-coding is in grey, rRNA is in purple, and tRNA is in light green.

Supplementary Figure 17. Pearson correlations of gene expression between 37 MT genes and 11 genes mapped from the general neuroticism locus and 33 mapped genes from anxiety/tension locus on chromosome 9 in 803 GTEx v8 muscle tissue samples.

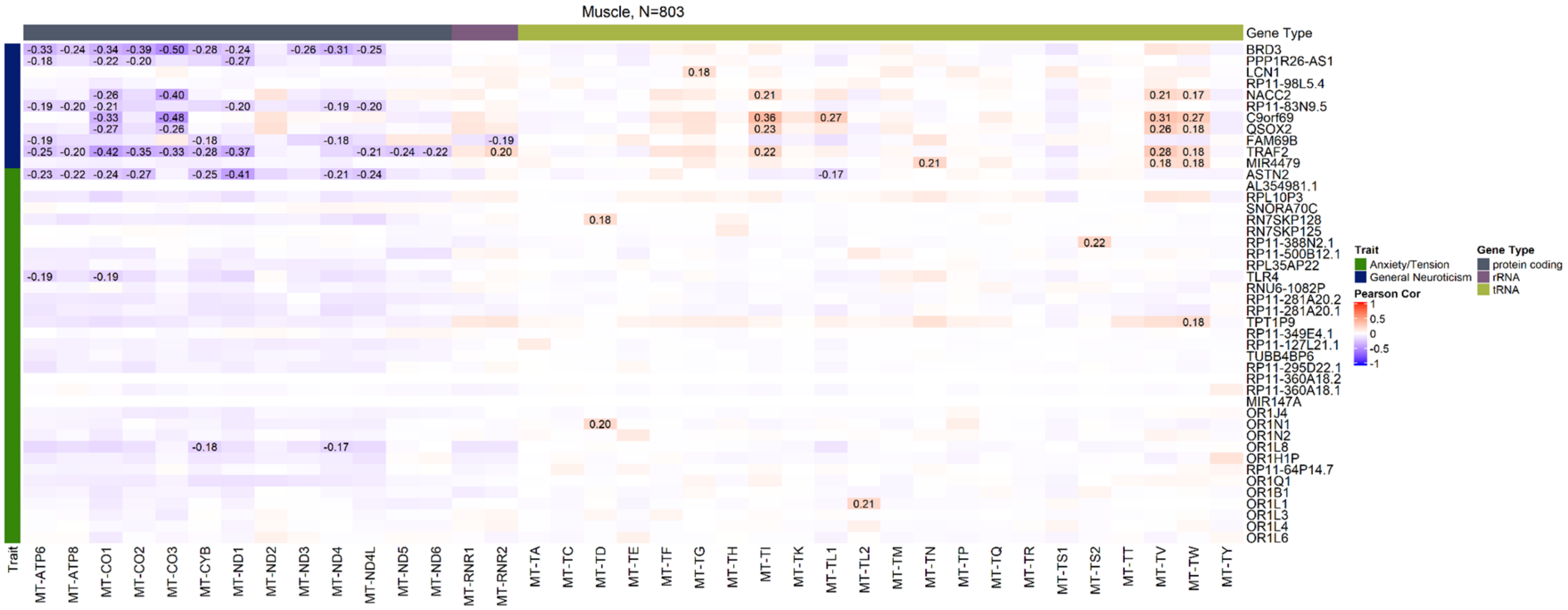

Colour in cells indicates the direction of a Pearson correlation and when labelled, indicate a statistically significant correlation at P < 0.05/56,200 (two-sided) where 56,200 is the total number of genes in GTEx v8. Rows are split by traits where general neuroticism is in dark blue and anxiety/tension is in green. Columns are split by gene type, where protein-coding is in grey, rRNA is in purple, and tRNA is in light green.

Supplementary Figure 18. Pearson correlations of gene expression between 37 MT genes and 11 genes mapped from the general neuroticism locus and 33 mapped genes from anxiety/tension locus on chromosome 9 in 619 GTEx v8 nerve tissue samples.

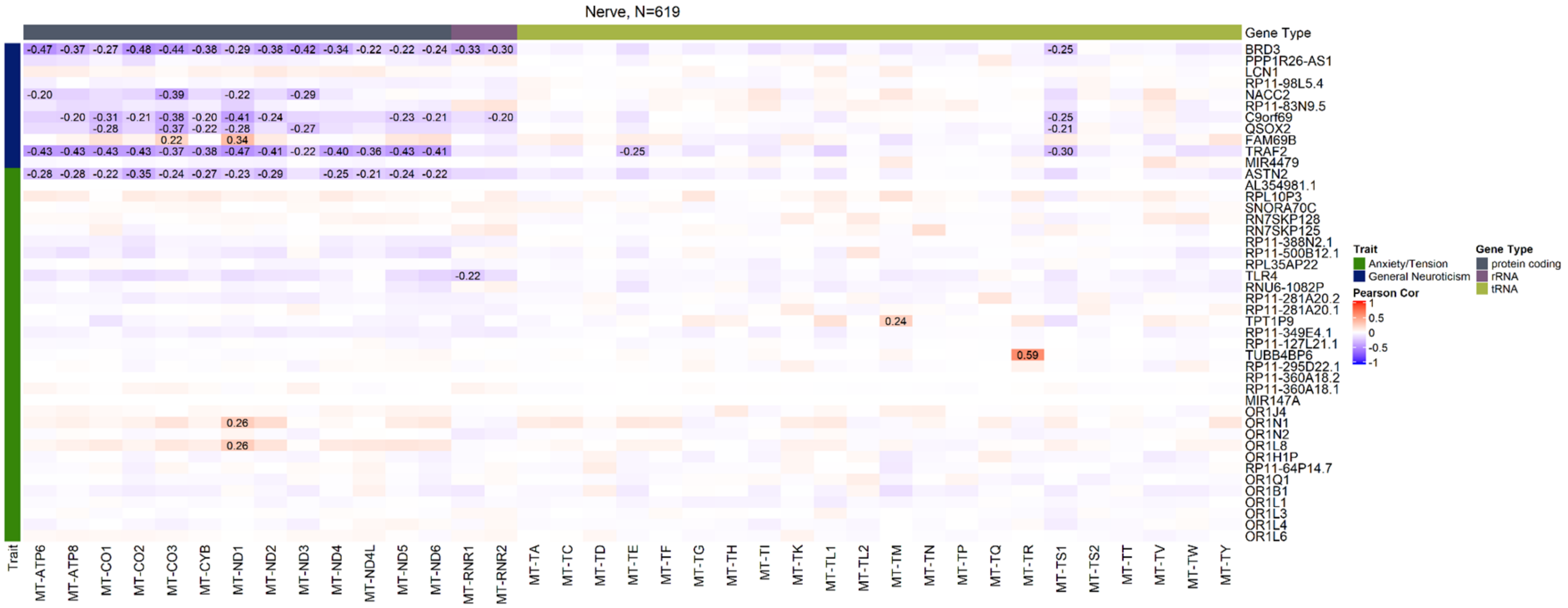

Colour in cells indicates the direction of a Pearson correlation and when labelled, indicate a statistically significant correlation at P < 0.05/56,200 (two-sided) where 56,200 is the total number of genes in GTEx v8. Rows are split by traits where general neuroticism is in dark blue and anxiety/tension is in green. Columns are split by gene type, where protein-coding is in grey, rRNA is in purple, and tRNA is in light green.

Supplementary Figure 19. Pearson correlations of gene expression between 37 MT genes and 11 genes mapped from the general neuroticism locus and 33 mapped genes from anxiety/tension locus on chromosome 9 in 180 GTEx v8 ovary tissue samples.

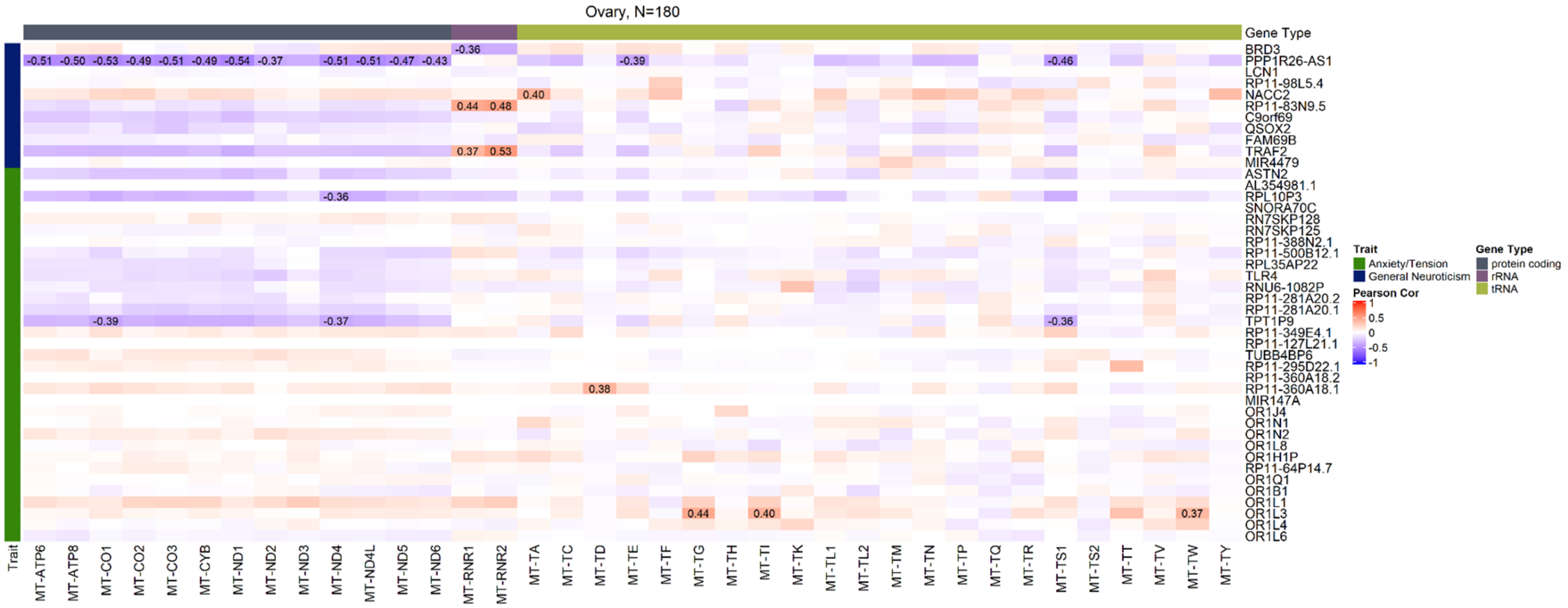

Colour in cells indicates the direction of a Pearson correlation and when labelled, indicate a statistically significant correlation at P < 0.05/56,200 (two-sided) where 56,200 is the total number of genes in GTEx v8. Rows are split by traits where general neuroticism is in dark blue and anxiety/tension is in green. Columns are split by gene type, where protein-coding is in grey, rRNA is in purple, and tRNA is in light green.

Supplementary Figure 20. Pearson correlations of gene expression between 37 MT genes and 11 genes mapped from the general neuroticism locus and 33 mapped genes from anxiety/tension locus on chromosome 9 in 328 GTEx v8 pancreas tissue samples.

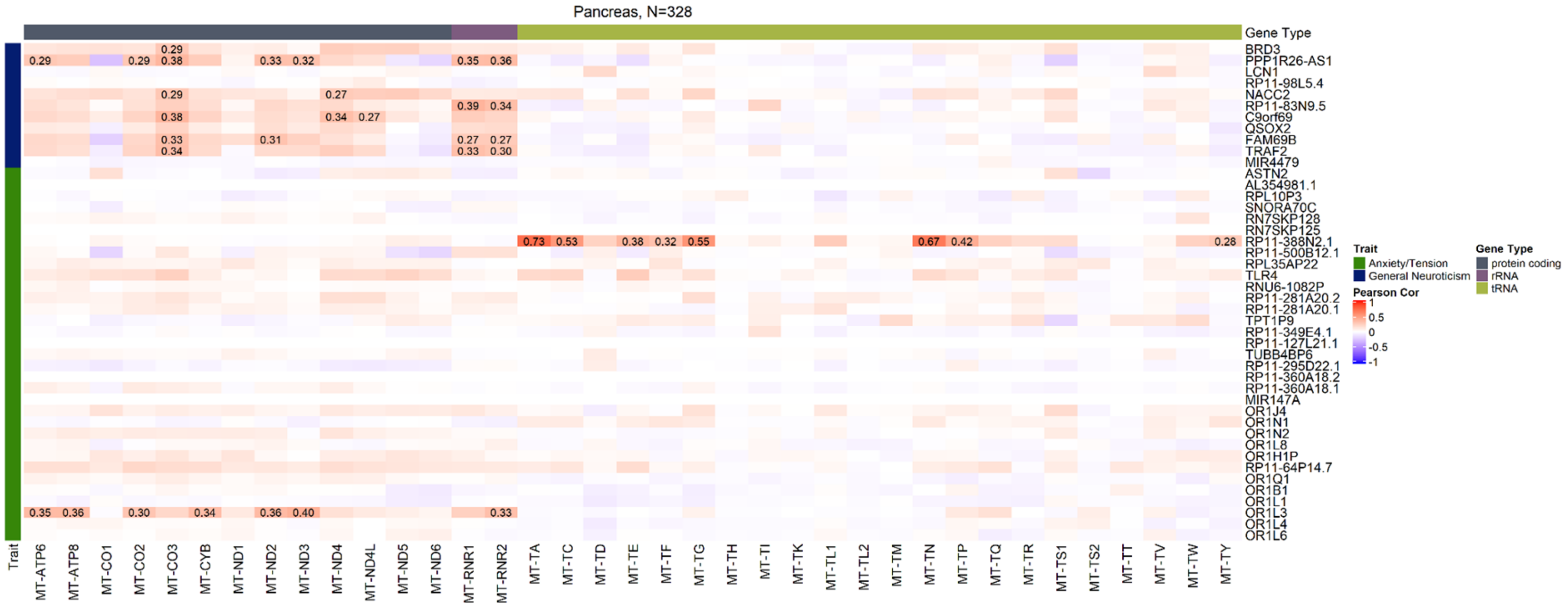

Colour in cells indicates the direction of a Pearson correlation and when labelled, indicate a statistically significant correlation at $P < 0.05/56,200$ (two-sided) where 56,200 is the total number of genes in GTEx v8. Rows are split by traits where general neuroticism is in dark blue and anxiety/tension is in green. Columns are split by gene type, where protein-coding is in grey, rRNA is in purple, and tRNA is in light green.

Supplementary Figure 21. Pearson correlations of gene expression between 37 MT genes and 11 genes mapped from the general neuroticism locus and 33 mapped genes from anxiety/tension locus on chromosome 9 in 283 GTEx v8 pituitary tissue samples.

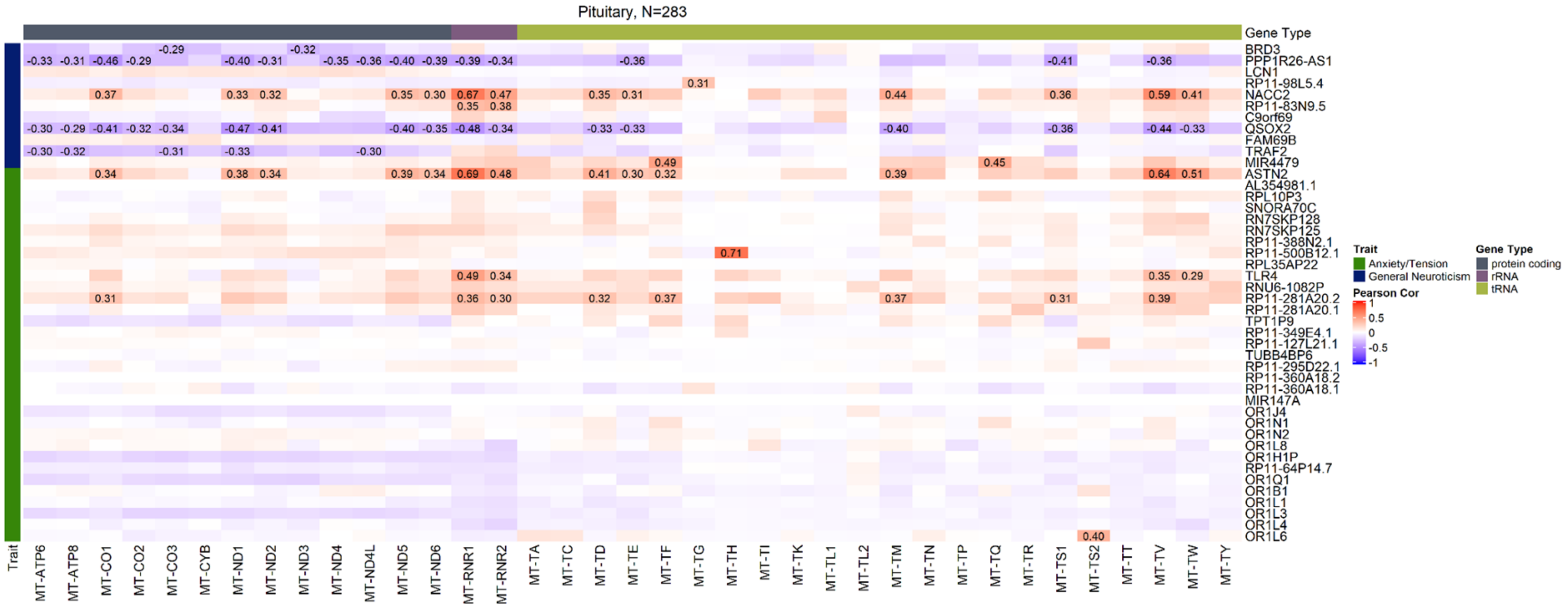

Colour in cells indicates the direction of a Pearson correlation and when labelled, indicate a statistically significant correlation at P < 0.05/56,200 (two-sided) where 56,200 is the total number of genes in GTEx v8. Rows are split by traits where general neuroticism is in dark blue and anxiety/tension is in green. Columns are split by gene type, where protein-coding is in grey, rRNA is in purple, and tRNA is in light green.

Supplementary Figure 22. Pearson correlations of gene expression between 37 MT genes and 11 genes mapped from the general neuroticism locus and 33 mapped genes from anxiety/tension locus on chromosome 9 in 245 GTEx v8 prostate tissue samples.

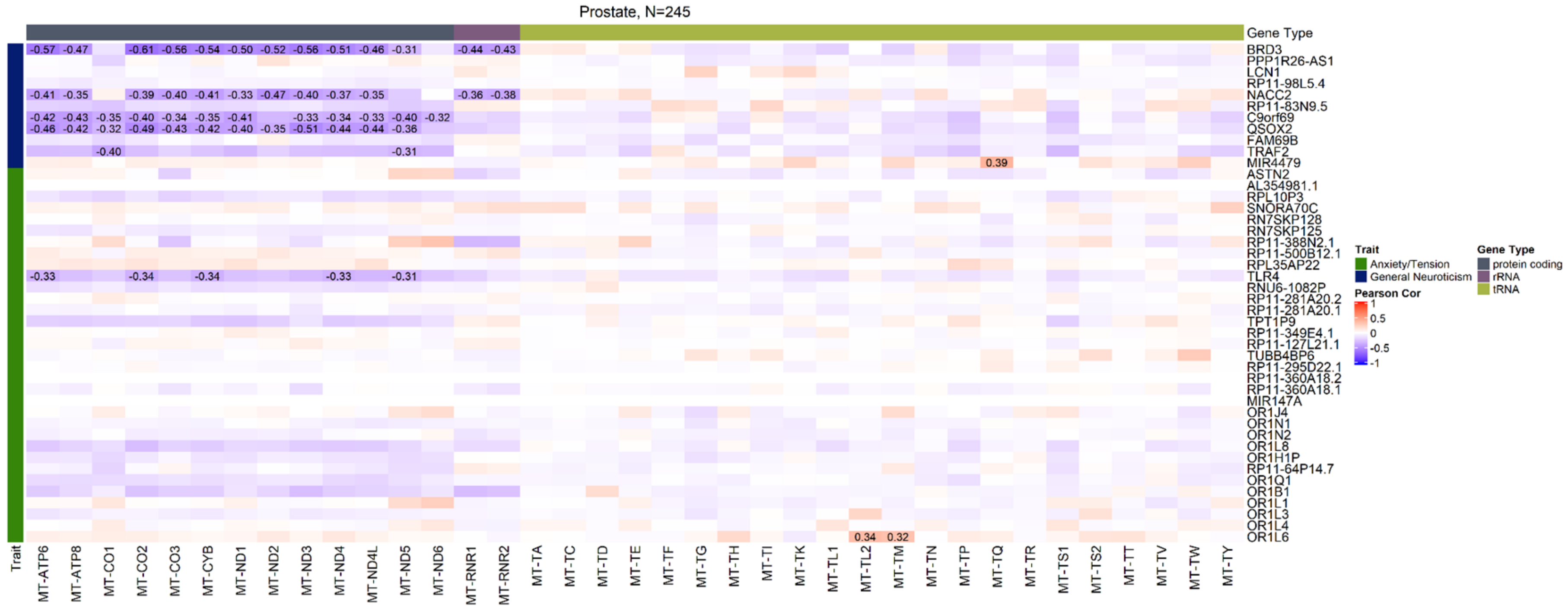

Colour in cells indicates the direction of a Pearson correlation and when labelled, indicate a statistically significant correlation at P < 0.05/56,200 (two-sided) where 56,200 is the total number of genes in GTEx v8. Rows are split by traits where general neuroticism is in dark blue and anxiety/tension is in green. Columns are split by gene type, where protein-coding is in grey, rRNA is in purple, and tRNA is in light green.

Supplementary Figure 23. Pearson correlations of gene expression between 37 MT genes and 11 genes mapped from the general neuroticism locus and 33 mapped genes from anxiety/tension locus on chromosome 9 in 162 GTEx v8 salivary gland tissue samples.

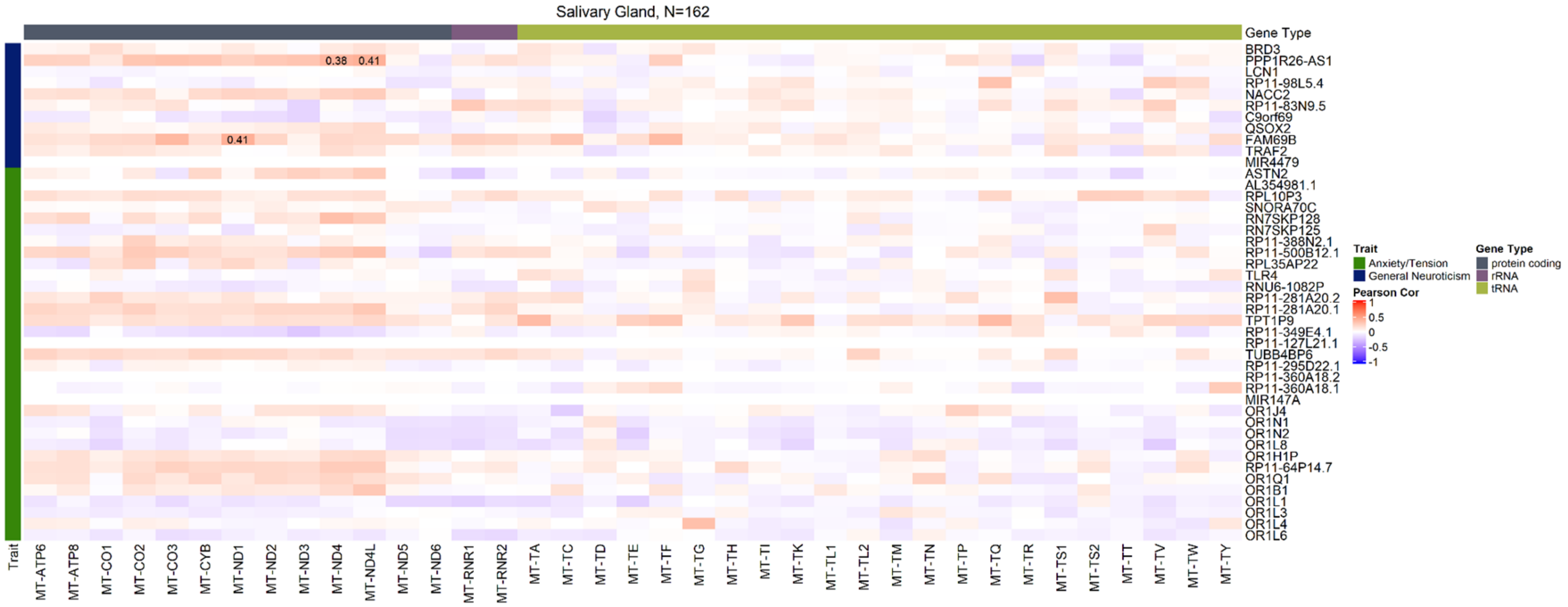

Colour in cells indicates the direction of a Pearson correlation and when labelled, indicate a statistically significant correlation at P < 0.05/56,200 (two-sided) where 56,200 is the total number of genes in GTEx v8. Rows are split by traits where general neuroticism is in dark blue and anxiety/tension is in green. Columns are split by gene type, where protein-coding is in grey, rRNA is in purple, and tRNA is in light green.

Supplementary Figure 24. Pearson correlations of gene expression between 37 MT genes and 11 genes mapped from the general neuroticism locus and 33 mapped genes from anxiety/tension locus on chromosome 9 in 1,809 GTEx v8 skin tissue samples.

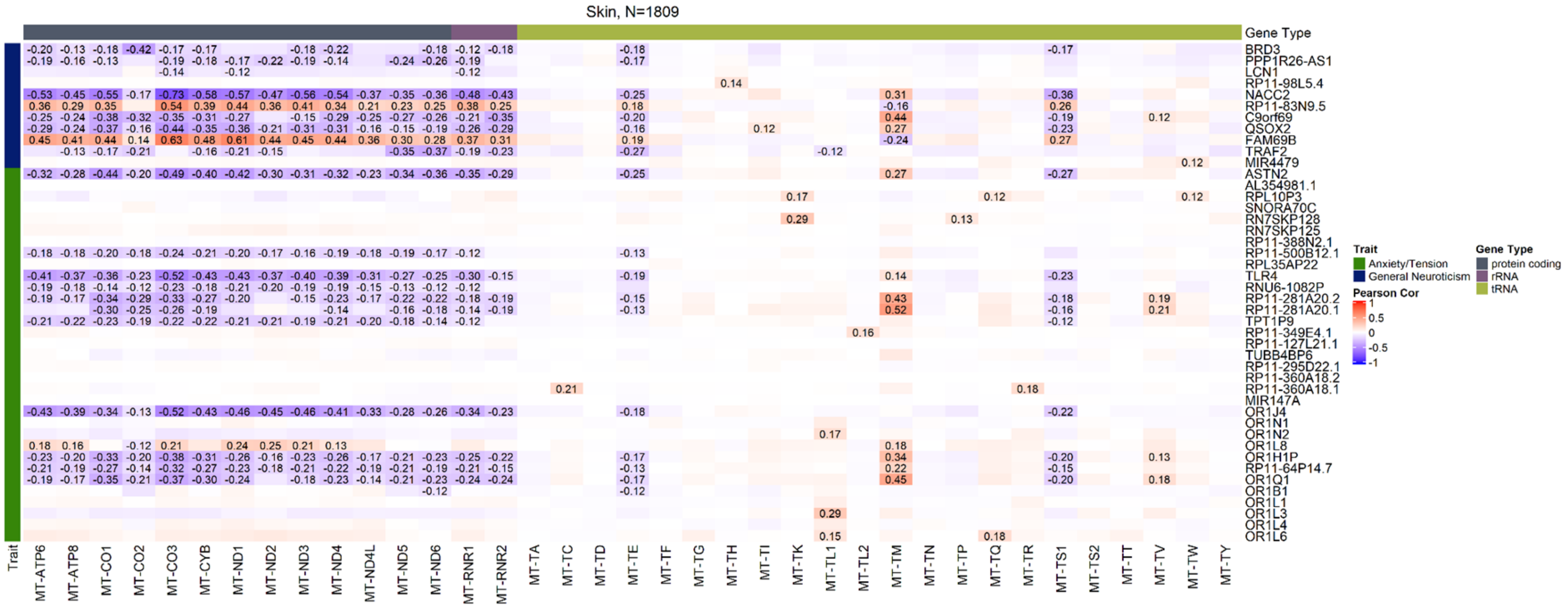

Colour in cells indicates the direction of a Pearson correlation and when labelled, indicate a statistically significant correlation at P < 0.05/56,200 (two-sided) where 56,200 is the total number of genes in GTEx v8. Rows are split by traits where general neuroticism is in dark blue and anxiety/tension is in green. Columns are split by gene type, where protein-coding is in grey, rRNA is in purple, and tRNA is in light green.

Supplementary Figure 25. Pearson correlations of gene expression between 37 MT genes and 11 genes mapped from the general neuroticism locus and 33 mapped genes from anxiety/tension locus on chromosome 9 in 187 GTEx v8 small intestine tissue samples.

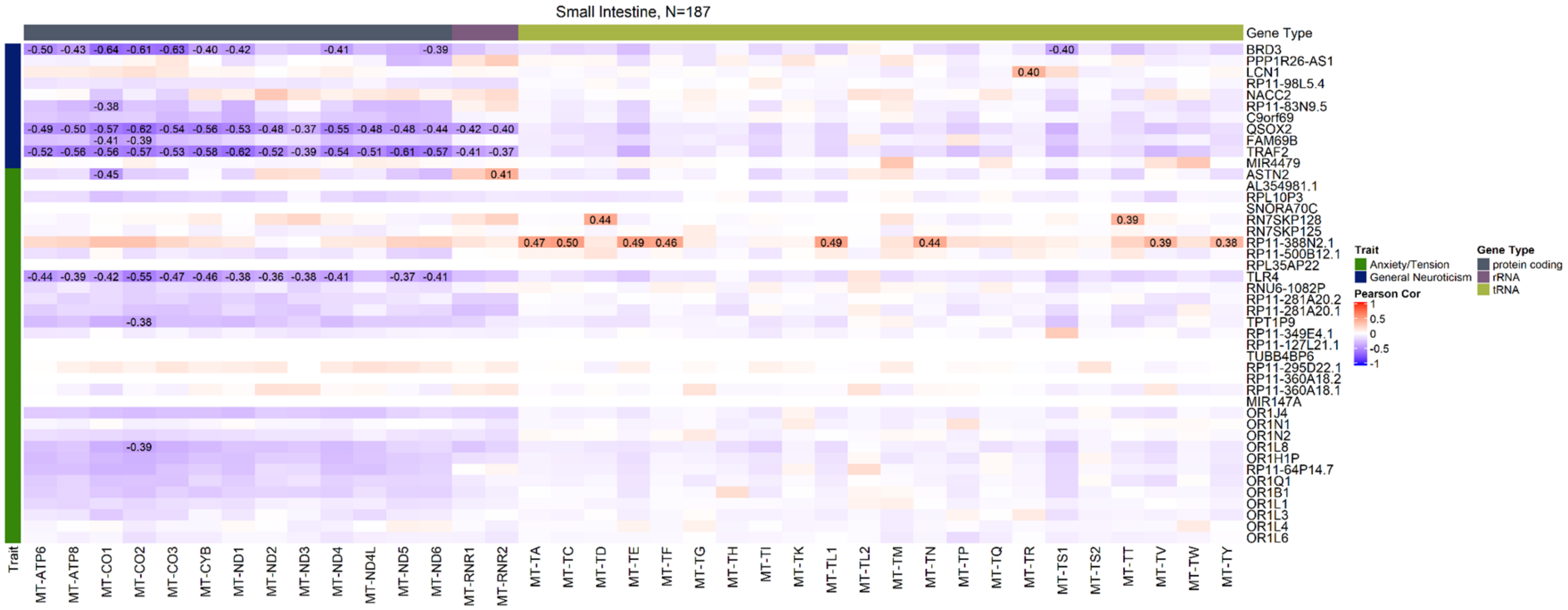

Colour in cells indicates the direction of a Pearson correlation and when labelled, indicate a statistically significant correlation at P < 0.05/56,200 (two-sided) where 56,200 is the total number of genes in GTEx v8. Rows are split by traits where general neuroticism is in dark blue and anxiety/tension is in green. Columns are split by gene type, where protein-coding is in grey, rRNA is in purple, and tRNA is in light green.

Supplementary Figure 26. Pearson correlations of gene expression between 37 MT genes and 11 genes mapped from the general neuroticism locus and 33 mapped genes from anxiety/tension locus on chromosome 9 in 241 GTEx v8 spleen tissue samples.

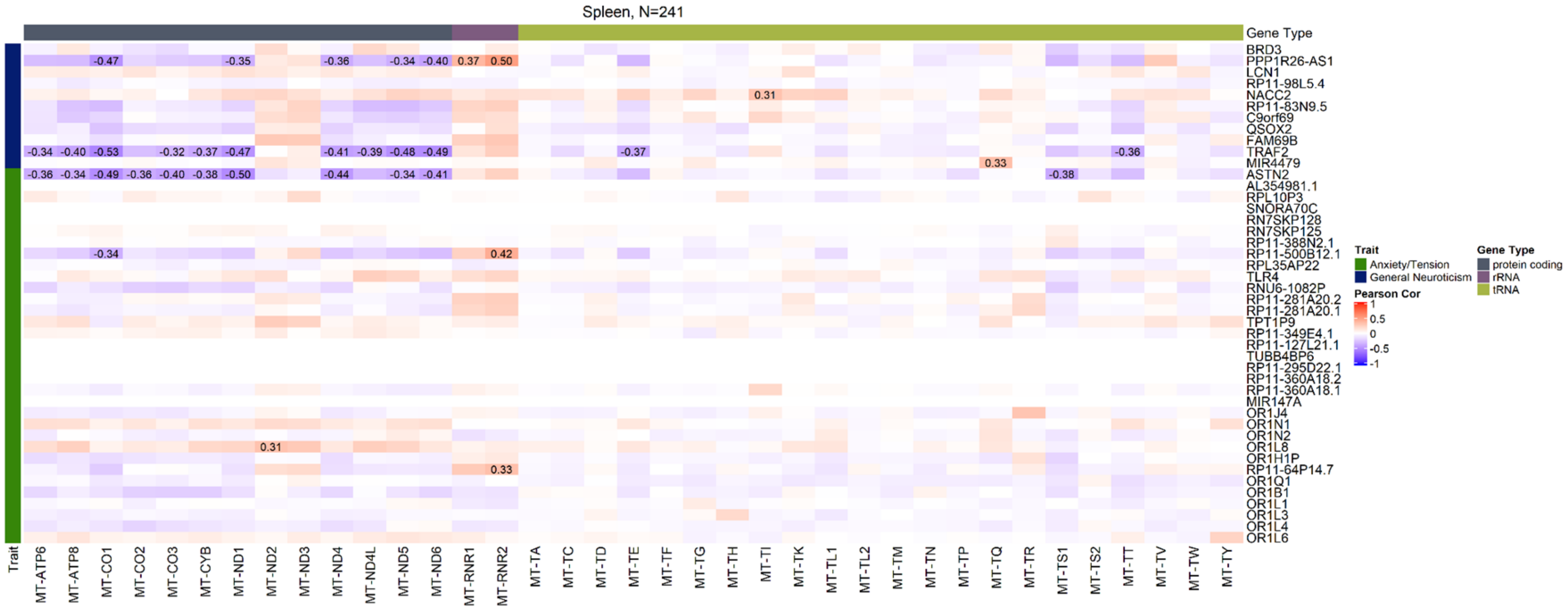

Colour in cells indicates the direction of a Pearson correlation and when labelled, indicate a statistically significant correlation at P < 0.05/56,200 (two-sided) where 56,200 is the total number of genes in GTEx v8. Rows are split by traits where general neuroticism is in dark blue and anxiety/tension is in green. Columns are split by gene type, where protein-coding is in grey, rRNA is in purple, and tRNA is in light green.

Supplementary Figure 27. Pearson correlations of gene expression between 37 MT genes and 11 genes mapped from the general neuroticism locus and 33 mapped genes from anxiety/tension locus on chromosome 9 in 359 GTEx v8 stomach tissue samples.

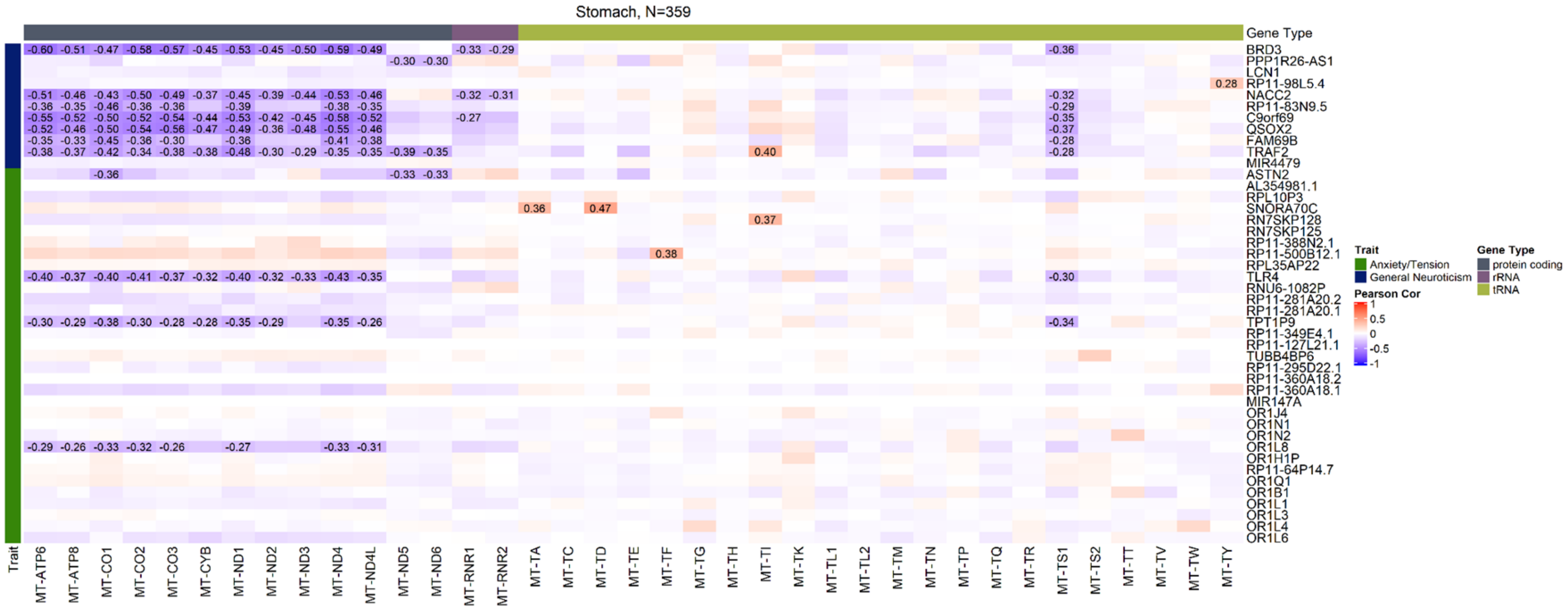

Colour in cells indicates the direction of a Pearson correlation and when labelled, indicate a statistically significant correlation at P < 0.05/56,200 (two-sided) where 56,200 is the total number of genes in GTEx v8. Rows are split by traits where general neuroticism is in dark blue and anxiety/tension is in green. Columns are split by gene type, where protein-coding is in grey, rRNA is in purple, and tRNA is in light green.

Supplementary Figure 28. Pearson correlations of gene expression between 37 MT genes and 11 genes mapped from the general neuroticism locus and 33 mapped genes from anxiety/tension locus on chromosome 9 in 361 GTEx v8 testis tissue samples.

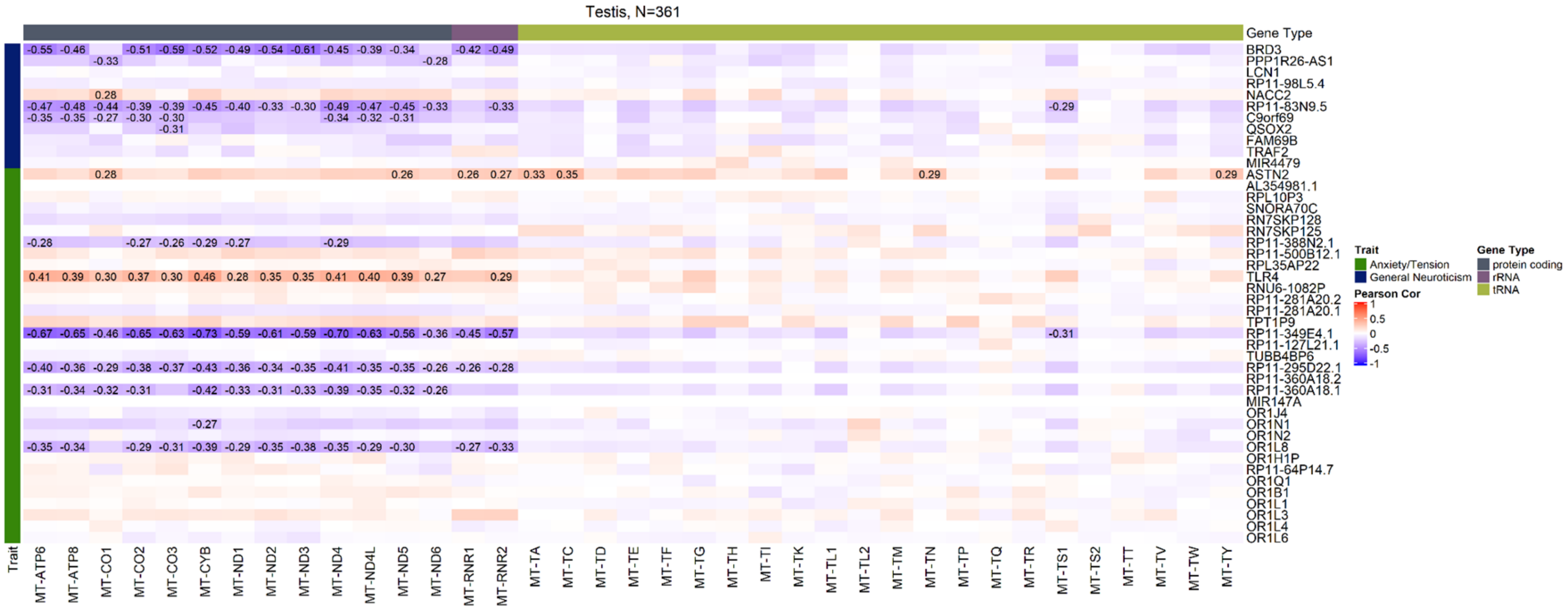

Colour in cells indicates the direction of a Pearson correlation and when labelled, indicate a statistically significant correlation at P < 0.05/56,200 (two-sided) where 56,200 is the total number of genes in GTEx v8. Rows are split by traits where general neuroticism is in dark blue and anxiety/tension is in green. Columns are split by gene type, where protein-coding is in grey, rRNA is in purple, and tRNA is in light green.

Supplementary Figure 29. Pearson correlations of gene expression between 37 MT genes and 11 genes mapped from the general neuroticism locus and 33 mapped genes from anxiety/tension locus on chromosome 9 in 653 GTEx v8 thyroid tissue samples.

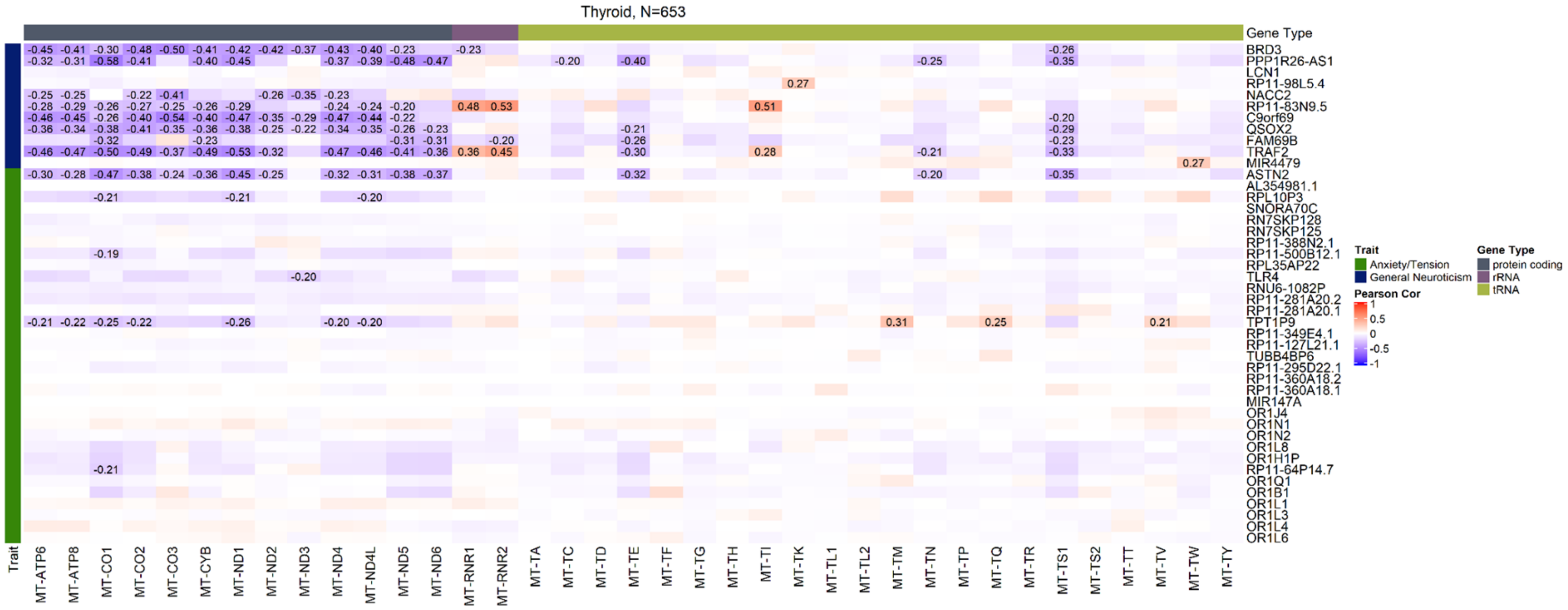

Colour in cells indicates the direction of a Pearson correlation and when labelled, indicate a statistically significant correlation at P < 0.05/56,200 (two-sided) where 56,200 is the total number of genes in GTEx v8. Rows are split by traits where general neuroticism is in dark blue and anxiety/tension is in green. Columns are split by gene type, where protein-coding is in grey, rRNA is in purple, and tRNA is in light green.

Supplementary Figure 30. Pearson correlations of gene expression between 37 MT genes and 11 genes mapped from the general neuroticism locus and 33 mapped genes from anxiety/tension locus on chromosome 9 in 142 GTEx v8 uterus tissue samples.

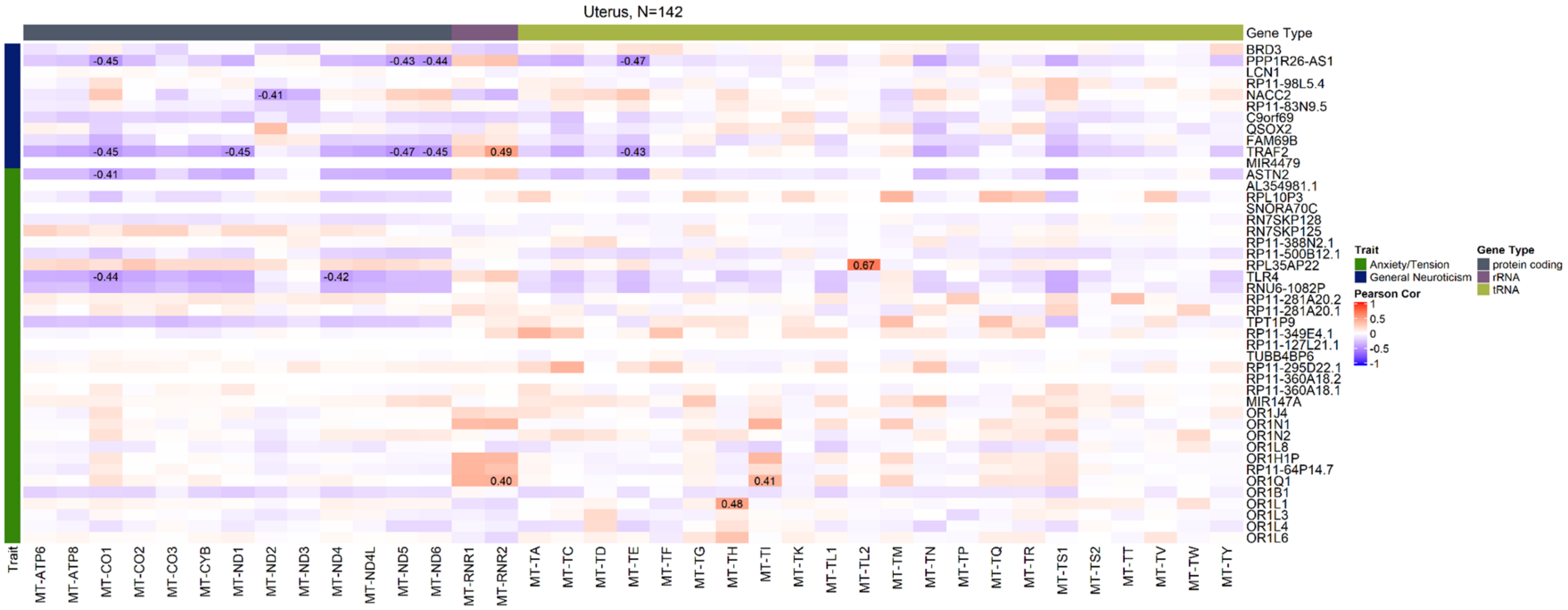

Colour in cells indicates the direction of a Pearson correlation and when labelled, indicate a statistically significant correlation at P < 0.05/56,200 (two-sided) where 56,200 is the total number of genes in GTEx v8. Rows are split by traits where general neuroticism is in dark blue and anxiety/tension is in green. Columns are split by gene type, where protein-coding is in grey, rRNA is in purple, and tRNA is in light green.

Supplementary Figure 31. Pearson correlations of gene expression between 37 MT genes and 11 genes mapped from the general neuroticism locus and 33 mapped genes from anxiety/tension locus on chromosome 9 in 156 GTEx v8 vagina tissue samples.

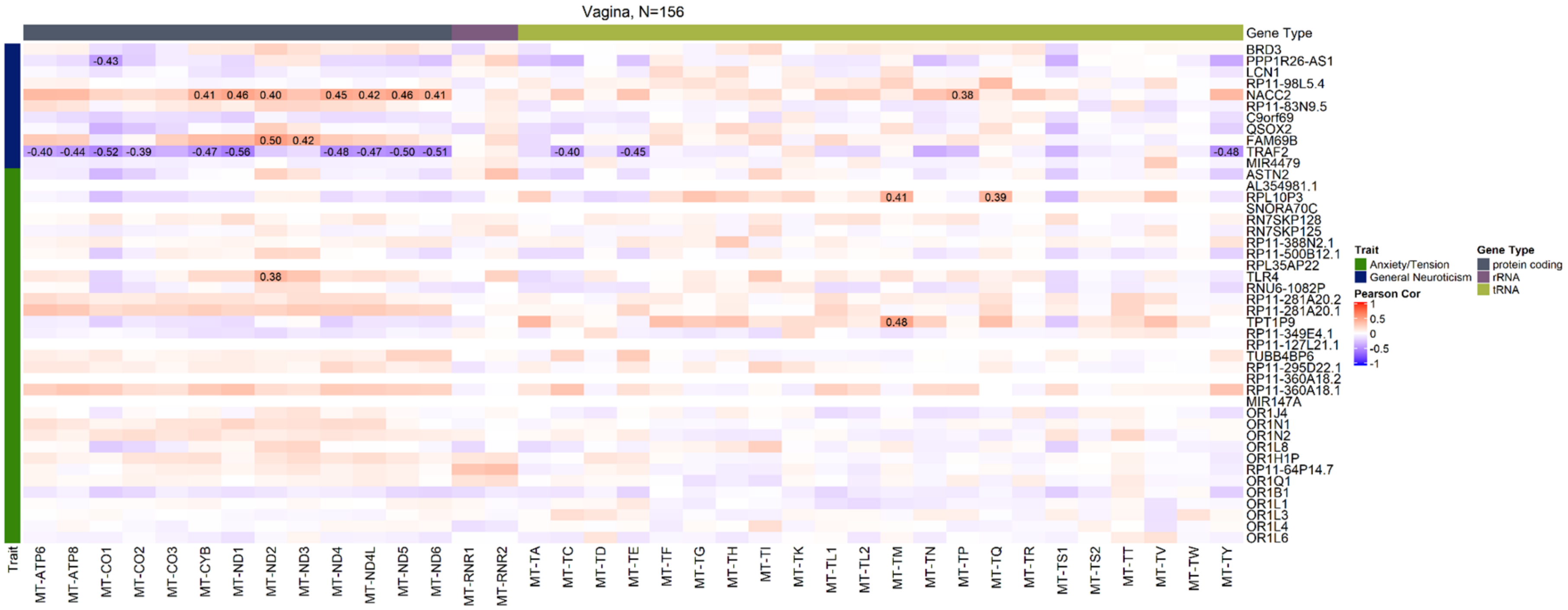

Colour in cells indicates the direction of a Pearson correlation and when labelled, indicate a statistically significant correlation at P < 0.05/56,200 (two-sided) where 56,200 is the total number of genes in GTEx v8. Rows are split by traits where general neuroticism is in dark blue and anxiety/tension is in green. Columns are split by gene type, where protein-coding is in grey, rRNA is in purple, and tRNA is in light green.

Supplementary Figure 32. Pearson correlations of gene expression between 37 MT genes and 11 genes mapped from the general neuroticism locus and 33 mapped genes from anxiety/tension locus on chromosome 9 in 152 GTEx v8 brain - amygdala tissue samples.

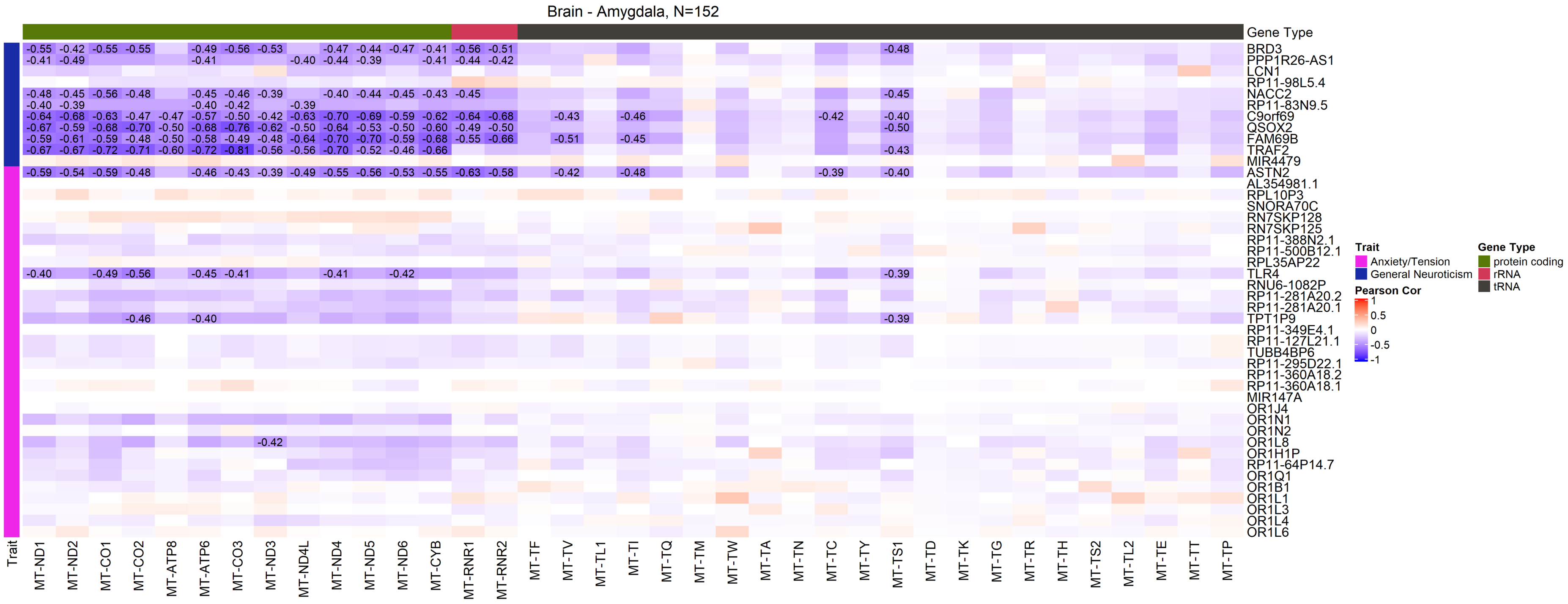

Colour in cells indicates the direction of a Pearson correlation and when labelled, indicate a statistically significant correlation at P < 0.05/56,200 (two-sided) where 56,200 is the total number of genes in GTEx v8. Rows are split by traits where general neuroticism is in dark blue and anxiety/tension is in green. Columns are split by gene type, where protein-coding is in grey, rRNA is in purple, and tRNA is in light green.

Supplementary Figure 33. Pearson correlations of gene expression between 37 MT genes and 11 genes mapped from the general neuroticism locus and 33 mapped genes from anxiety/tension locus on chromosome 9 in 176 GTEx v8 brain – anterior cingulate cortex tissue samples.

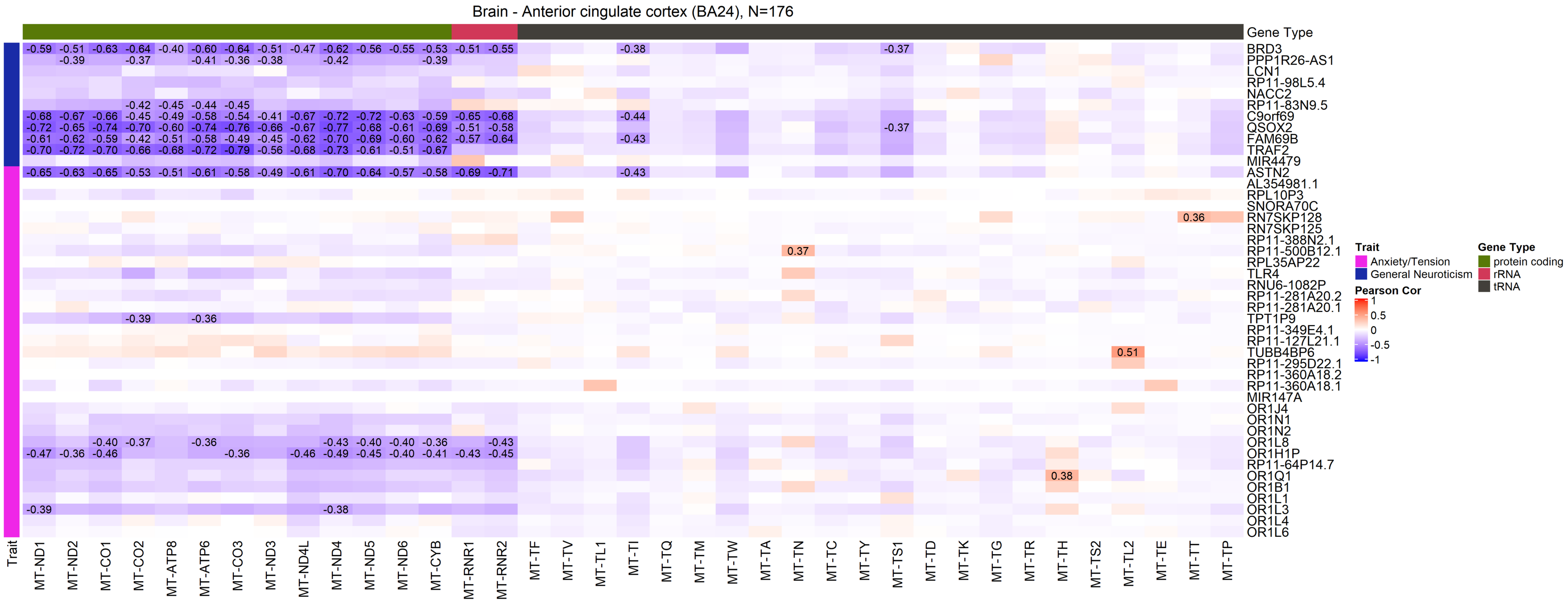

Colour in cells indicates the direction of a Pearson correlation and when labelled, indicate a statistically significant correlation at P < 0.05/56,200 (two-sided) where 56,200 is the total number of genes in GTEx v8. Rows are split by traits where general neuroticism is in dark blue and anxiety/tension is in green. Columns are split by gene type, where protein-coding is in grey, rRNA is in purple, and tRNA is in light green.

Supplementary Figure 34. Pearson correlations of gene expression between 37 MT genes and 11 genes mapped from the general neuroticism locus and 33 mapped genes from anxiety/tension locus on chromosome 9 in 246 GTEx v8 brain - caudate tissue samples.

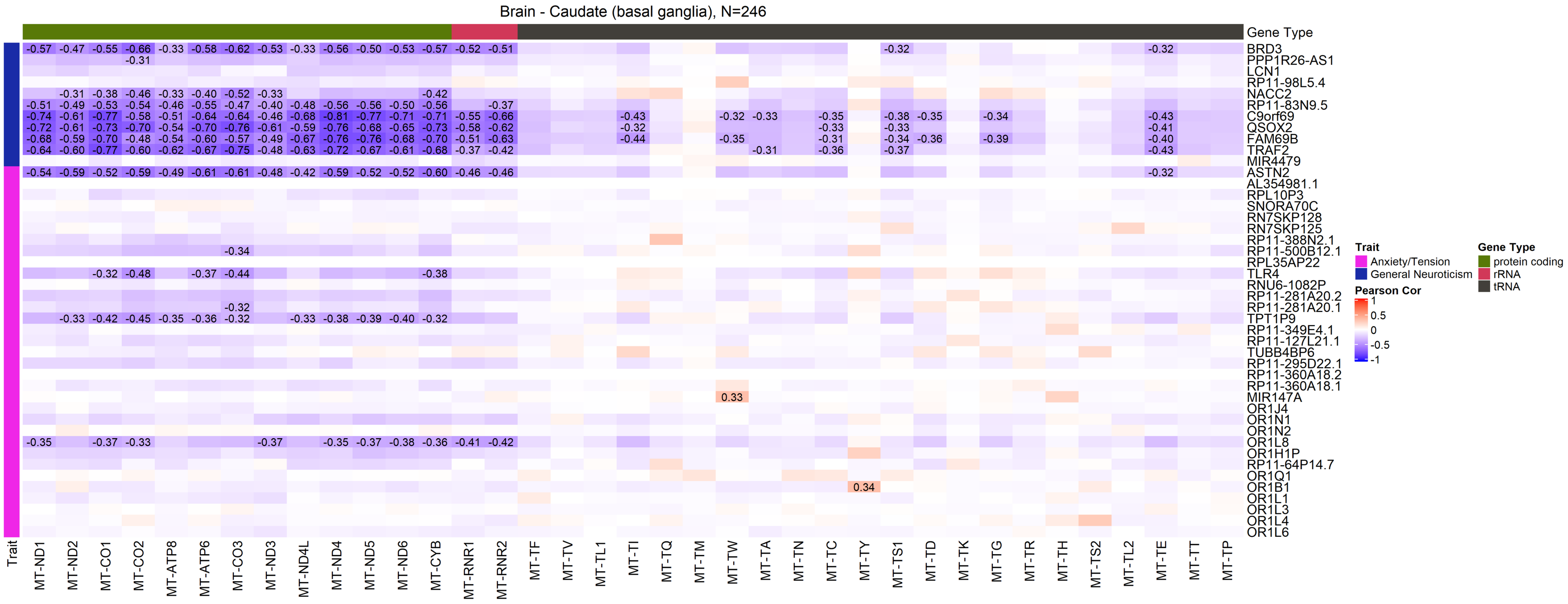

Colour in cells indicates the direction of a Pearson correlation and when labelled, indicate a statistically significant correlation at P < 0.05/56,200 (two-sided) where 56,200 is the total number of genes in GTEx v8. Rows are split by traits where general neuroticism is in dark blue and anxiety/tension is in green. Columns are split by gene type, where protein-coding is in grey, rRNA is in purple, and tRNA is in light green.

Supplementary Figure 35. Pearson correlations of gene expression between 37 MT genes and 11 genes mapped from the general neuroticism locus and 33 mapped genes from anxiety/tension locus on chromosome 9 in 215 GTEx v8 cerebellar hemisphere tissue samples.

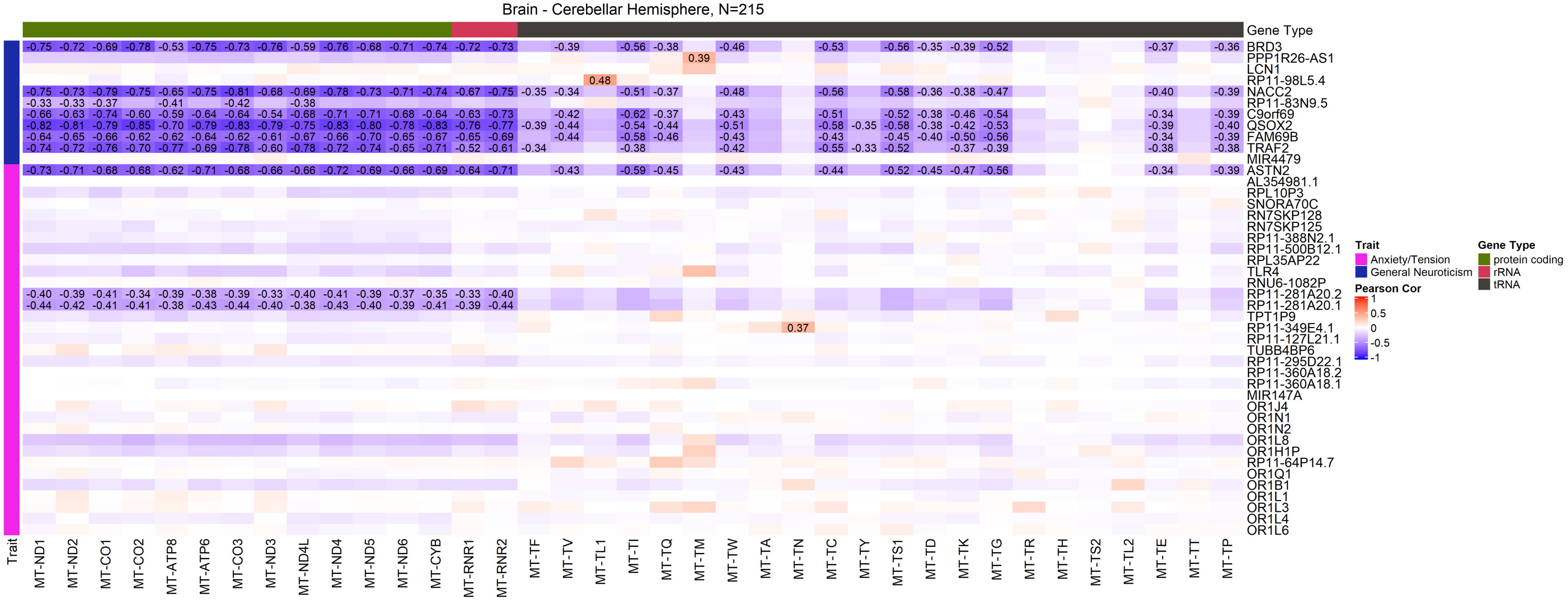

Colour in cells indicates the direction of a Pearson correlation and when labelled, indicate a statistically significant correlation at $P < 0.05/56{,}200$ (two-sided) where 56,200 is the total number of genes in GTEx v8. Rows are split by traits where general neuroticism is in dark blue and anxiety/tension is in green. Columns are split by gene type, where protein-coding is in grey, rRNA is in purple, and tRNA is in light green.

Supplementary Figure 36. Pearson correlations of gene expression between 37 MT genes and 11 genes mapped from the general neuroticism locus and 33 mapped genes from anxiety/tension locus on chromosome 9 in 209 GTEx v8 brain – frontal cortex tissue samples.

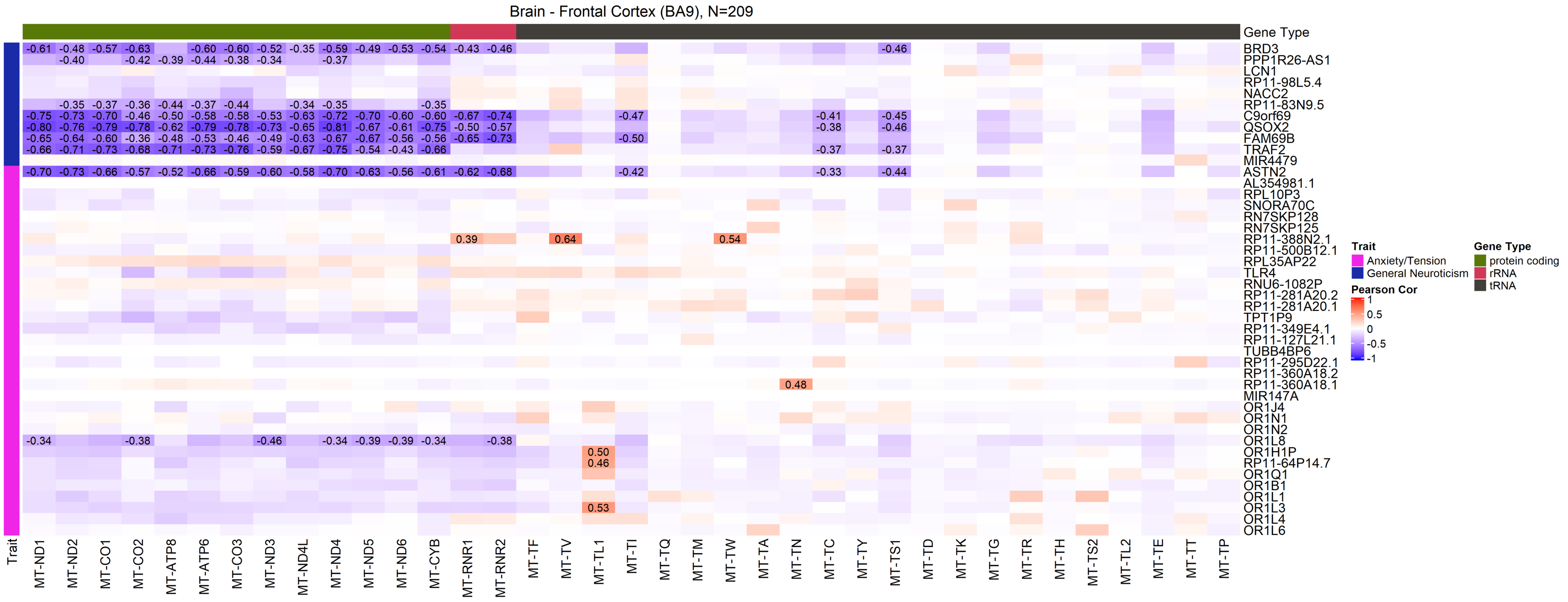

Colour in cells indicates the direction of a Pearson correlation and when labelled, indicate a statistically significant correlation at P < 0.05/56,200 (two-sided) where 56,200 is the total number of genes in GTEx v8. Rows are split by traits where general neuroticism is in dark blue and anxiety/tension is in green. Columns are split by gene type, where protein-coding is in grey, rRNA is in purple, and tRNA is in light green.

Supplementary Figure 37. Pearson correlations of gene expression between 37 MT genes and 11 genes mapped from the general neuroticism locus and 33 mapped genes from anxiety/tension locus on chromosome 9 in 197 GTEx v8 brain - hippocampus tissue samples.

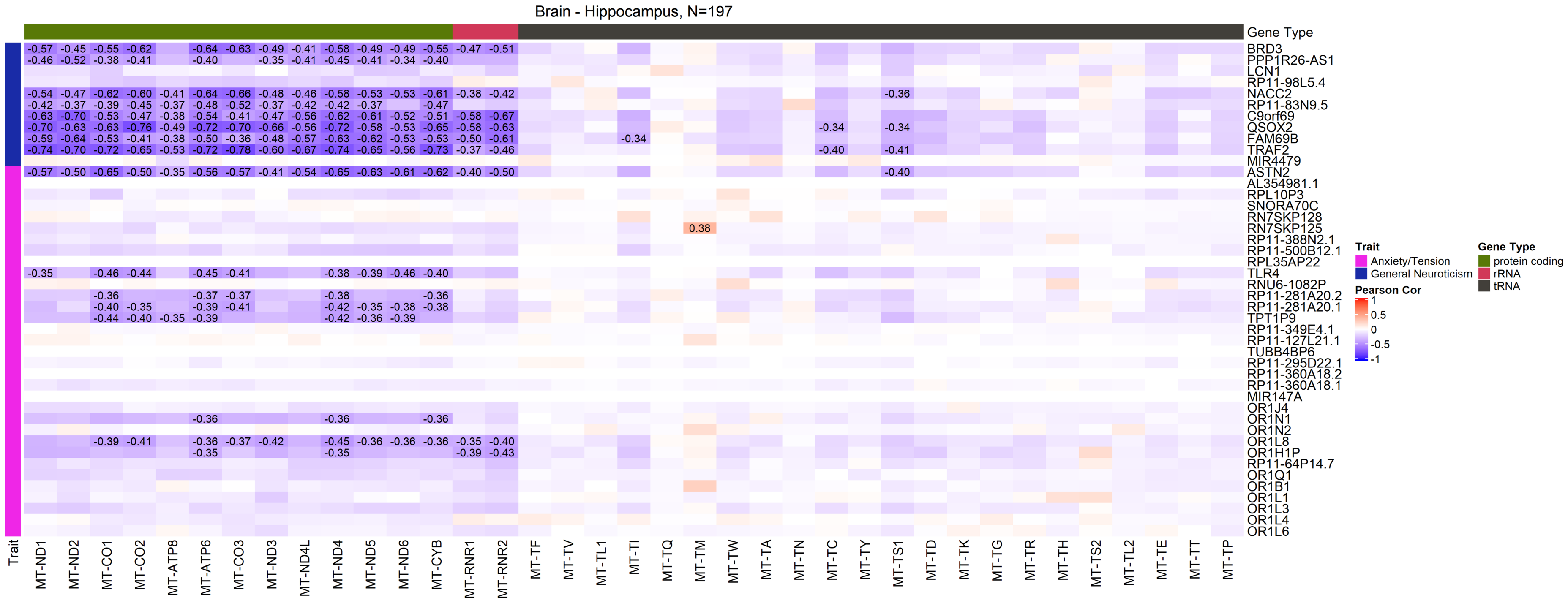

Colour in cells indicates the direction of a Pearson correlation and when labelled, indicate a statistically significant correlation at $P < 0.05/56{,}200$ (two-sided) where 56,200 is the total number of genes in GTEx v8. Rows are split by traits where general neuroticism is in dark blue and anxiety/tension is in green. Columns are split by gene type, where protein-coding is in grey, rRNA is in purple, and tRNA is in light green.

Supplementary Figure 38. Pearson correlations of gene expression between 37 MT genes and 11 genes mapped from the general neuroticism locus and 33 mapped genes from anxiety/tension locus on chromosome 9 in 202 GTEx v8 brain - hypothalamus tissue samples.

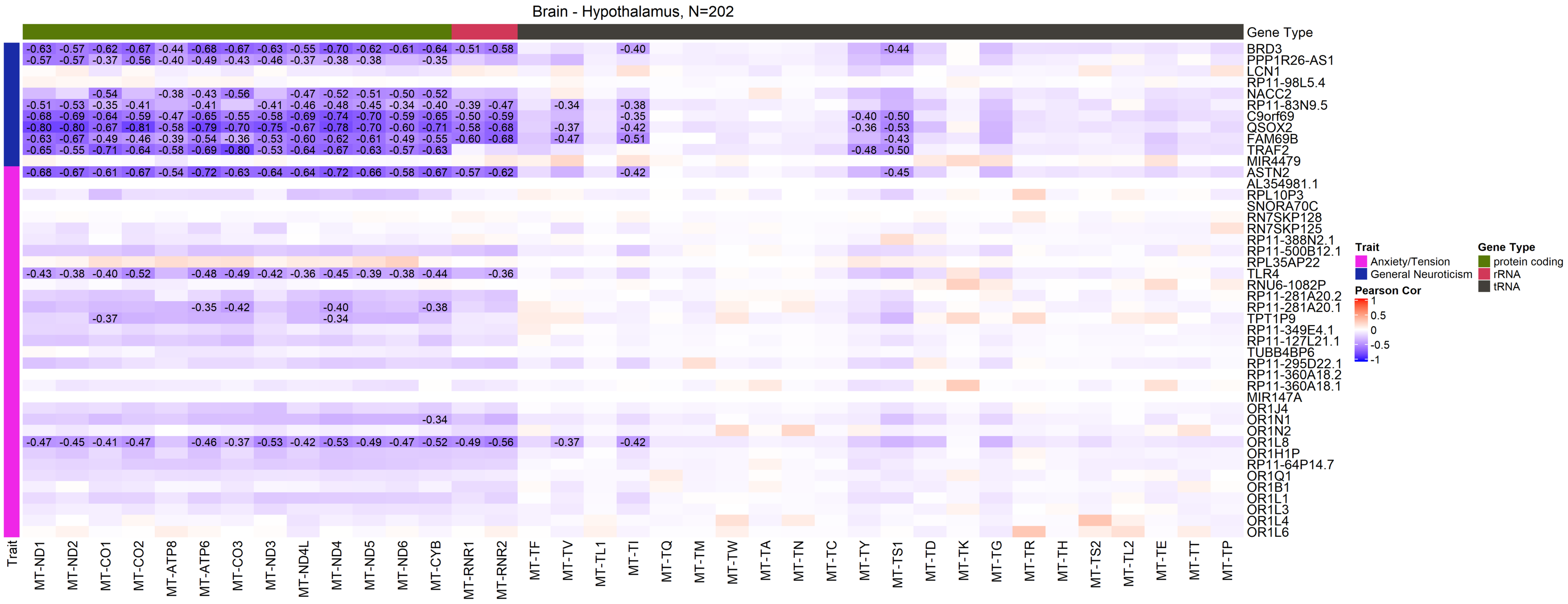

Colour in cells indicates the direction of a Pearson correlation and when labelled, indicate a statistically significant correlation at P < 0.05/56,200 (two-sided) where 56,200 is the total number of genes in GTEx v8. Rows are split by traits where general neuroticism is in dark blue and anxiety/tension is in green. Columns are split by gene type, where protein-coding is in grey, rRNA is in purple, and tRNA is in light green.

Supplementary Figure 39. Pearson correlations of gene expression between 37 MT genes and 11 genes mapped from the general neuroticism locus and 33 mapped genes from anxiety/tension locus on chromosome 9 in 246 GTEx v8 brain – nucleus accumbens tissue samples.

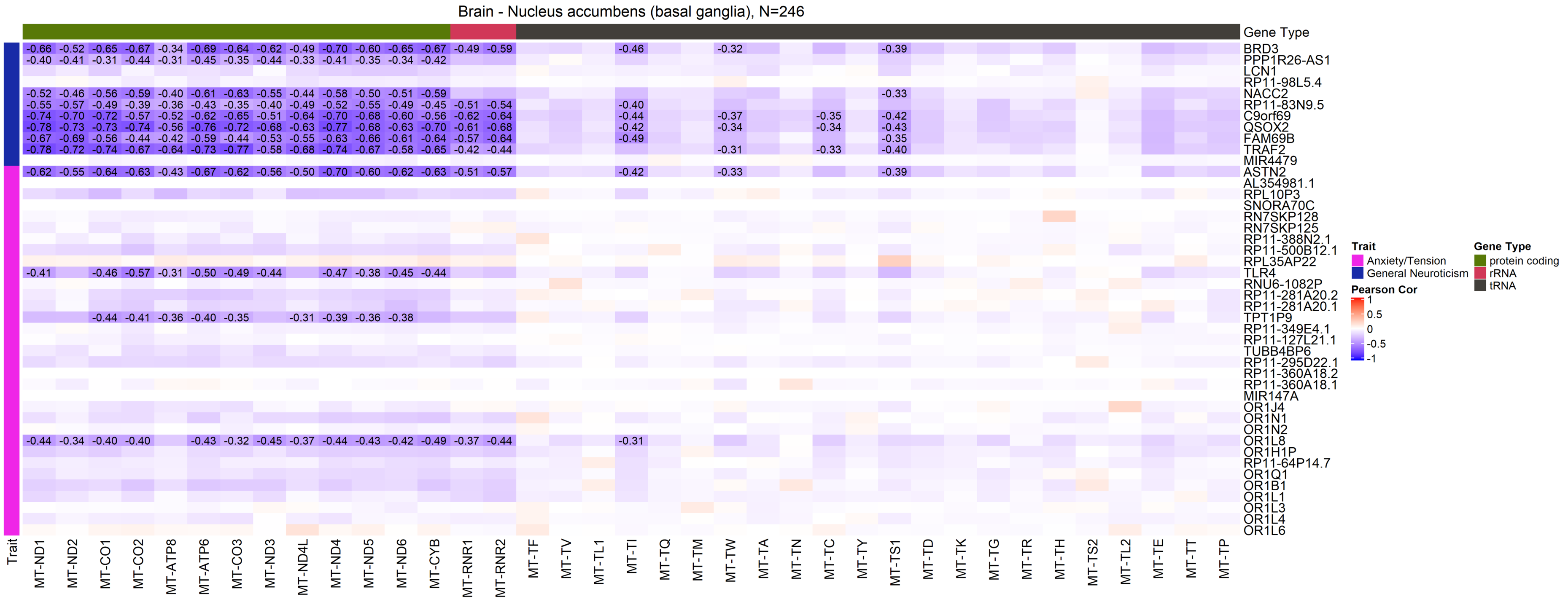

Colour in cells indicates the direction of a Pearson correlation and when labelled, indicate a statistically significant correlation at P < 0.05/56,200 (two-sided) where 56,200 is the total number of genes in GTEx v8. Rows are split by traits where general neuroticism is in dark blue and anxiety/tension is in green. Columns are split by gene type, where protein-coding is in grey, rRNA is in purple, and tRNA is in light green.

Supplementary Figure 40. Pearson correlations of gene expression between 37 MT genes and 11 genes mapped from the general neuroticism locus and 33 mapped genes from anxiety/tension locus on chromosome 9 in 205 GTEx v8 brain - putamen tissue samples.

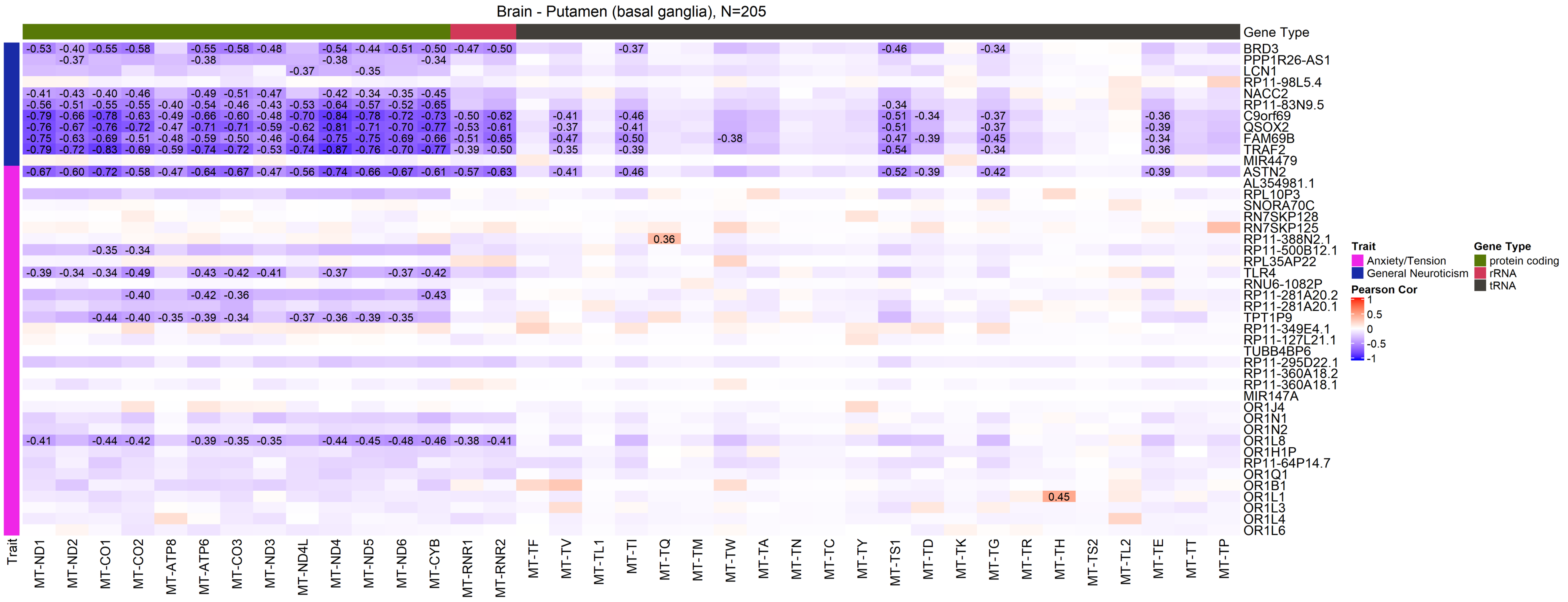

Colour in cells indicates the direction of a Pearson correlation and when labelled, indicate a statistically significant correlation at P < 0.05/56,200 (two-sided) where 56,200 is the total number of genes in GTEx v8. Rows are split by traits where general neuroticism is in dark blue and anxiety/tension is in green. Columns are split by gene type, where protein-coding is in grey, rRNA is in purple, and tRNA is in light green.

Supplementary Figure 41. Pearson correlations of gene expression between 37 MT genes and 11 genes mapped from the general neuroticism locus and 33 mapped genes from anxiety/tension locus on chromosome 9 in 139 GTEx v8 substantia nigra tissue samples.

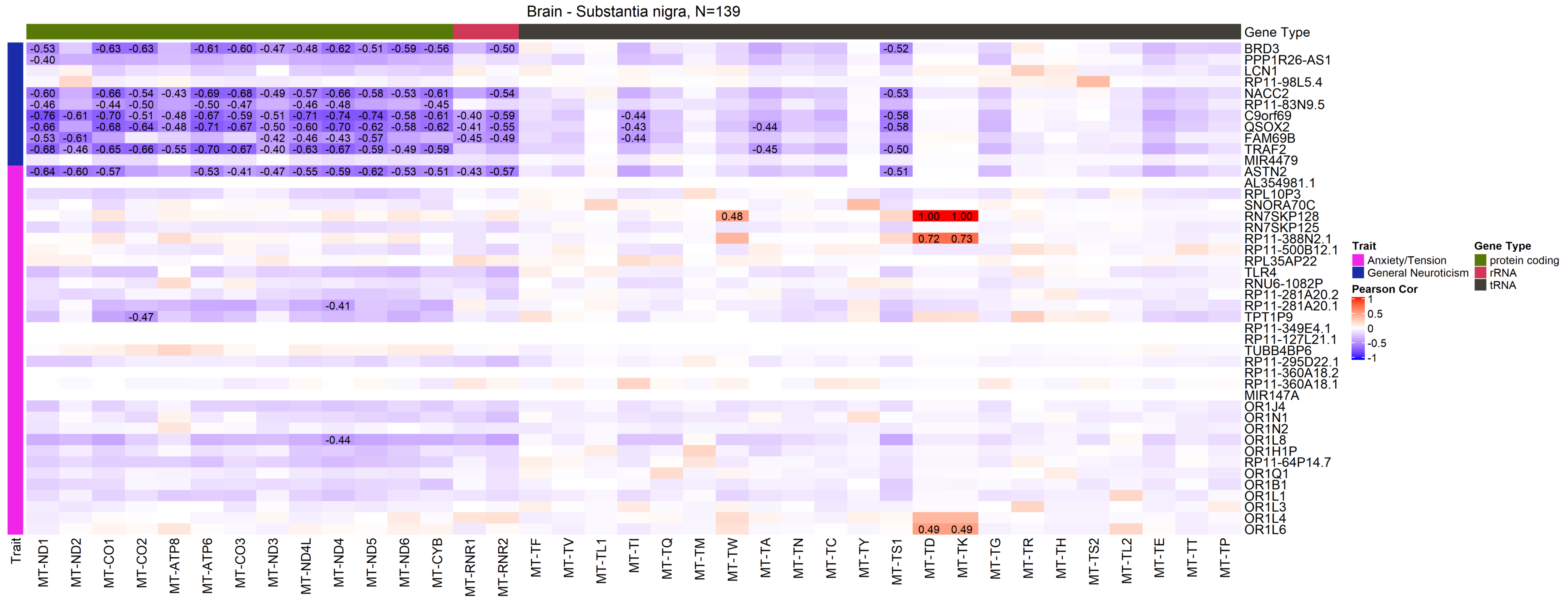

Colour in cells indicates the direction of a Pearson correlation and when labelled, indicate a statistically significant correlation at P < 0.05/56,200 (two-sided) where 56,200 is the total number of genes in GTEx v8. Rows are split by traits where general neuroticism is in dark blue and anxiety/tension is in green. Columns are split by gene type, where protein-coding is in grey, rRNA is in purple, and tRNA is in light green.

Supplementary Figure 42. STRING multiple protein search results.

a

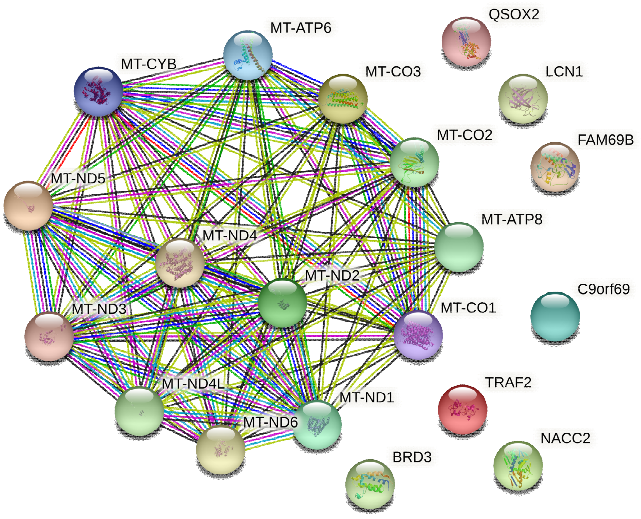

b

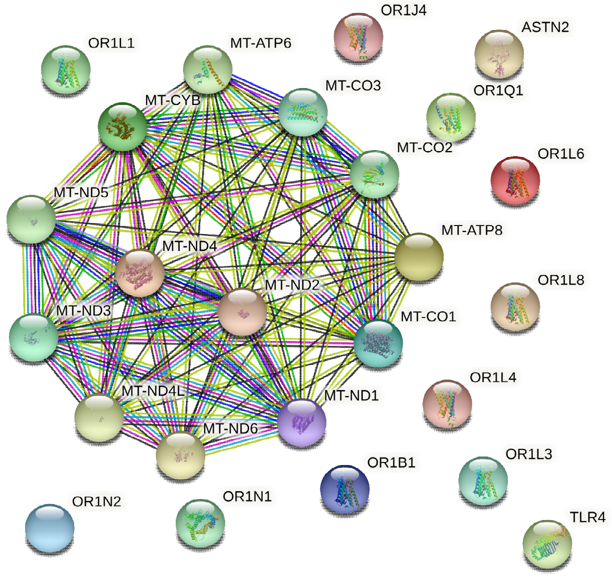

Figure shows multiple protein search results in STRING for genes mapped to the interaction locus on chromosome 9 for general neuroticism (**Panel a**) and anxiety/tension (**Panel b**). Whilst there are protein-protein interactions between the MT protein coding genes in both panels a and b, there are none between MT protein coding with nDNA protein coding genes on chromosome 9.

Supplementary Figure 43. Genes mapped to the anxiety/tension locus on chromosome 9 by FUMA.

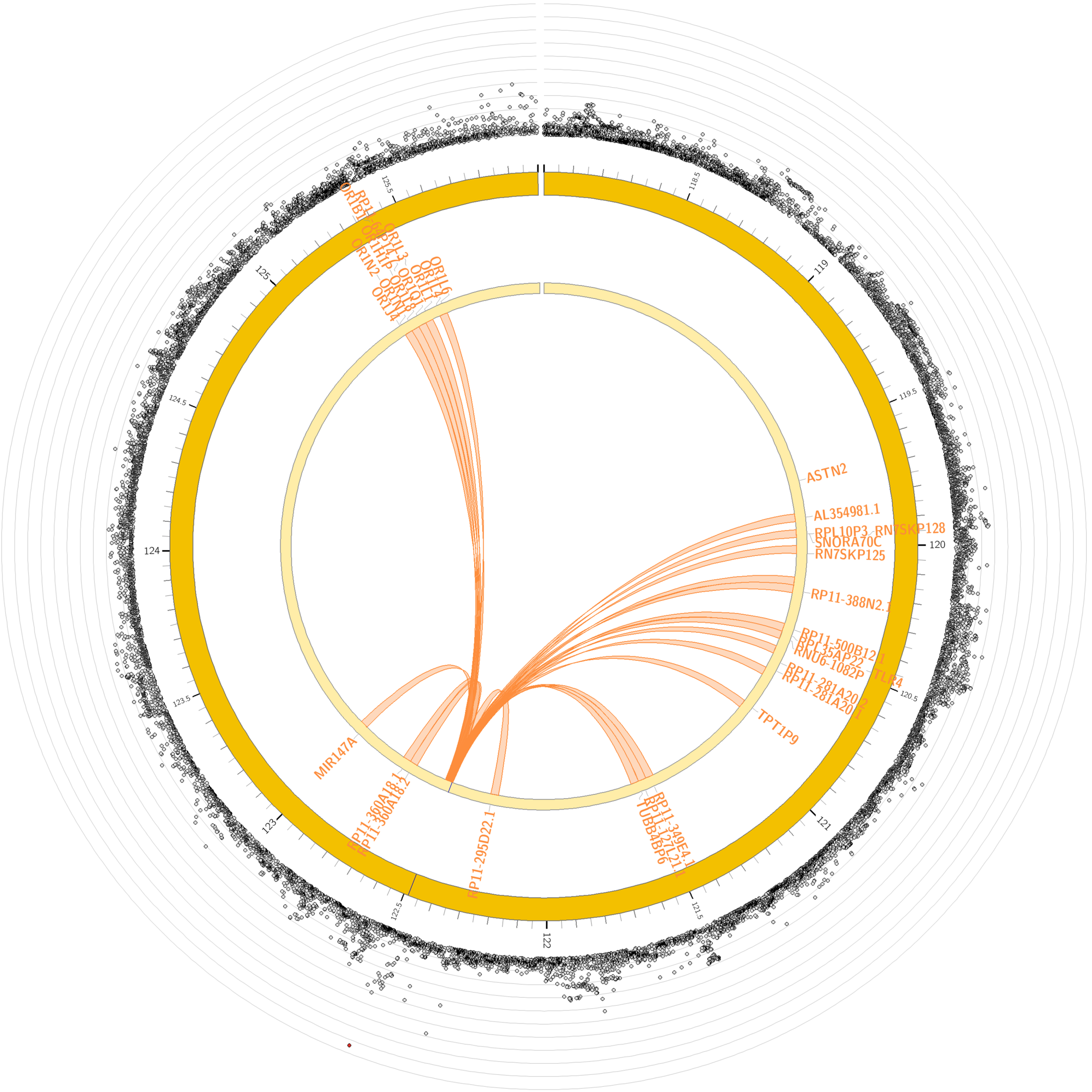

FUMA mapped 11 genes to general neuroticism locus on chromosome 9 due to chromatin interactions (orange), or eQTLs (green), or both (red). The outer layer shows –log10P (two-sided t-test) of Haplogroup-X-stratified nGWAS results. SNPs in LD with labelled lead SNP with r^2 >0.8, >0.6, >0.4 > 0.2 and ≤ 0.2 are coloured in red, orange, green, blue and grey respectively. The middle and inner layers are chromosome rings with and without coordinates, with the blue bar indicating the trait-associated genomic locus.

Supplementary Figure 44. Manhattan plot for H-haplogroup co-segregation GWAS.

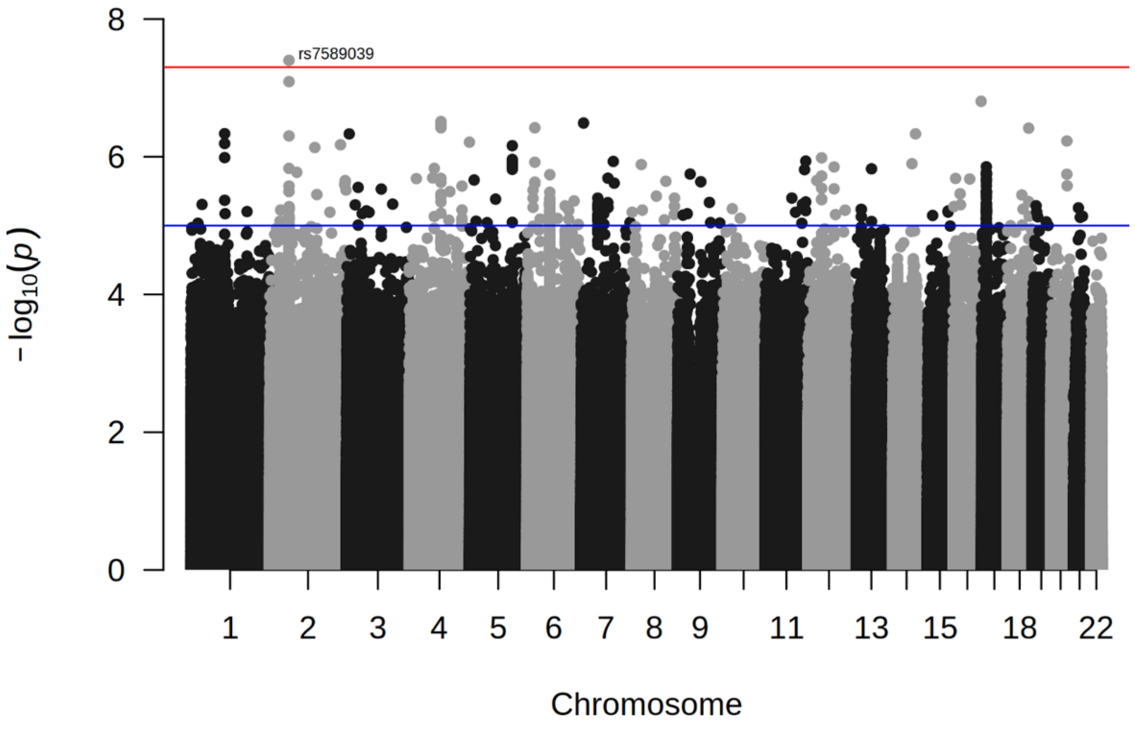

N=324,483 (including samples without the neuroticism phenotypes). Y-axis shows –log10 P value of the GWAS (two-sided t-test from PLINK linear regression) and X-axis shows the position of marker in the genome. Red line: genome-wide significant threshold of 5×10^{-8}. Blue line: suggestive significant threshold of 1×10^{-5}. The lead marker of each chromosome is labelled (if reached genome-wide significant level).

Supplementary Figure 45. Manhattan plot for HV-haplogroup co-segregation GWAS.

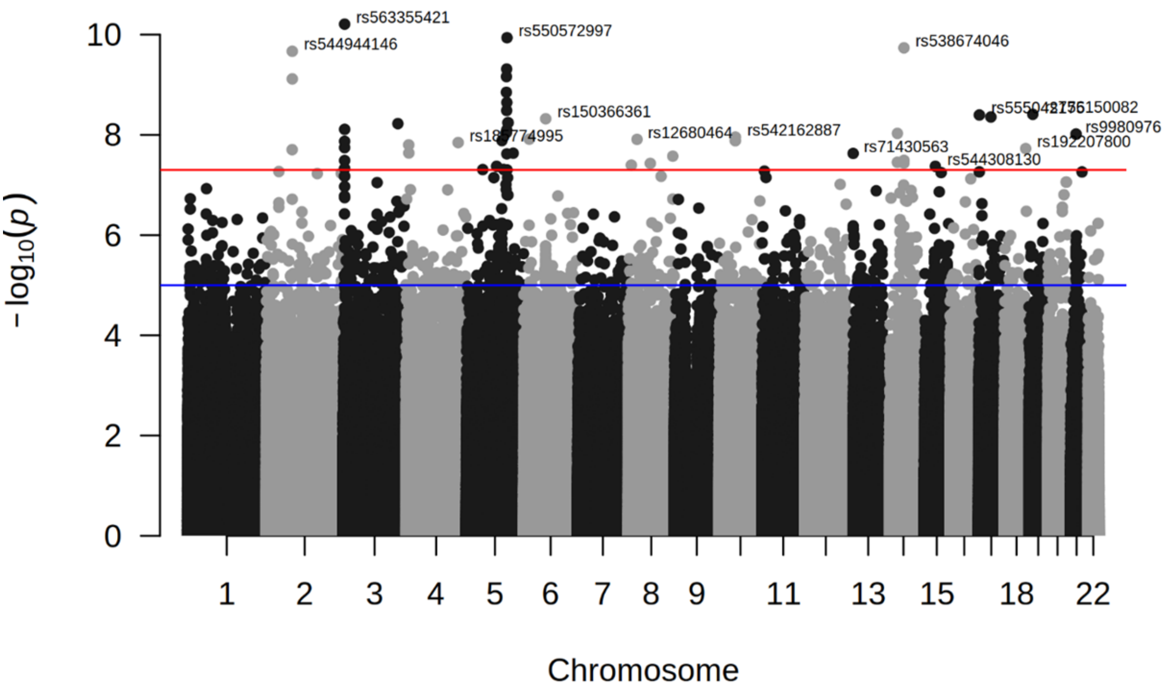

N=324,483 (including samples without neuroticism phenotypes that are not used in MT-GWAS). Y-axis shows –log10 P value of the GWAS (two-sided t-test from PLINK linear regression) and X-axis shows the position of marker in the genome. Red line: genome-wide significant threshold of 5×10^{-8}. Blue line: suggestive significant threshold of 1×10^{-5}. The lead marker of each chromosome is labelled (if reached genome-wide significant level).

Supplementary Figure 46. Manhattan plot for V-haplogroup co-segregation GWAS.

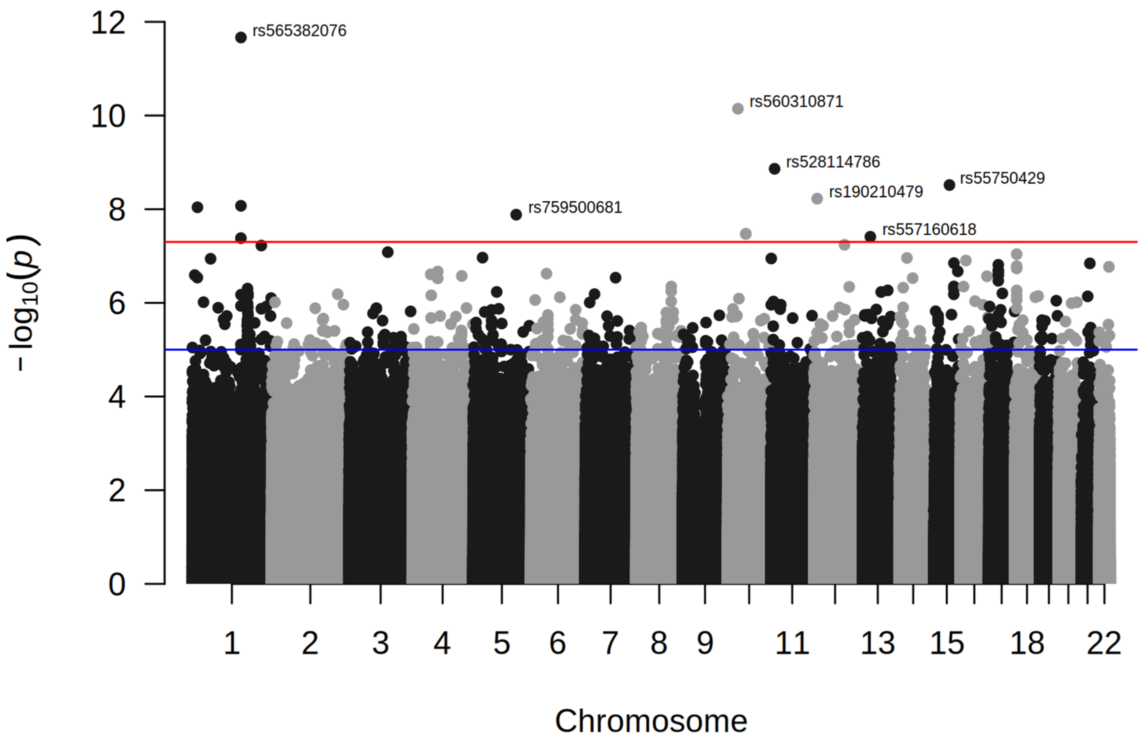

N=324,483 (including samples without neuroticism phenotypes that are not used in MT-GWAS). Y-axis shows –log10 P value of the GWAS (two-sided t-test from PLINK linear regression) and X-axis shows the position of marker in the genome. Red line: genome-wide significant threshold of 5×10^{-8}. Blue line: suggestive significant threshold of 1×10^{-5}. The lead marker of each chromosome is labelled (if reached genome-wide significant level).

Supplementary Figure 47. Manhattan plot for J-haplogroup co-segregation GWAS.

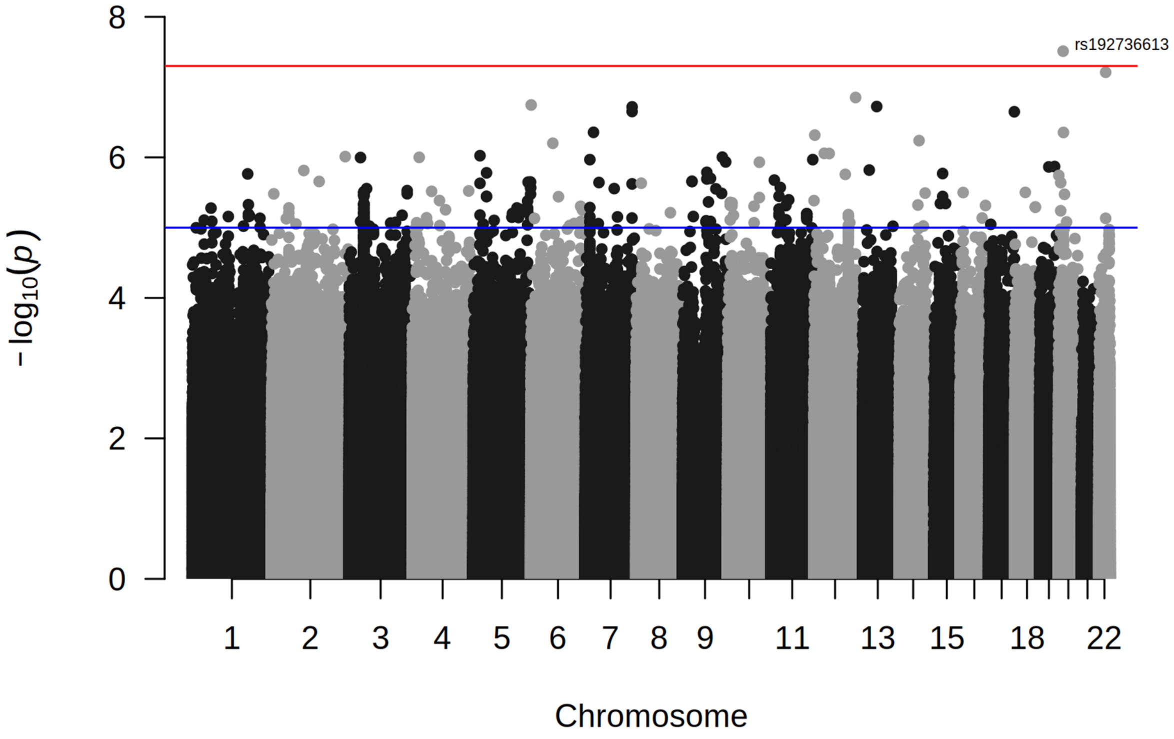

N=324,483 (including samples without neuroticism phenotypes that are not used in MT-GWAS). Y-axis shows –log10 P value of the GWAS (two-sided t-test from PLINK linear regression) and X-axis shows the position of marker in the genome. Red line: genome-wide significant threshold of 5×10^{-8}. Blue line: suggestive significant threshold of 1×10^{-5}. The lead marker of each chromosome is labelled (if reached genome-wide significant level).

Supplementary Figure 48. Manhattan plot for T-haplogroup co-segregation GWAS.

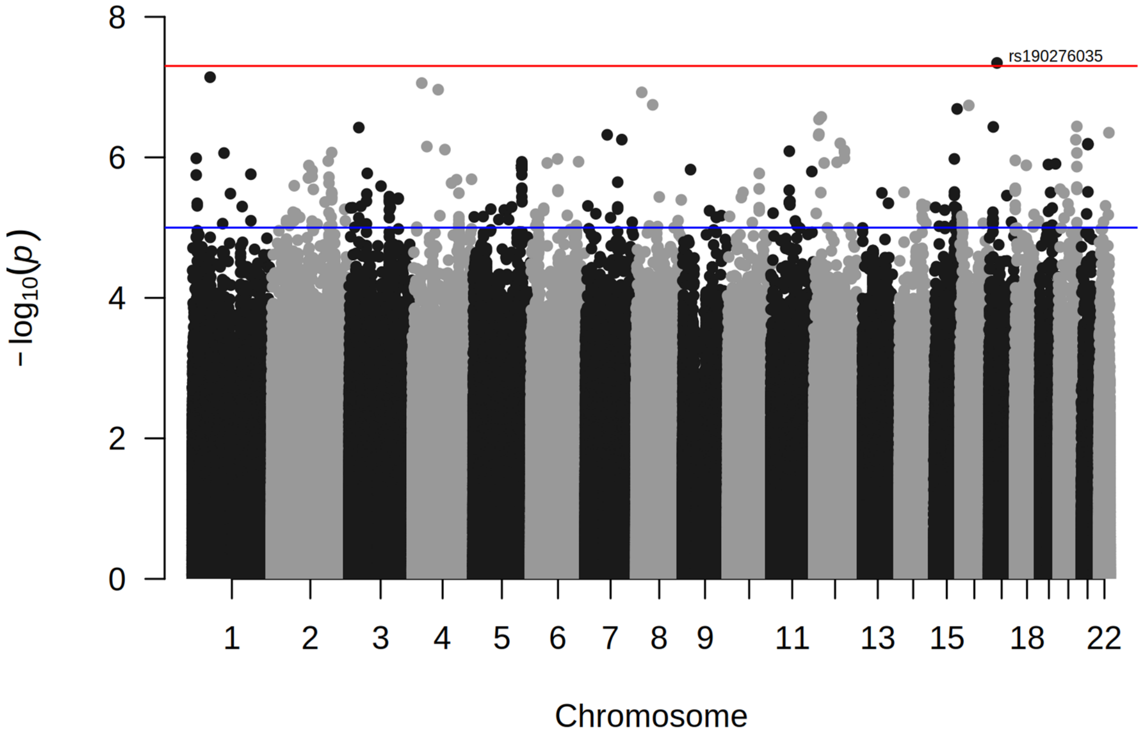

N=324,483 (including samples without neuroticism phenotypes that are not used in MT-GWAS). Y-axis shows –log10 P value of the GWAS (two-sided t-test from PLINK linear regression) and X-axis shows the position of marker in the genome. Red line: genome-wide significant threshold of 5×10^{-8}. Blue line: suggestive significant threshold of 1×10^{-5}. The lead marker of each chromosome is labelled (if reached genome-wide significant level).

Supplementary Figure 49. Manhattan plot for U-haplogroup co-segregation GWAS.

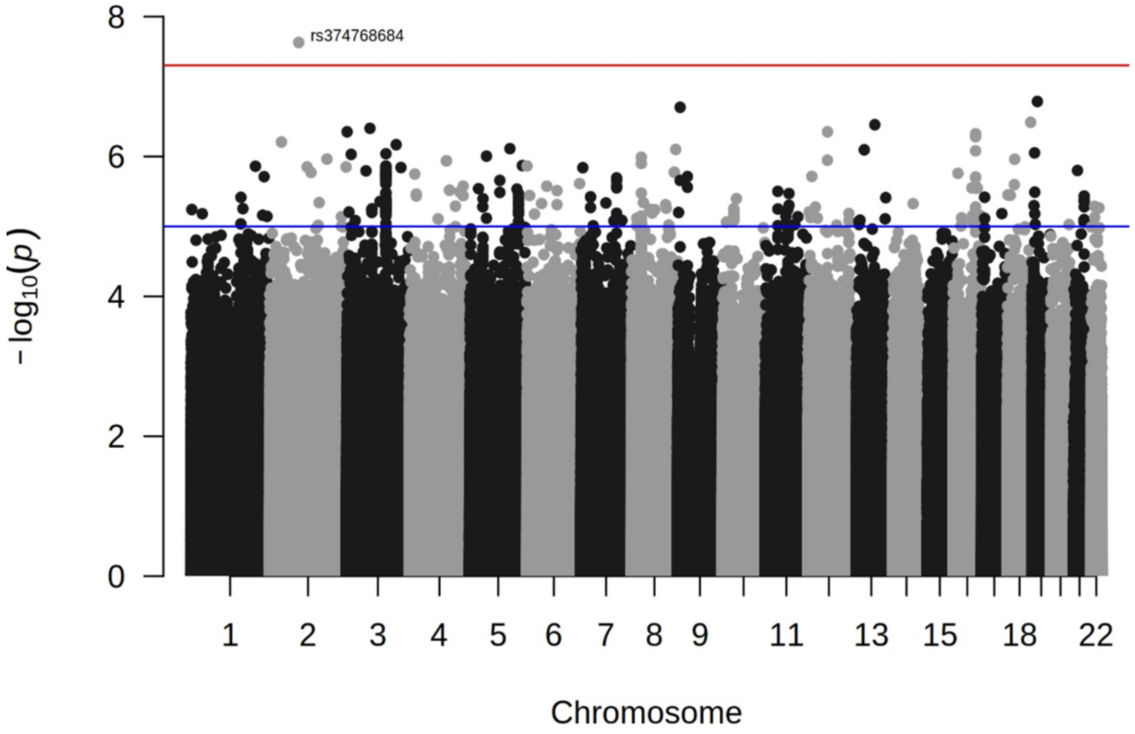

N=324,483 (including samples without neuroticism phenotypes that are not used in MT-GWAS). Y-axis shows –log10 P value of the GWAS (two-sided t-test from PLINK linear regression) and X-axis shows the position of marker in the genome. Red line: genome-wide significant threshold of 5×10^{-8}. Blue line: suggestive significant threshold of 1×10^{-5}. The lead marker of each chromosome is labelled (if reached genome-wide significant level).

Supplementary Figure 50. Manhattan plot for K-haplogroup co-segregation GWAS.

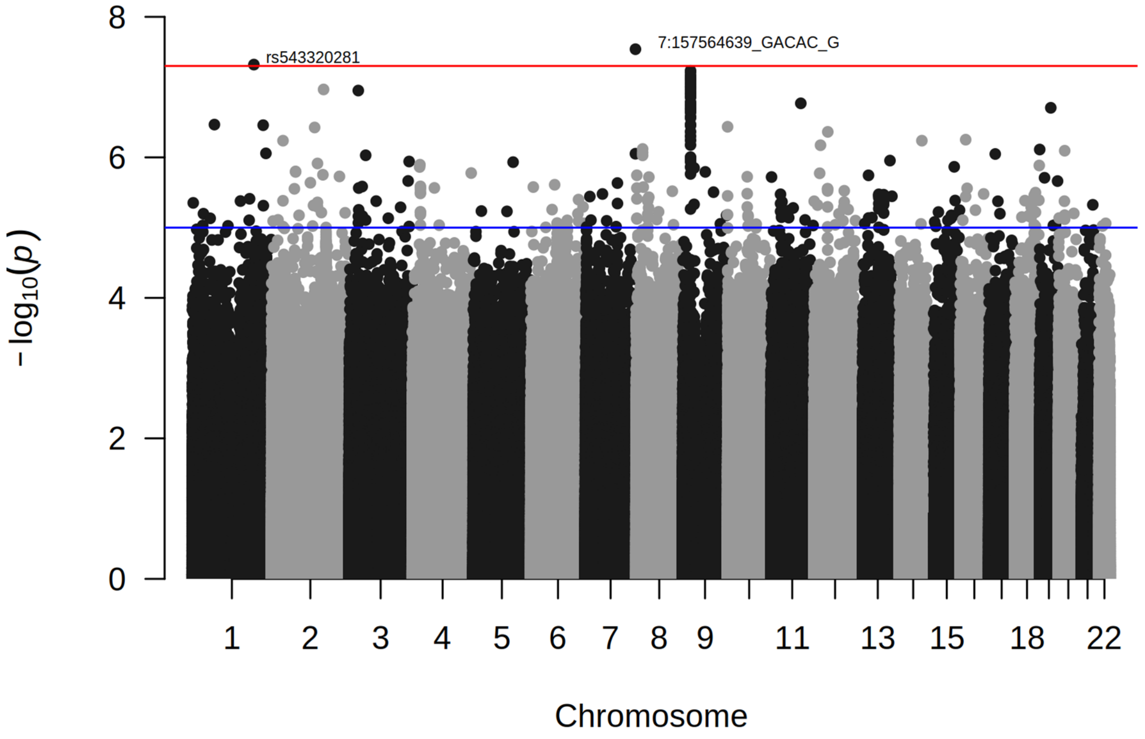

N=324,483 (including samples without neuroticism phenotypes that are not used in MT-GWAS). Y-axis shows –log10 P value of the GWAS (two-sided t-test from PLINK linear regression) and X-axis shows the position of marker in the genome. Red line: genome-wide significant threshold of 5×10^{-8}. Blue line: suggestive significant threshold of 1×10^{-5}. The lead marker of each chromosome is labelled (if reached genome-wide significant level).

Supplementary Figure 51. Manhattan plot for I-haplogroup co-segregation GWAS.

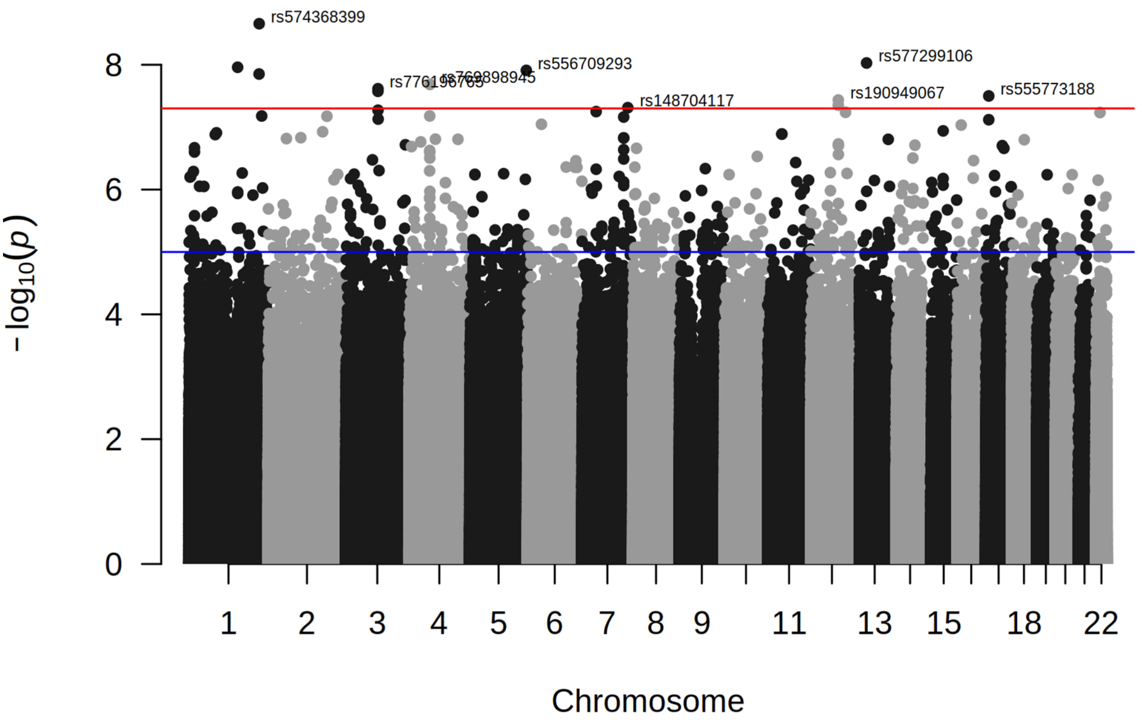

N=324,483 (including samples without neuroticism phenotypes that are not used in MT-GWAS). Y-axis shows –log10 P value of the GWAS (two-sided t-test from PLINK linear regression) and X-axis shows the position of marker in the genome. Red line: genome-wide significant threshold of 5×10^{-8}. Blue line: suggestive significant threshold of 1×10^{-5}. The lead marker of each chromosome is labelled (if reached genome-wide significant level).

Supplementary Figure 52. Manhattan plot for W-haplogroup co-segregation GWAS.

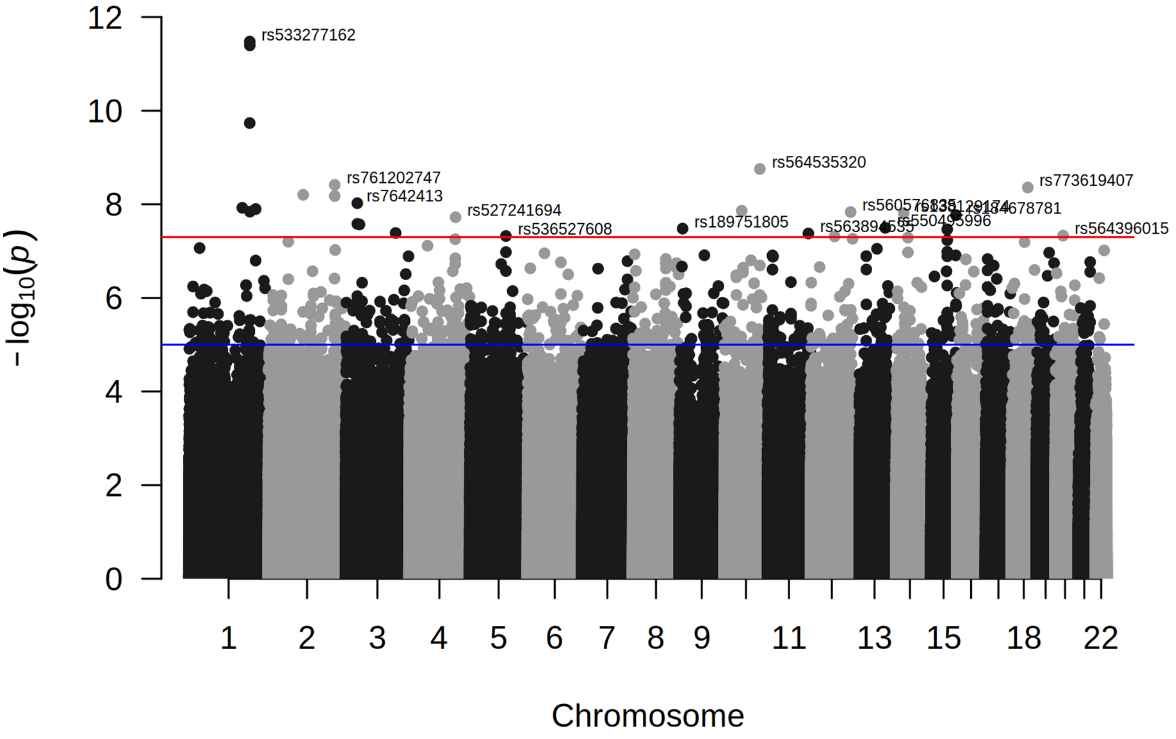

N=324,483 (including samples without neuroticism phenotypes that are not used in MT-GWAS). Y-axis shows –log10 P value of the GWAS (two-sided t-test from PLINK linear regression) and X-axis shows the position of marker in the genome. Red line: genome-wide significant threshold of 5×10^{-8}. Blue line: suggestive significant threshold of 1×10^{-5}. The lead marker of each chromosome is labelled (if reached genome-wide significant level).

Supplementary Figure 53. Manhattan plot for X-haplogroup co-segregation GWAS.

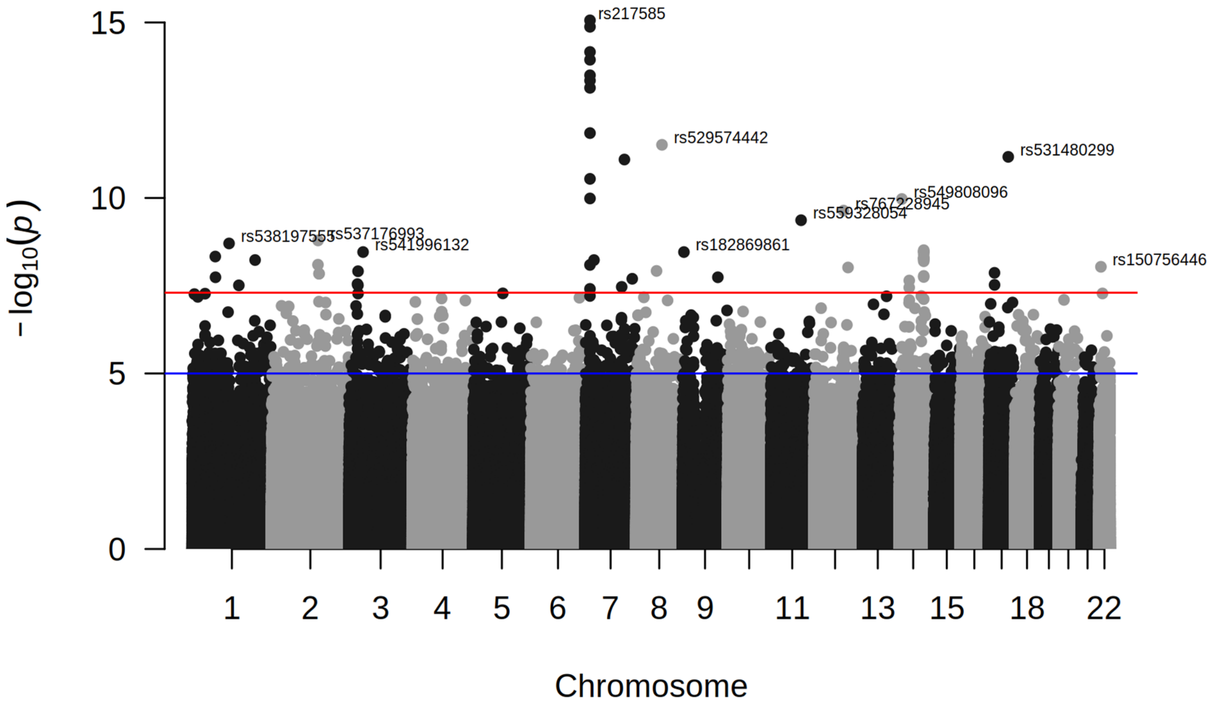

N=324,483 (including samples without neuroticism phenotypes that are not used in MT-GWAS). Y-axis shows –log10 P value of the GWAS (two-sided t-test from PLINK linear regression) and X-axis shows the position of marker in the genome. Red line: genome-wide significant threshold of 5×10^{-8}. Blue line: suggestive significant threshold of 1×10^{-5}. The lead marker of each chromosome is labelled (if reached genome-wide significant level).

Supplementary Figure 54. Manhattan plot for Super-**HV**-haplogroup co-segregation GWAS.

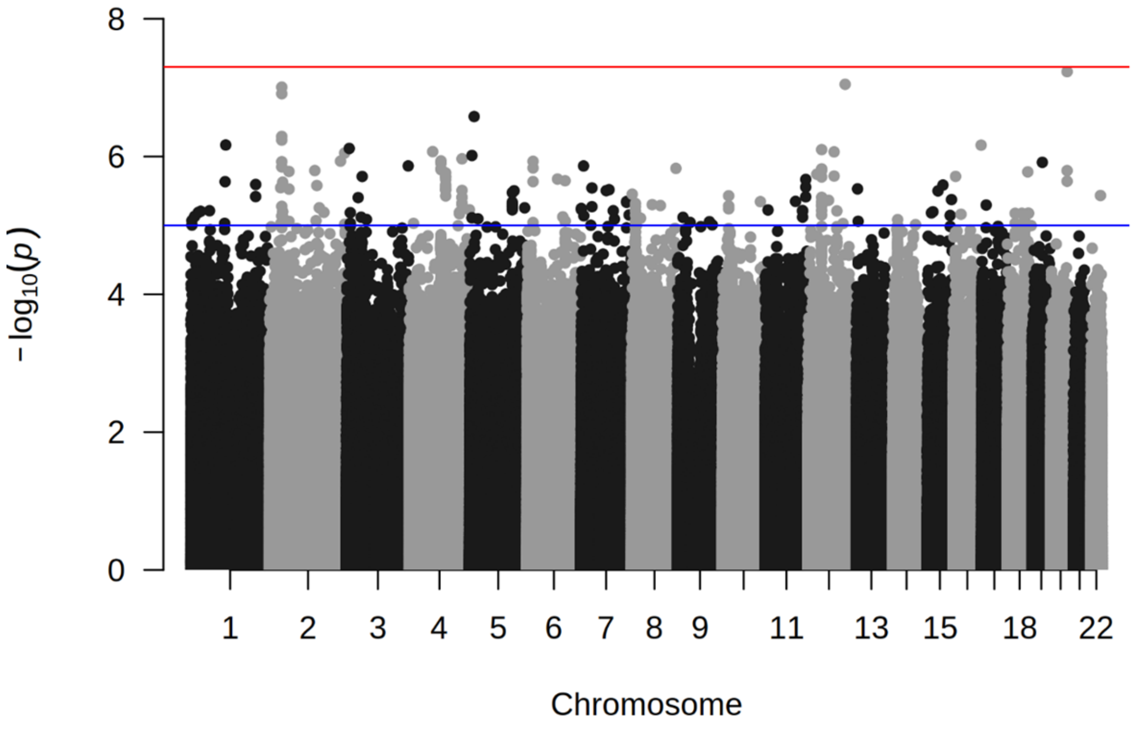

N=324,483 (including samples without neuroticism phenotypes that are not used in MT-GWAS). Y-axis shows –log10 P value of the GWAS (two-sided t-test from PLINK linear regression) and X-axis shows the position of marker in the genome. Red line: genome-wide significant threshold of 5×10^{-8}. Blue line: suggestive significant threshold of 1×10^{-5}. The lead marker of each chromosome is labelled (if reached genome-wide significant level).

Supplementary Figure 55. Manhattan plot for Super-**JT**-haplogroup co-segregation GWAS.

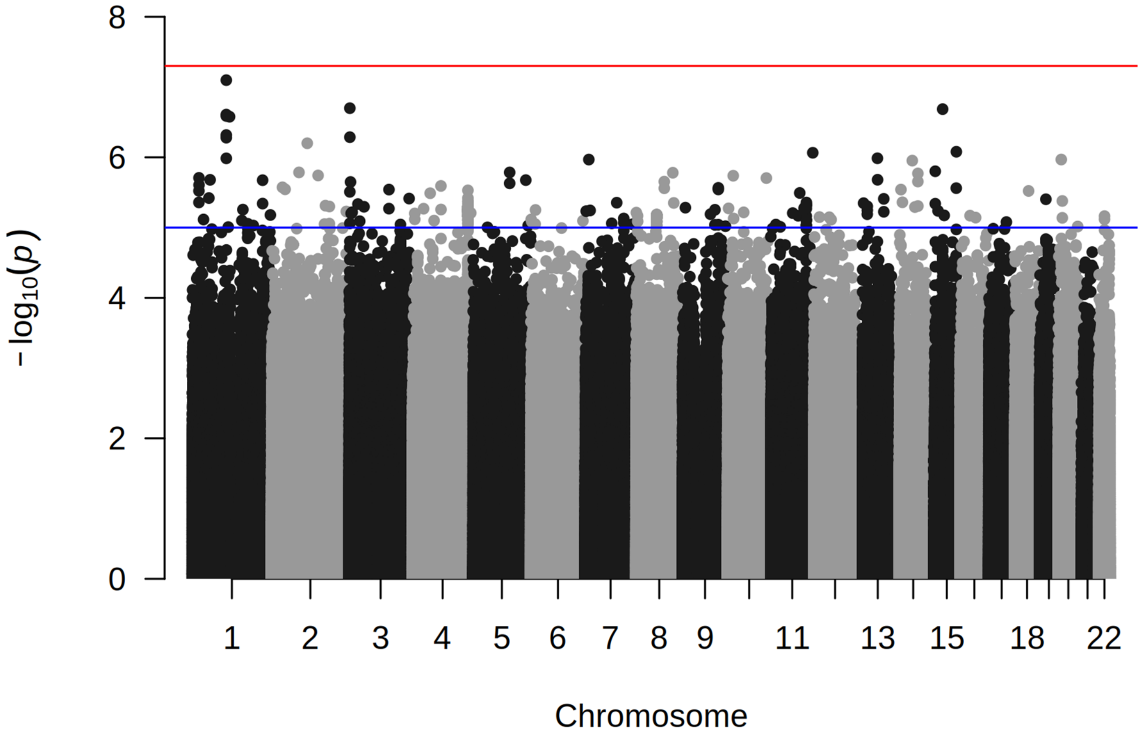

N=324,483 (including samples without neuroticism phenotypes that are not used in MT-GWAS). Y-axis shows –log10 P value of the GWAS (two-sided t-test from PLINK linear regression) and X-axis shows the position of marker in the genome. Red line: genome-wide significant threshold of 5×10^{-8}. Blue line: suggestive significant threshold of 1×10^{-5}. The lead marker of each chromosome is labelled (if reached genome-wide significant level).

Supplementary Figure 56. Manhattan plot for Super-**UK**-haplogroup co-segregation GWAS.

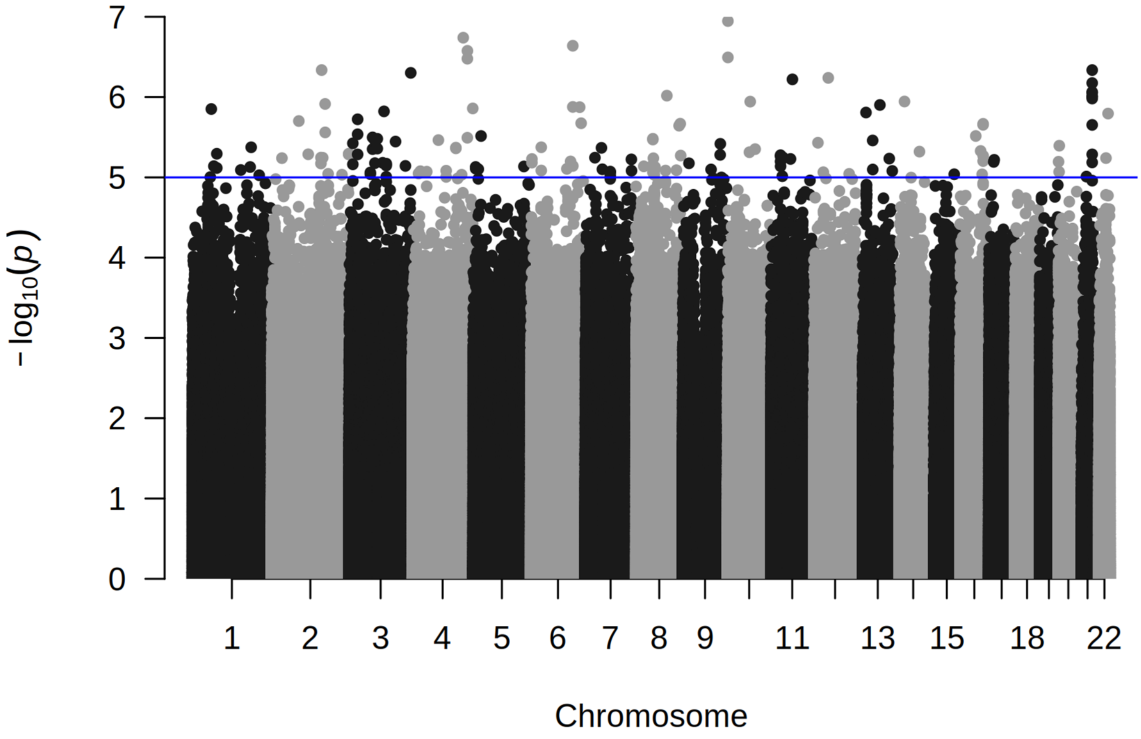

N=324,483 (including samples without neuroticism phenotypes that are not used in MT-GWAS). Y-axis shows –log10 P value of the GWAS (two-sided t-test from PLINK linear regression) and X-axis shows the position of marker in the genome. Red line: genome-wide significant threshold of 5×10^{-8}. Blue line: suggestive significant threshold of 1×10^{-5}. The lead marker of each chromosome is labelled (if reached genome-wide significant level).

Supplementary Notes

Co-segregation GWAS

To explore if co-segregation between MT-haplogroup and nuclear DNA was driving the association between haplogroup identity and the three neuroticism phenotypes, we performed nDNA GWAS of MT haplogroups. Here, we extracted the imputed data for our initial 332,047 unrelated white British samples and performed chromosome-wise quality control in PLINK2[2] by removing markers with INFO < 0.3, MAF < 0.0005, call rate < 0.95, Hardy-Weinberg equilibrium p value < 10^{-50} and removing samples with genotyping missingness > 0.1. Multi-allelic markers in the remaining data were also removed. No sample was removed due to missing neuroticism phenotypes. A total number of 19,651,021 markers and 332,042 samples passed QC.

At least one genome-wide significant co-segregation marker ($P < 5\times10^{-8}$) was found for each haplogroup (**Supplementary Figures 44-53**), indicating that MT-nDNA co-segregation can occur. However, no marker was found to co-segregate with the three super groups at $P < 5\times10^{-8}$ (**Supplementary Figures 54-56** and **Supplementary Data 25**). The most significant co-segregation signal is between rs217585 and X haplogroup ($P = 8.71\times10^{-16}$). The MAF of rs217585 is 0.0022 in X haplogroup in contrast to $5.16\times10^{-4} \pm 8.62\times10^{-5}$ in the other nine common haplogroups. We performed gene-set analysis and found evidence that the regions co-segregated with V and X haplogroups are enriched for MT-candidate genes (e.g. in V haplogroup, term: 'genes with evidence of mitochondrial localisation in any tissues in Mito Carta 3.0', $q = 0.017$; in X haplogroup, term 'known MT-nDNA genes in IMPI', $q = 0.003$) but not for other haplogroups (**Supplementary Data 26**).

Next, we extracted all markers that co-segregated with any haplogroups at $P < 1\times10^{-5}$ and cross-referenced their effect in nuclear GWAS of the three factors of neuroticism[7] to see whether our discoveries in MT-GWAS and MT-haplogroup analysis are likely driven by MT-nDNA co-segregation. For instance, in MT-haplogroup analysis we found that general neuroticism is associated with K haplogroup at $P = 8.19\times10^{-4}$. Among all nDNA markers associated with K haplogroup at $P < 1\times10^{-5}$, the one that associates with general neuroticism most significantly is rs34788318 with $P = 0.003$ (>

8.19×10^{-4}). Therefore, the association between general neuroticism and K haplogroup is unlikely driven by rs34788318 due to long distance correlation between this marker and K haplogroup. That is the case for all associations found in haplogroup analysis (**Supplementary Data 27**), except for the association between super **HV** and worry/vulnerability ($P = 2.03\times10^{-4}$ for super **HV** vs $P = 1.10\times10^{-4}$ for super **HV** co-segregated marker rs79025089). However, the observed correlation between super **HV** and rs79025089 ($r = -0.0088 \pm 0.0018$, $P = 5.22\times10^{-7}$) has an effect size too small to explain the association between super **HV** and worry/vulnerability. Therefore, we can confirm that none of the observed haplogroup-association results identified were due to confounding of single MT-nDNA co-segregating markers.

Finally, we looked up the co-segregation P values of our MT-nDNA interaction markers in the corresponding GWAS[7]. There was no evidence that the two markers showing evidence of interaction were co-segregation markers (rs72771986 with $P = 0.48$ in X co-segregation GWAS for the general factor of neuroticism, and rs181210427 with $P = 0.15$ in T co-segregation GWAS of anxiety/tension). Thus, our previous MT-nDNA interaction discoveries are also not a result of MT-nDNA co-segregation.

Supplementary Method

MT Genotype Data QC

Genotype QC was performed in PLINKv1.90b6.20 for each subset individually.

We first extracted UKBB and UKBL participants from the data.

#input, raw plink files, without file extensions

#array.id, individuals genotyped using the array

#array.mt.snps, a list of SNPs on the array

#array.mt.snps_failed, a list of SNPs on the array failed batch QC

#output, output file name

```
plink \
--bfile $input \
```

```
--keep $array.id \

--extract ../$array.mt.snps \

--exclude ../$array.mt.snps_failed \

--make-bed \

--out $output
```

We then removed low quality markers (primary QC).

#input, input plink file from previous step

```
geno1=0.2

maf1=0.0001

plink --bfile $input \

--geno $geno1 \

--maf $maf1 \

--make-bed \

--out "qc1_geno$geno1""_maf$maf1"
```

Subsequently, we performed secondary QCs to remove markers with MAF < 0.0001 and call rate < 0.98 and to remove individuals with genotype rate < 0.95. QC will stop till no marker/individual is removed.

```
geno2=0.02

maf2=0.0001

mind2=0.05



run=2

geno1=0.2

maf1=0.0001

plink \

--bfile "qc1_geno$geno1""_maf$maf1" \

--geno $geno2 \
```

```
--maf $maf2 \

--make-bed \

--mind $mind2 \

--out "qc$run""_mind$mind2""_geno$geno2""_maf$maf2"

lines1=`wc -l "qc1_geno$geno1""_maf$maf1.bim" | cut -f1 -d' '`

flines1=`wc -l "qc1_geno$geno1""_maf$maf1.fam" | cut -f1 -d' '`

lines2=`wc -l "qc$run""_mind$mind2""_geno$geno2""_maf$maf2.bim" | cut -f1 -d' '`

flines2=`wc -l "qc$run""_mind$mind2""_geno$geno2""_maf$maf2.fam" | cut -f1 -d' '`


while [ "$lines1" -ne "$lines2" ] || [ "$flines1" -ne "$flines2" ]

do

lines1=`wc -l "qc$run""_mind$mind2""_geno$geno2""_maf$maf2.bim" | cut -f1 -d' '`

flines1=`wc -l "qc$run""_mind$mind2""_geno$geno2""_maf$maf2.fam" | cut -f1 -d' '`

run2=`expr $run + 1`

../../mtDNA/plink \

--bfile "qc$run""_mind$mind2""_geno$geno2""_maf$maf2" \

--geno $geno2 \

--maf $maf2 \

--make-bed \

--mind $mind2 \

--out "qc$run2""_mind$mind2""_geno$geno2""_maf$maf2"

lines2=`wc -l "qc$run2""_mind$mind2""_geno$geno2""_maf$maf2.bim" | cut -f1 -d' '`

flines2=`wc -l "qc$run2""_mind$mind2""_geno$geno2""_maf$maf2.fam" | cut -f1 -d' '`

run=$run2

done
```

MT Genotype “Pre-phasing” and Imputation

We prepared data to IMPUTE 2 readable format using SHAPEIT 2.

#inputefile, QCed genotype plink file without extension

#outputfile, output file name

```
shapeit --input-bed $inputfile.bed $inputfile.bim $inputfile.fam \

--thread 16 \

--duohmm \

--rho 4.0E-12 \

-O $outputfile.phased \

--output-log $outputfile.phased
```

Imputation was conducted at sub-cohort level in the window size of 16,579 kb (length of mtDNA) using MT-1000G as reference.

#kentmap.map, oxg1000ref.gen and fin.oxg1000ref.legend are mtDNA reference panel files

#inputfile, shapeit.phased.haps from previous step

#outputfile, output file name

```
impute2 -m kentmap.map \

-h oxg1000ref.gen \

-l fin.oxg1000ref.legend  \

-known_haps_g $inputfile.phased.haps \

-int 1 16579 \

-Ne 20000 \

-o $outputfile.imputed
```

Hard calls were made from genotype probabilities using PLINKv1.90b6.20.

#inputfile1, impute2.imputed file from previous step

#imputfile2, shapeit2.sample file from two steps before

#outputfile, output file name

```
plink \

--gen $inputfile1.imputed \
```

```
--sample $inputefile2.phased.sample \

--hard-call-threshold 0.1 \

--oxford-single-chr 26 \

--make-bed \

--out $outputfile
```

MT Imputation QC

We set heterozygosity to NA in plink.

#inputfile, binary plink files from previous step

#outputfile, output file name

```
plink --bfile $inputfile \

--sex-mixed-mt-missing \

--make-bed \

--out $outputfile
```

To QC the imputation data, we first extract markers with info > 0.3. The list of markers was prepared in R.

#R script

#inputfile1, impute2.imputed_info from imputation step

```
s1 <- read.table(paste0(inputfile1,”.imputed_info"),header=T)

markers1 <- s1$rs_id[which(s1$info > 0.3)]

write.table(markers1,"info0.3",quote=F,row.names=F,col.names=F)
```

Then we extracted these markers from the imputation data in PLINK.

#inputfile, het_removed plink binary file from 2 steps before.

#markerlist, markers with info > 0.3 from previous step.

#outputfile, output file name

```
plink \

--bfile $inputfile \
```

```
--extract $markerlist \

--make-bed \

--out $outputfile
```

Finally, we performed quality control on the imputed data using the same script used in the secondary MT genotype data QC to remove markers with MAF < 0.01 and call rate < 0.95 and individuals with genotype rate < 0.9.

MT GWAS

GWAS conducted in each subset individually and then meta-analysed in PLINKv1.90b6.20.

#inputfile, ukbb or ukbl qced imputed data

#phe, phenotype (residuals)

#outputfile, name of the output file

```
plink --bfile $inputfile \

--pheno $phe \

--linear \

--maf 0.01 \

--ci 0.95 \

--out $outputfile
```

#inputfile1-2, ukbb and ukbl GWAS results

#outputfile, output file name

```
plink --meta-analysis $inputfile1 $inputfile2 + qt study report-all \

--meta-analysis-a1-field A1 \

--meta-analysis-bp-field BP \

--meta-analysis-chr-field CHR \

--meta-analysis-ess-field NMISS \

--meta-analysis-p-field P \
```

```
--meta-analysis-se-field SE \

--meta-analysis-snp-field SNP \

--out $outputfile
```

MT Haplogroup Derivation

We first transformed plink binary files to ped files using PLINKv1.90b6.20 and then transformed ped files to hsd format in R v3.6.1.

#inputfile, plink bed files without extension

#outputfile, output file name

```
plink –-bfile $inputfile --recode --out $outputfile
```

#R script

#input.file, name of ped file without extension

```
df <- read.table(paste0(input.file,".ped"),header=F)

map <- read.table(paste0(input.file,".map"),header=F)

id <- df[,1]

df <- df[,-c(1:6)]

df <- df[,2*c(1:(ncol(df)/2))]

cl3 <- "?"

output.file <- file(paste0(input.file,".hsd"),"w")

writeLines(paste(c("ID","Range","Haplogroup","Polymorphisms"), collapse = "\t"),

 con=output.file)

for (i in 1:nrow(df)){

         temp <- which(df[i,] !=0)

         cl1 <- id[i]

         cl2 <- paste(map$V4[temp], collapse = ';')

         cl4 <- paste(paste0(as.character(map$V4)[temp], unlist(df[i,temp])),coll
apse='\t')
```

```
        writeLines(paste(c(cl1,cl2,cl3,cl4), collapse = "\t"), con=output.file)

}

close(output.file)
```

Subsequently, we loaded the hsd file into haplogrep for haplogroup identification.

#inputfile, hsd file

#outputfile, name of the output file

```
haplogrep classify --in input.file --format hsd –out output.file
```

Afterwards, we set haplogroup to NA if the quality score was lower than 0.9 and we clustered haplogroups into macro haplogroup and super haplogroup in R v3.6.1.

#inputfile, haplogrep output file, cut 1st, 2nd, 4th columns if needed

#outputfile, output file name

#R script

```
df <- read.table(inputfile,header=T)

df$Haplogroup[which(df$Quality <= 0.9)] <- NA

df <- df[complete.cases(df),]

library(stringr)

hap <- unique(as.character(df$Haplogroup))

up <- strsplit(hap,"[^::A-Z::]")

macro <- c()

for (i in 1:length(hap)){

  macro[i] <- unlist(up[[i]])[1]

}

super <- array("Other",length(hap))

super[which(macro == "H" | macro == "V" | macro == "HV")] <- "HV"

super[which(macro == "U" | macro == "K")] <- "UK"

super[which(macro == "J" | macro == "T")] <- "JT"
```

```
df <- cbind(df,macro,super)

write.table(df, outputfile, quote=F, row.names=F, col.names=T)
```

MT Haplogroup Association Analysis

Haplogroup association analysis was performed in R v3.6.1 using customised script.

#R script

#hg, haplogroup file, including columns, id, macro, super

#pheno, phenotypic residuals file, including columns id, phe

#outputfile, name your output file

```
df <- read.table(hg, header=T)

phe <- read.table(pheno,header=T)

df.lm <- data.frame(phe=as.numeric(phe$phe),hg=as.factor(df[match(phe$id,df$id),
"macro"]))

##change macro to super for super haplogroup analysis

df.lm <- df.lm[complete.cases(df.lm),]

temp.count <-
t(t(sort(summary(as.factor(df.lm$hg),length(unique(df.lm$hg))),decreasing=T)))

lvl <- c(rownames(temp.count)[which(temp.count >=nrow(df.lm)*0.01)],"Other")

hg.trans <- cbind(rownames(temp.count),row.names(temp.count))

hg.trans[which(temp.count < nrow(df.lm)*0.01),2] <- "Other"

temp.hg <- df.lm$hg

temp.hg <- hg.trans[match(df.lm$hg,hg.trans[,1]),2]

temp.count <-
t(t(sort(summary(as.factor(temp.hg),length(unique(temp.g))),decreasing=T)))

fit.model1 <- lm(df.lm$phe ~ factor(temp.hg,levels=lvl))

temp.out1 <- summary(fit.model1)$coefficients[2:length(lvl),]

rownames(temp.out1) <- lvl[2:length(lvl)]

temp.out2 <- c()

for (j in 1:(length(sup.f1)-1)){
```

```
temp.hg <- df.lm$hg

temp.hg[which(df.lm$hg != lvl[j])] <- 0

temp.hg[which(df.lm$hg == lvl[j])] <- 1

fit.model2 <- lm(df.lm$phe ~ as.factor(temp.hg))

temp.out2 <- rbind(temp.out2,summary(fit.model2)$coefficients[2,])

}

rownames(temp.out2) <- lvl[1:(length(lvl)-1)]

sum.out <- cbind(temp.count,

temp.out1[match(rownames(temp.count),rownames(temp.out1)),],

temp.out2[match(rownames(temp.count),rownames(temp.out2)),])

colnames(sum.out) <-

c("HG.Count",paste0(rep(c("Model1","Model2"),each=4),

rep(c(".Est",".SE",".T",".P"), 2)))

write.csv(sum.out, outputfile ,quote=F,row.names=T)
```

Simulations

Simulations were performed to investigate the number of independent associations and the associated unit (MT SNP or haplogroup) that most likely driven the signal where the detected haplogroup and SNP associated with the same trait are incompletely correlated with each other (i.e. not haplogroup defining marker). Each simulated phenotype was composed of an environmental effect and a genetic effect (either a SNP or a haplogroup). The assigned genetic effect was the same as the observed effect for the MT associated unit in real data. Afterwards, an uncorrelated environmental effect with specified mean and variance was simulated, accounting for all unmeasured factors. The final simulated phenotype was calculated as the sum of the two above, which has mean of 0 and variance of 1. When the genetic effect was assigned to a SNP, linear regression was performed to estimate the association effect of the alternative associated unit (i.e. haplogroup), and vice versa. 1000 replicas were conducted for each case to generate an empirical distribution of the effect size where the 'true' causal effect was given to the alternative associated unit. Finally, an empirical p value was computed by comparing the observed effect estimate to its quantile in the distribution. If the observed effect of a MT associated unit was not significantly different from the distribution when the 'true' causal effect was assigned to the alternative associated unit at $P < 0.05$, it is considered that the former signal was contributed by the later due to their correlation. Simulations were conducted in R v3.6.1[1] using the same UKBB + UKBL post-QC

imputed markers used for MT-GWAS and the derived haplogroups used for MT-haplogroup association analysis. The script used to perform the simulations is presented below.

#R script

#hg, haplogroup, its observed effect in real data is eff_hg

#SNP, mt SNP, its observed effect in real data is eff_SNP

```
g <- hg #or SNP when assigning the effect to the SNP, coded in 0/1

g_alt <- SNP #or hg when assigning the effect to the SNP, coded in 0/1

a <- eff_hg #or eff_SNP when assigning the effect to the SNP

G <- a*g

beta <- c()

for (i in 1:1000){

E <- rnorm_pre(G, mu = -mean(G), sd = sqrt(1-var(G)), r = 0, empirical = TRUE)

P <- G + E

fit <- lm(P ~ g_alt)

beta[i] <- summary(fit)$coefficients[2,1]

}

pval <- mean(beta <= eff_hg)

#use >= when observed effect locates in the right half of the distribution
```

Nuclear DNA Imputation QC

We transferred bgen file to PLINK file and extracted the imputed data for our initial 332,047 unrelated white British samples in PLINK2[2]. Taking QC chromosome 1 as an example, the script used is a follow. SNP INFO and sample genetic ancestry information were provided by UK biobank (Resource 1967 & 531).

```
plink2 --bgen chr1.bgen ref-first \ #input imputation file
```

```
--extract qc1/qc1.chr1.snplist \ #input a list of SNP with INFO > 0.3

--geno 0.05 \

--hwe 1e-50 \

--keep unrelated_wb_id.txt \ #input a list of individuals that are white British

--maf 0.0001 \

--make-pgen \

--mind 0.1 \

--out chr1 \

--oxford-single-chr 1 \

--sample chr1.sample \ #input sample file

--threads 64
```

Nuclear DNA GWAS (nGWAS)

nGWAS, including haplogroup-stratified GWAS, MT-nDNA interaction and co-segregation GWAS, was conducted in PLINK2, without splitting UKBB and UKBL subsets. An example code for running GWAS on chromosome 1 is provided below.

#haplogroup-stratified GWAS

```
plink2 --pfile chr1 \ #input chr1.pgen file

--pheno phe.residuals \#input covariate adjusted phenotype

--keep T.id \ #list of individuals belong to T haplogroup

--threads 64 \

--glm allow-no-covars \

--out chr1_T #output name
```

#MT-nDNA interaction

```
plink2  --covar is.T.cov \ #input covariate file, 1 for being T haplogroup, 0 for not

--extract sig.snps \# a list of SNP

--glm interaction \

--no-psam-pheno \

--pfile chr1 \ #input chr1.pgen file

--pheno phe.residuals \#input covariate adjusted phenotype

--tests 1,3 \test beta and interaction

--out chr_T_interaction #output name
```

The script for co-segregation GWAS was similar to haplogroup-stratified GWAS without --keep command to remove individuals.

MAGMA

Gene-based and gene-set analyses were performed in MAGMA v1.09.

#step 1, annotate 1000G EUR reference panel

```
magma --annotate --snp-loc g1000.bim --gene-loc NCBI37.3.gene.loc --out 1000g
```

#step 2. Derive gene-based statistic using all SNPs from the summary

```
magma --bfile g1000_eur \

--pval inputgwas ncol=N \

--gene-annot 1000g.genes.annot \

--out output
```

#the inputgwas file contains three columns with heading, SNP, N and P.

#step 3. Derive a competitive p value for a gene set

```
magma \

--gene-results input.based.genes.raw \ #output from step 2

--set-annot gene_set col=1,2 \ #without heading, column 1= gene id and 2= set_name

--model self-contained \

--out output
```

LD Score regression (LDSC)

LD score regression was used to estimate the genetic correlation between two GWAS summaries[3, 4].

#step 1, reformat GWAS summary

```
munge_sumstats.py --sumstats  input \

--N-col n \ #column number of sample size

--a1 a1 \ #column number of A1 allele

--a2 a2 \ #column number of A2 allele

--signed-sumstats z,0 \ #column number or beta/OR/T-stats/Z-stats

--merge-alleles w_hm3.snplist \

--chunksize 500000 \

--out output
```

#step2, estimate rg between two GWAS

```
ldsc.py --rg gwas1.sumstats.gz, gwas2.sumstats.gz \

--ref-ld-chr ../ldsc/eur_w_ld_chr/ \ #pre-calculated LD score from LDSC website

--w-ld-chr ../ldsc/eur_w_ld_chr/ \

--out output
```

Multi-Trait-based Conditional and Joint (mtCOJO)

mtCOJO was run using the following command in GCTA[5, 6].

```
gcta64 --bfile 1000G.EUR.QC.ALL.hm3 \ #1000G EUR data

--mtcojo-file gwas.list \ #input file pointing the location of GWAS

--ref-ld-chr eur_w_ld_chr/ \

--w-ld-chr eur_w_ld_chr/ \

--threads 64 \

--heidi-thresh 0 \

--gsmr-snp-min 1 \

--out output name
```

gwas.list is a file made by two rows and two columns. The first column is the name of the trait and the second column is the path to the GWAS file, with H-haplogroup-stratified GWAS being on the first row.

Power calculation for co-segregation GWAS

Derivations

The concept of co-segregation GWAS is essentially testing whether there is a difference in allele frequency between cases (i.e. tested haplogroup) and controls (i.e. non-tested groups) using linear regression. Assuming having N samples in a population, k proportion are cases and $(1-k)$ proportion are controls. Assuming a SNP is under Hardy-Weinberg equilibrium (i.e. under random matting) in both cases and controls with allele frequency of p_1 and p_0, respectively. The contingency table under these assumptions is shown in **Supplementary Table 1**.

Supplementary Table 1. 2 x 3 contingency table under co-segregation GWAS assumptions.

		Genotype (X)		
		0	1	2
Haplogroup (Y)	Tested	$(1-p_1)^2kN$	$2p_1(1-p_1)kN$	p_1^2kN
	Non-tested	$(1-p_0)^2(1-k)N$	$2p_0(1-p_0)(1-k)N$	$p_0^2(1-k)N$

In simple linear regression of regressing Y on X, the linear regression coefficient and its t-statistics (for testing $H_0{:}\,\hat{\beta}=0;\ H_1{:}\,\hat{\beta}\neq 0$) are as the following, $\hat{\beta}=r_{xy}\frac{S_y}{S_x}$ and $t=\frac{r_{xy}\sqrt{N-2}}{\sqrt{1-r_{xy}^2}}$, where $r_{xy}=\frac{N\sum x_iy_i-\sum x_i\sum y_i}{\sqrt{N\sum x_i^2-(\sum x_i)^2}\sqrt{N\sum y_i^2-(\sum y_i)^2}}$. Here the phenotype Y is 1 for tested group and 0 for non-tested group, and X is the genotype of a SNP in dosage. The expectation of each item (e.g. $\sum x_i$) involved in the equations can be calculated using the contingency table provided above. The detailed calculation is shown for $\sum x_i$ as below.

$$\sum x_i = 0\times[(1-p_1)^2kN+(1-p_0)^2(1-k)N]+1\times[2p_1(1-p_1)kN+2p_0(1-p_0)(1-k)N] + 2\times[p_1^2kN+p_0^2(1-k)N] = 2p_1kN+2p_o(1-k)N$$

For the rest item, their expectations are as the following

$$\sum y_i = kN$$

$$\sum x_iy_i = 2p_1kN$$

$$\sum x_i^2 = 2p_1(1+p_1)kN+2p_0(1+p_0)(1-k)N$$

$$\sum y_i^2 = kN$$

Thereby, the expectations of r_{xy} and t are

$$r_{xy}=\frac{\sqrt{2k(1-k)}(p_1-p_0)}{\sqrt{p_1k(1+p_1-2p_1k)-4p_0p_1k(1-k)+p_o(1-k)[1+p_0-2p_o(1-k)]}}$$

$$t=\frac{\sqrt{2k(1-k)(N-2)}(p_1-p_0)}{\sqrt{\sqrt{p_1(1-p_1)k+p_0(1-p_0)(1-k)}}}$$

Simulation shows that, for a given marker and population (i.e. p_0, p_1, k, and N are known), the observed values and theoretical expected values converged for r_{xy} and t. (Figures 1-2)”

Simulation

In each simulation, we simulated the genotype of N samples (N was set to 324,488, the sample size for co-segregation GWAS including those without phenotypes). For kN samples, the genotype were sampled from 0, 1 or 2 with probabilities of $(1-p_1)^2$, $2p_1(1-p_1)$ and p_1^2. For the rest $(1-k)N$ samples, the genotype were sampled from 0, 1 or 2 with probabilities of $(1-p_0)^2$, $2p_0(1-p_0)$ and p_0^2. k, p_1 and $p_0 \in (0,0.5]$ and the numbers were randomly generated in each simulation. We regressed the phenotype (0 for the $(1-k)N$ subset and 1 for the kN subset) on simulated genotype to get the t-statistics and the coefficient of determination. We performed 1000 replications and compared the observed values from simulated data with the theoretical expectations directly calculated from simulation parameters. Results (**Supplementary Figures 56 & 57**) show that the expected values and observed values perfectly converged at line $y = x$, suggesting the math equations derived are accurate.

Supplementary Figure 57. Comparing r_{xy}. Expected and observed values converged at line $y = x$ (red dotted).

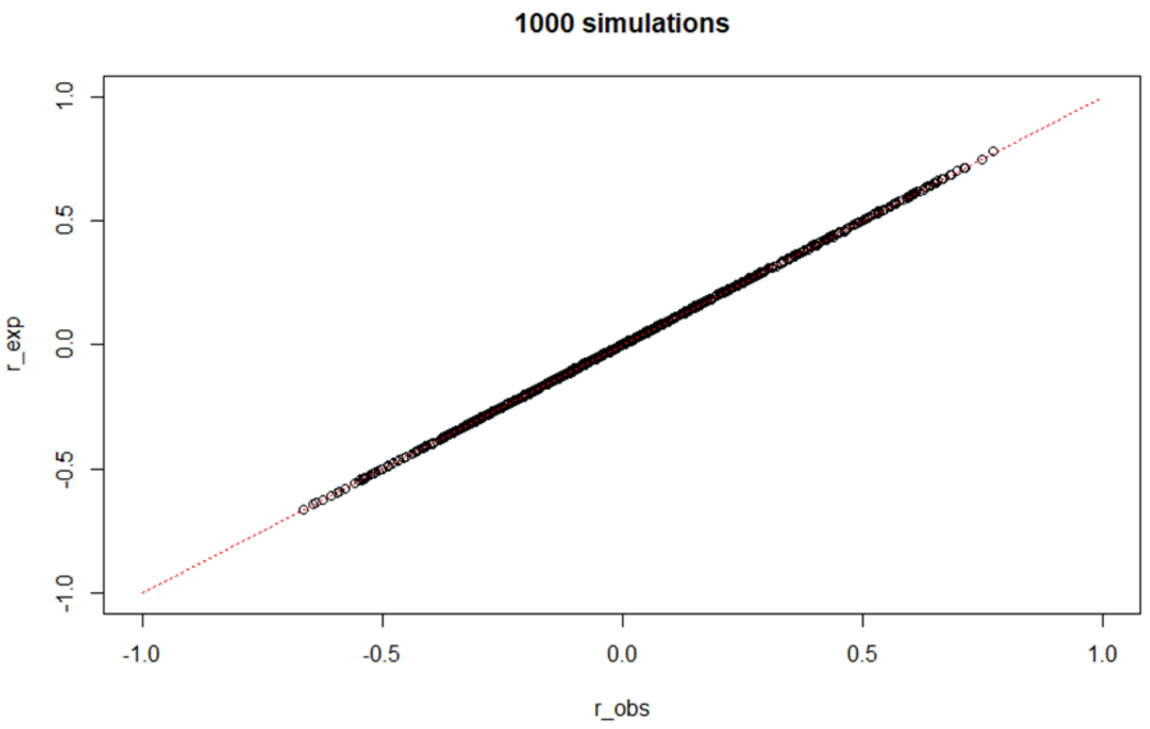

Supplementary Figure 58. Comparing t. Expected and observed values converged at line $y = x$ (red dotted).

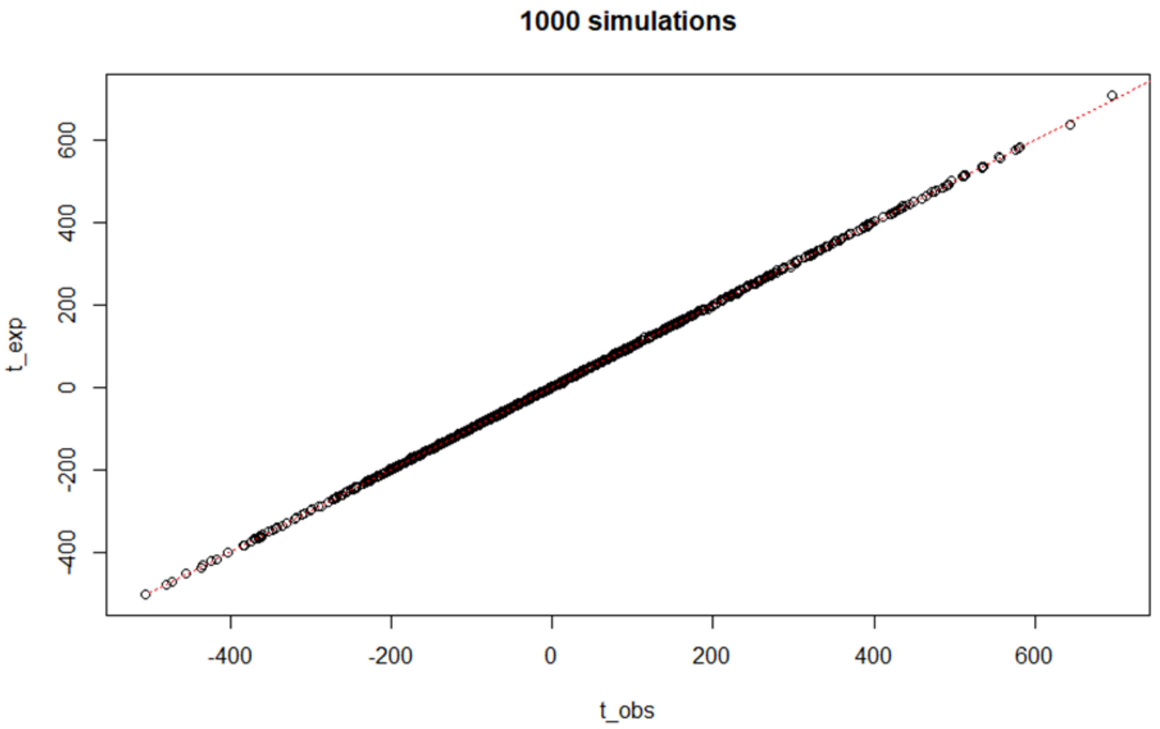

Power calculation

Based on the equation derived above, for each haplogroup, we calculated the power for SNPs across MAF spectrum (**Supplementary Figures 59**). Results show that the detection power is 100% for any SNPs with frequency difference greater than 5% in UK Biobank, regardless of MAF and haplogroups.

Supplementary Figures 59. Power calculation for co-segregation GWAS.

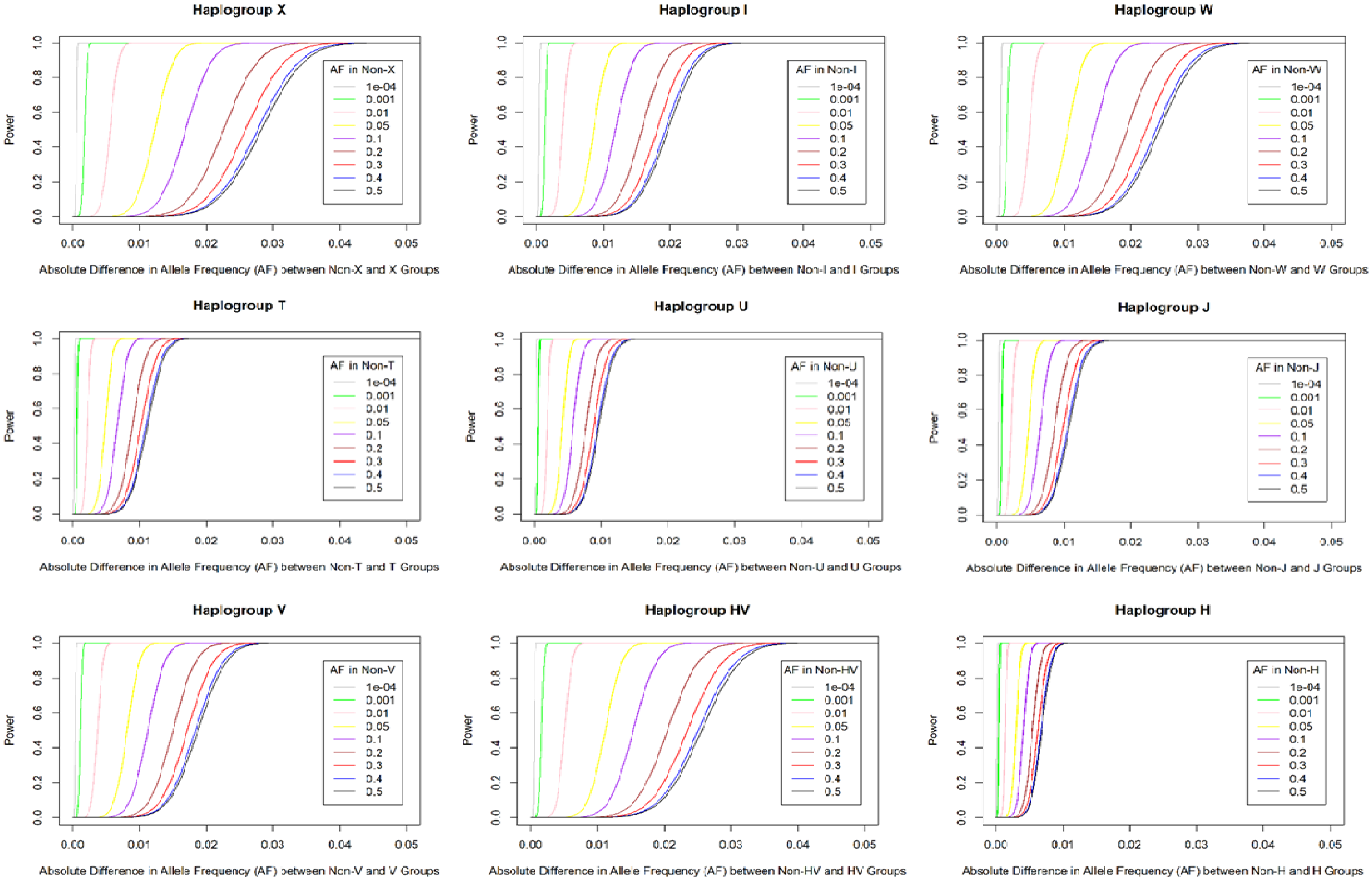

R code

#simulation to confirm the math equations for power calculation

```
n <- 324488

P0 <- c()

P1 <- c()

K <- c()

T_obs <- c()

R_obs <- c()

for (x in 1:1000){

k <- sample(c(1:5000),1)/10000

K[x] <- k

n1 <- round(k*n,0)

n0 <- n - n1

P <- c(rep(1,n1),rep(0,n0))

af1 <- sample(c(1:5000),1)/10000

af0 <- sample(c(1:5000),1)/10000

g1 <- sample(c(0,1,2),n1,prob=c((1-af1)^2,2*af1*(1-af1),af1^2),replace=T)

g0 <- sample(c(0,1,2),n0,prob=c((1-af0)^2,2*af0*(1-af0),af0^2),replace=T)

g <- c(g1,g0)

P1[x] <- af1

P0[x] <- af0

fit <- lm(P ~ g)

T_obs[x] <- summary(fit)$coefficients[2,3]

R_obs[x] <- cor.test(P,g)$est

rm(k,n1,n0,P,af1,af0,g1,g0,g,fit)

}

rm(x)

sum_xy <- 2*P1*K*n

sum_x <- 2*P1*K*n + 2*P0*(1-K)*n
```

```
sum_y <- K*n
sum_xx <- 2*P1*(1+P1)*K*n + 2*P0*(1+P0)*(1-K)*n
sum_yy <- K*n
r_exp <- (n * sum_xy - sum_x * sum_y)/sqrt( (n*sum_xx - sum_x^2)*(n*sum_yy-sum_y^2))
plot(r_exp ~ R_obs,ylim=c(-1,1),xlim=c(-1,1),xlab="r_obs",ylab="r_exp",main="1000 simulations")
segments(-1,-1,1,1,col="red",lty="dotted")
t_exp <- r_exp*sqrt(n-2)/sqrt(1-r_exp^2)
plot(t_exp ~ T_obs,xlab="t_obs",ylab="t_exp",main="1000 simulations")
segments(-500,-500,800,800,col="red",lty="dotted")
```

#power calculation and plots

```
n <- 324488
hg <- c("H","HV","V","J","T","U","K","I","W","X")
N0 <- c(185779,318706,313569,288324,291469,278266,295986,314610,318057,319764)
N1 <- n - N0
K <- rep(N1/n,each=45000)
P0 <- rep(rep(c(0.0001,0.001,0.01,0.05,0.1,0.2,0.3,0.4,0.5),each=5000),10)
P1 <- rep(rep(c(1:5000)/10000,9),10)
sum_xy <- 2*P1*K*n
sum_x <- 2*P1*K*n + 2*P0*(1-K)*n
sum_y <- K*n
sum_xx <- 2*P1*(1+P1)*K*n + 2*P0*(1+P0)*(1-K)*n
sum_yy <- K*n
r_exp <- (n * sum_xy - sum_x * sum_y)/sqrt( (n*sum_xx - sum_x^2)*(n*sum_yy-sum_y^2))
t_exp <- r_exp*sqrt(n-2)/sqrt(1-r_exp^2)
z_half_a <- -qnorm(5*10^-8/2,0,1)
pw <- 1 - pnorm(z_half_a-abs(t_exp),0,1,lower.tail=T)
P_D <- abs(P1 - P0)
```

```
my_col <- c("grey","green","pink","yellow","purple","brown","red","blue","black")

for (i in 1:10){

  tiff(paste0("power_co_seg_gwas_",hg[i],".tiff"),width=16,height=12,units="cm",res=300)

  temp_idx <- 1:45000 + 45000*(i-1)

  j <- 1

  temp_idx2 <- 1:5000 + 5000*(j-1)

plot(pw[temp_idx][temp_idx2] ~
P_D[temp_idx][temp_idx2],ylim=c(0,1),xlim=c(0,0.05),type="l",,ylab="Power",xlab=paste0("Absolu
te Difference in Allele Frequency (AF) between Non-",hg[i]," and ",hg[i], "
Groups"),main=paste0("Haplogroup ",hg[i]),col=my_col[j])

  for (j in 2:9){

    temp_idx2 <- 1:5000 + 5000*(j-1)

    points(pw[temp_idx][temp_idx2] ~ P_D[temp_idx][temp_idx2],type="l",col=my_col[j])

  }

  legend(0.0375, 0.9,legend=unique(P0),title=paste0("AF in Non-",hg[i]),col=my_col,lty=1)

  dev.off()

}
```

Power calculation for interaction GWAS

Derivations

Assuming in a random matting population of N samples, a causal variant has an effect of a on the phenotype y. The phenotype can be written as $y = ax + e$, where the genotype $x \in B(2, p)$ with p being the allele frequency of reference allele, and unmeasured residuals $e \in N(0, \sigma_e^2)$. Assuming no genotype-by-environmental correlation, $\sigma_e^2 = \sigma_y^2 - a^2\sigma_x^2 = \sigma_y^2 - 2p(1-p)a^2$. Let $a = k\sigma_y$ (i.e. effect size a in SD unit of y), $\sigma_e^2 = \sigma_y^2[1 - 2p(1-p)k^2]$. Under these assumptions, when regressing phenotype y on genotype x, the expectation of regression coefficient is $\beta = a = k\sigma_y$ and its standard error is $se(\beta) = \frac{1}{\sqrt{N-2}}\frac{\sigma_e}{\sigma_x} = \frac{\sigma_y}{\sqrt{N-2}}\frac{\sqrt{1-2p(1-p)k^2}}{\sqrt{2p(1-p)}}$ with $k^2 < \frac{1}{2p(1-p)}$.

In our method, we first ran GWAS stratified by mt-haplogroups and then compared the beta of test-group-stratified GWAS with that of H-group-stratified GWAS as an initial scan of potential interaction. Two hypothesis are, $H_0: \widehat{\beta_1} = \widehat{\beta_2}$; $H_1: \widehat{\beta_1} \neq \widehat{\beta_2}$. The statistics of the test is $Z = \frac{\widehat{\beta_1} - \widehat{\beta_2}}{\sqrt{se(\widehat{\beta_1})^2 + se(\widehat{\beta_2})^2}}$ and its expectation is $Z = \frac{k_1\sigma_{y_1} - k_2\sigma_{y_2}}{\sqrt{\frac{[1-2p_1(1-p_1)k_1^2]\sigma_{y_1}^2}{2p_1(1-p_1)(N_1-2)} + \frac{[1-2p_2(1-p_2)k_2^2]\sigma_{y_2}^2}{2p_2(1-p_2)(N_2-2)}}}$. When p_1, p_2, k_1, k_2, $\sigma_{y_1}^2$, $\sigma_{y_2}^2$, N_1, and $N_2$are known, the distribution of H_1 is centred at Z. Thus, the power to reject H_0 at 1×10^{-5} (i.e. $Z_{a/2} = 4.42$) is, $Power = 1 - \Pr(X \leq t) = 1 - \phi(4.42 - |Z|)$.

Here, we add more realistic assumptions before power calculation. First, we assume that there is no difference in phenotype variance, $\sigma_{y_1}^2 = \sigma_{y_2}^2 = \sigma_y^2$. Second, we assume that there is no difference in allele frequency, $p_1 = p_2 = p$, as markers with frequency difference between haplogroups should be identified by co-segregation GWAS. Simplified expectation of Z is $\frac{k_1 - k_2}{\sqrt{\frac{1-2p(1-p)k_1^2}{2p(1-p)(N_1-2)} + \frac{1-2p(1-p)k_2^2}{2p(1-p)(N_2-2)}}}$.

Simulations (**Supplementary Figures 60-62**) confirms that the expected and theoretical values of β, $se(\beta)$, and Z converged. And power calculations for each haplogroup see Figures 7.

Simulation

In each simulation, we simulated the genotype of N_1 and N_2 samples, where N_1 and N_2 are the sample size of H haplogroup and any tested haplogroup in real data used in interaction GWAS. For N_1 samples, the genotype were sampled from 0, 1 or 2 with probabilities of $(1-p)^2$, $2p(1-p)$ and p^2. The genetic effect was calculated as genotype multiples the standardised effect size k_1. The unmeasured residuals were samples from normal distribution with mean of 0 and variance of $1 - 2p(1-p)\,k_1^2$. Phenotype was calculated as the sum of genetic effect and unmeasured residuals.

Similar for N_2 samples. p, k_1 and $k_2 \in (0,0.5]$ and the numbers were randomly generated in each simulation. We regressed the phenotype on simulated genotype to get regression coefficient and its standard error in N_1 and N_2 samples separately, and then tested whether the regression coefficients are differently from each other. We performed 1000 replications and compared the observed values from simulated data with the theoretical expectations directly calculated from simulation parameters. Results show that the expected values and observed values converged at line $y = x$, suggesting the math equations derived are accurate.

Supplementary Figure 60. Comparing β. Expected and observed values converged at line $y = x$ (red dotted).

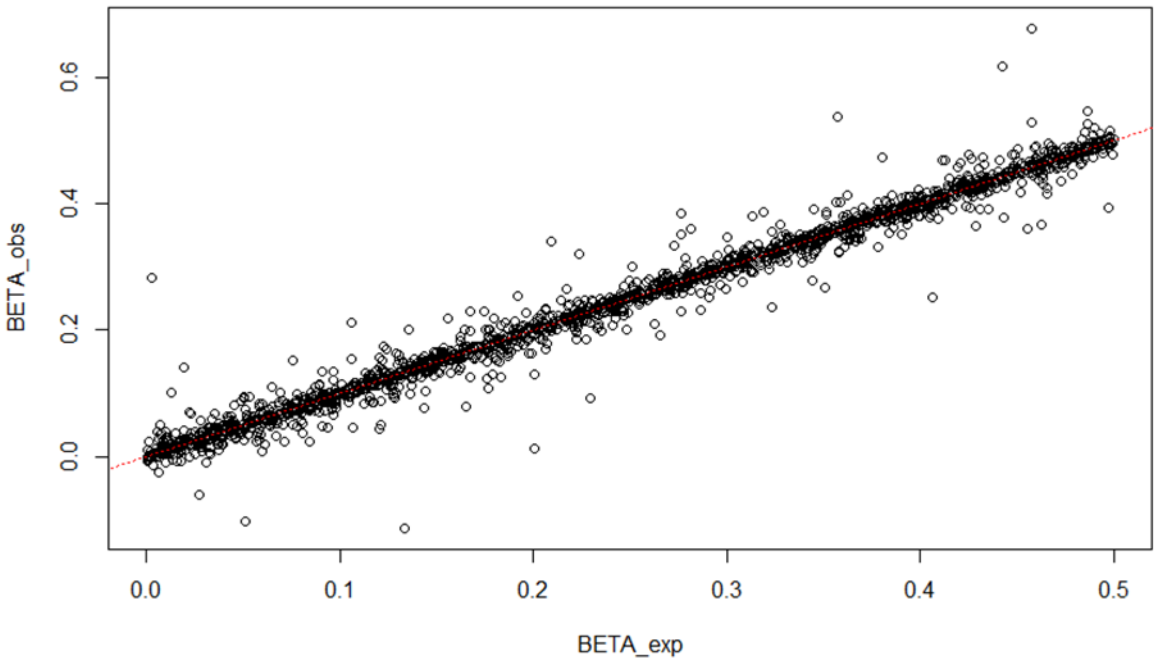

Supplementary Figure 61. Comparing $se(\beta)$. Expected and observed values converged at line $y = x$ (red dotted).

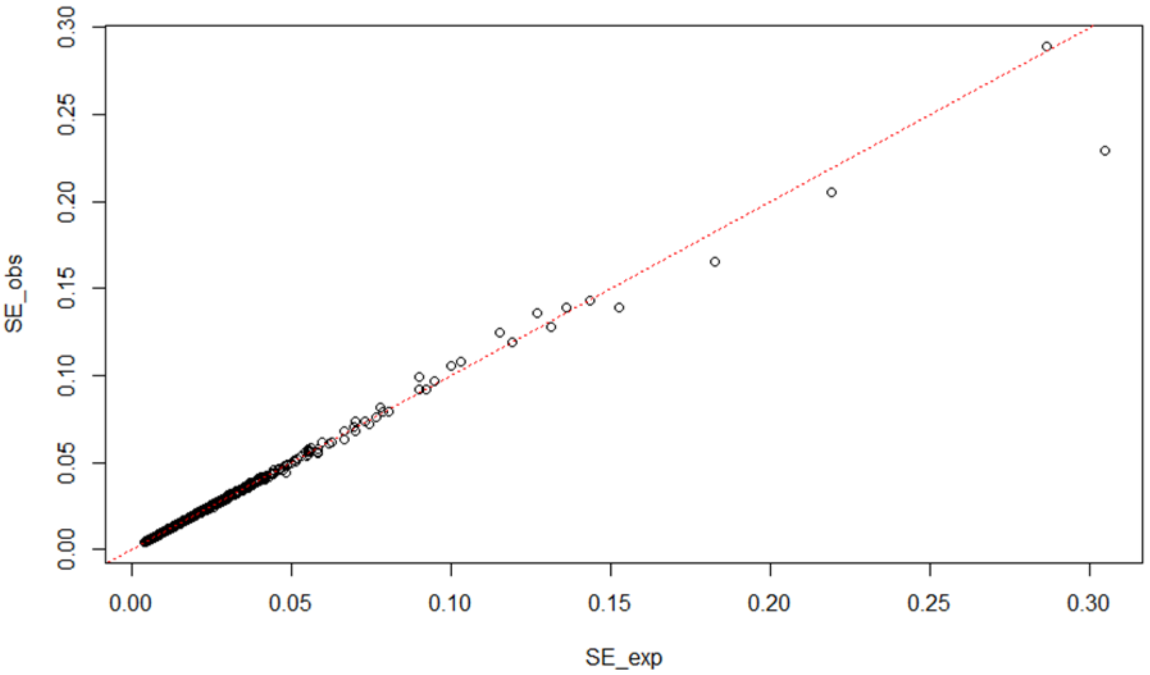

Supplementary Figure 62. Comparing Z statistics of testing the difference in β between two groups. Expected and observed values converged at line $y = x$ (red dotted).

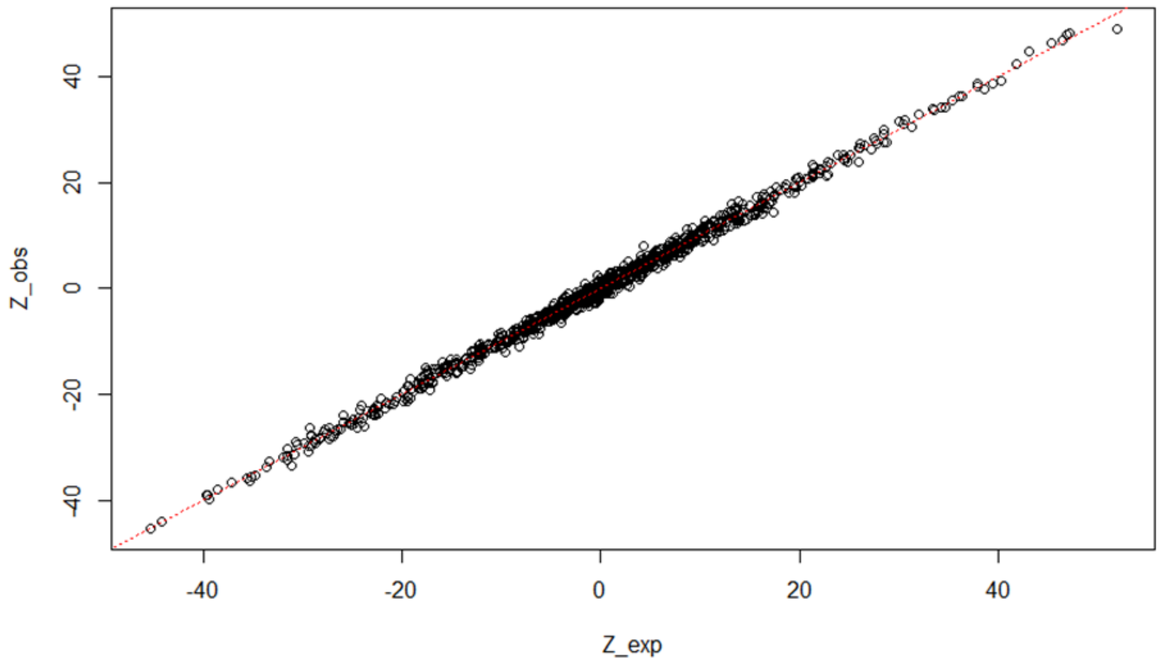

Power calculation

Based on the equation derived above, we calculated the power to detection the beta difference for SNPs across MAF spectrum with different sizes of the effect in each haplogroups (**Supplementary Figure 63**).

Supplementary Figures 63. Power calculation for interaction GWAS.

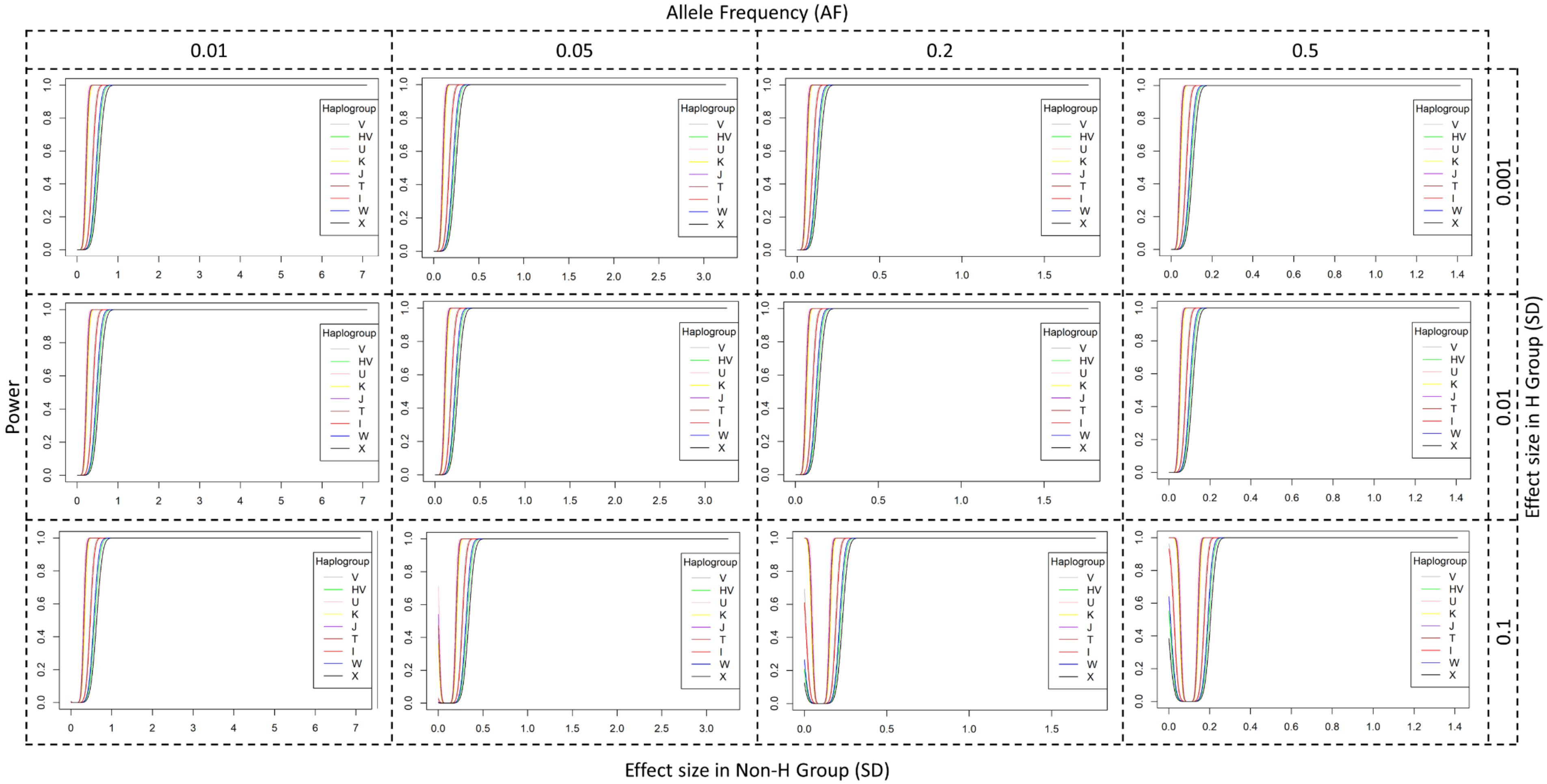

R code

#simulation to confirm the math equations for power calculation, interaction

```
n1 <- 112793
hg <- c("V","HV","U","K","J","T","I","W","X")
n2 <- c(8818,4687,37627,23131,29464,26893,8025,5176,3851)
P <- c()
K1 <- c()
K2 <- c()
N2 <- c()
BETA1_obs <- c()
BETA2_obs <- c()
SE1_obs <- c()
SE2_obs <- c()
Z_obs <- c()
for (x in 1:1000){
  k1 <- sample(c(1:5000),1)/10000
  k2 <- sample(c(1:5000),1)/10000
  K1[x] <- k1
  K2[x] <- k2
  af <- sample(c(1:5000),1)/10000
  P[x] <- af
  N2[x] <- sample(n2,1)
  g1 <- sample(c(0,1,2),n1,prob=c((1-af)^2,2*af*(1-af),af^2),replace=T)
  g2 <- sample(c(0,1,2),N2[x],prob=c((1-af)^2,2*af*(1-af),af^2),replace=T)
  e1 <- rnorm(n1,0,sqrt(1-2*af*(1-af)*k1^2))
  e2 <- rnorm(N2[x],0,sqrt(1-2*af*(1-af)*k2^2))
  Phe1 <- g1*k1 + e1
  Phe2 <- g2*k2 + e2
```

```
  fit1 <- lm(Phe1 ~ g1)

  BETA1_obs[x] <- summary(fit1)$coefficients[2,1]

  SE1_obs[x] <- summary(fit1)$coefficients[2,2]

  fit2 <- lm(Phe2 ~ g2)

  BETA2_obs[x] <- summary(fit2)$coefficients[2,1]

  SE2_obs[x] <- summary(fit2)$coefficients[2,2]

  Z_obs[x] <- (BETA1_obs[x]-BETA2_obs[x])/sqrt(SE1_obs[x]^2 + SE2_obs[x]^2)

  rm(af,e1,e2,g1,g2,k1,k2,Phe1,Phe2,fit1,fit2)

}

rm(x)



BETA_obs <- c(BETA1_obs,BETA2_obs)

BETA_exp <- c(K1,K2)

SE_obs <- c(SE1_obs,SE2_obs)

SE_exp <- c(1/sqrt(n1-2)*sqrt(1-2*P*(1-P)*K1^2)/sqrt(2*P*(1-P)),1/sqrt(N2-2)*sqrt(1-2*P*(1-
P)*K2^2)/sqrt(2*P*(1-P)))

Z_exp <- (K1 - K2)/sqrt( 1/(n1-2)*(1-2*P*(1-P)*K1^2)/(2*P*(1-P)) + 1/(N2-2)*(1-2*P*(1-
P)*K2^2)/(2*P*(1-P)))

f <- K2/K1

Z_exp2 <- (1-f)*K1*sqrt(2*P*(1-P))*sqrt(n1-2)*sqrt(N2-2)/sqrt(n1 + N2 - 4 - 2*P*(1-P)*K1^2*(N2-
2+f^2*(n1-2)))

plot(BETA_obs ~ BETA_exp)

segments(-1,-1,1,1,col="red",lty="dotted",main="1000 Simulatoins")

plot(SE_obs ~ SE_exp)

segments(-1,-1,1,1,col="red",lty="dotted",main="1000 Simulatoins")

plot(Z_obs ~ Z_exp)

segments(-100,-100,100,100,col="red",lty="dotted",main="1000 Simulatoins")

summary(lm(Z_obs ~ Z_exp))

plot(Z_exp2 ~ Z_exp)

segments(-100,-100,100,100,col="red",lty="dotted",main="1000 Simulatoins")
```

#power calculation

```
n1 <- 112793

hg <- c("V","HV","U","K","J","T","I","W","X")

N2 <- c(8818,4687,37627,23131,29464,26893,8025,5176,3851)

K1 <- c(0.001,0.01,0.1)

P <- c(0.01,0.05,0.2,0.5)

z_half_a <- -qnorm(5*10^-6/2,0,1)

my_col <- c("grey","green","pink","yellow","purple","brown","red","blue","black")

for (k1 in K1){

  for (af in P){

    tiff(paste0("power_interaction_kH",k1,"_af",af,".tiff"),width=16,height=12,units="cm",res=300)

    idx <- 1

    K2 <- c(1:floor(1/sqrt(2*af*(1-af))*1000))/1000

    n2 <- N2[idx]

    Z_exp <- (k1 - K2)/sqrt( 1/(n1-2)*(1-2*af*(1-af)*k1^2)/(2*af*(1-af)) + 1/(n2-2)*(1-2*af*(1-af)*K2^2)/(2*af*(1-af)))

    pw <- 1 - pnorm(z_half_a-abs(Z_exp),0,1,lower.tail=T)

    plot(pw ~ K2,type="l",col=my_col[idx],ylim=c(0,1),xlab="Effect size in non-H group",ylab="Power",

         main="1000 Simulations",sub=paste0("Effect size in H group:",k1," SD; Allele frequency: ",af))

    for (idx in 2:9){

      n2 <- N2[idx]

      Z_exp <- (k1 - K2)/sqrt( 1/(n1-2)*(1-2*af*(1-af)*k1^2)/(2*af*(1-af)) + 1/(n2-2)*(1-2*af*(1-af)*K2^2)/(2*af*(1-af)))

      pw <- 1 - pnorm(z_half_a-abs(Z_exp),0,1,lower.tail=T)

      #points(pw ~ K_ratio,type="l",col=my_col[idx])

      points(pw ~ K2,type="l",col=my_col[idx])

    }

    legend("right",legend=hg,title=paste0("Haplogroup"),col=my_col,lty=1)

    dev.off()
```

```
  }

}
```

Supplementary References

1. R: A Language and Environment for Statistical Computing. . 2020, Accessed Date Accessed 2020 Accessed.

2. Chang CC, Chow CC, Tellier LC, Vattikuti S, Purcell SM, Lee JJ. Second-generation PLINK: rising to the challenge of larger and richer datasets. *Gigascience* 2015; **4:** 7-7.

3. Bulik-Sullivan B, Finucane HK, Anttila V, Gusev A, Day FR, Loh P-R *et al.* An atlas of genetic correlations across human diseases and traits. *Nature Genetics* 2015; **47:** 1236.

4. Bulik-Sullivan BK, Loh P-R, Finucane HK, Ripke S, Yang J, Schizophrenia Working Group of the Psychiatric Genomics C *et al.* LD Score regression distinguishes confounding from polygenicity in genome-wide association studies. *Nature Genetics* 2015; **47:** 291.

5. Zhu Z, Zheng Z, Zhang F, Wu Y, Trzaskowski M, Maier R *et al.* Causal associations between risk factors and common diseases inferred from GWAS summary data. *Nature Communications* 2018; **9**(1)**:** 224.

6. Yang J, Lee SH, Goddard ME, Visscher PM. GCTA: a tool for genome-wide complex trait analysis. *The American Journal of Human Genetics* 2011; **88**(1)**:** 76-82.

7. Hill WD, Weiss A, Liewald DC, Davies G, Porteous DJ, Hayward C *et al.* Genetic contributions to two special factors of neuroticism are associated with affluence, higher intelligence, better health, and longer life. *Mol Psychiatry* 2020; **25**(11)**:** 3034-3052.
