# Supplementary material for: The contributions of mitochondrial and nuclear mitochondrial genetic variation to neuroticism
Source: Nat Commun. 2023 May 30;14:3146. doi: 10.1038/s41467-023-38480-y (PMC10229642; doi:10.1038/s41467-023-38480-y)
Supplement: Supplementary file 2 — Description of Additional Supplementary Files [file 41467_2023_38480_MOESM2_ESM.pdf]

Description of Additional Supplementary Files

File name: **Supplementary Data 1.**
Description: Showing the variance explained by common SNPs on the autosomes and those on the mitochondrial genome. Stratified linkage disequilibrium score regression was used to estimate the per chromosome heritability of each of the factors of neuroticism. Regression was used to estimate the variance explained by haplogroup for the mitochondrial genome.

File name: **Supplementary Data 2.**
Description: Model 1 describing the association between haplogroup and super haplogroup with the three neuroticism traits. Haplogroup H and super haplogroup HV were used as the reference group when examining the effect of haplogroup and super haplogroup respectively. P values (two-sided t-test) shown in bold passed the significance threshold set at 0.005.

File name: **Supplementary Data 3.**
Description: Model 2 describing the association between haplogroup and super haplogroup with the three neuroticism traits. Here each tested haplogroup (and super haplogroup) was compared to the remaining haplogroups to determine its association. P values (two-sided t-test) shown in bold passed the significance threshold set at 0.005.

File name: **Supplementary Data 4.**
Description: MT-GWAS results for the general factor of neuroticism. Sample sizes and associations are shown for each the UKBB and UKBL arrays separately as well as the final meta-analytic results. N_Study indicates the number of studies (i.e. UKBB and UKBL) that the SNP passed quality control checks in.

File name: **Supplementary Data 5.**
Description: MT-GWAS results for the anxiety/tension special factor of neuroticism. Sample sizes and associations are shown for each the UKBB and UKBL arrays separately as well as the final meta-analytic results. N_Study indicates the number of studies (i.e. UKBB and UKBL) that the SNP passed quality control checks in.

File name: **Supplementary Data 6.**
Description: MT-GWAS results for the worry/vulnerability special factor of neuroticism. Sample sizes and associations are shown for each the UKBB and UKBL arrays separately as well as the final meta-analytic results. N_Study indicates the number of studies (i.e. UKBB and UKBL) that the SNP passed quality control checks in.

File name: **Supplementary Data 7.**
Description: Table comparing the mtSNP associations identified for Worry/Vulnerability with those identified in Yonova-Dong et al. (2021). P values in column F are from our GWAS results derived in PLINK using two-sided t-test. P values in M are extracted from Yonova-Doing et al. (2021).

File name: **Supplementary Data 8.**
Description: Table comparing the haplogroup associations identified for Worry/Vulnerability and the General Factor of Neuroticism with the haplogroup defining markers found in Yonova-Dong et al. (2021). P values in column F are from our GWAS results derived in PLINK using two-sided t-test. P values in M are extracted from Yonova-Doing et al. (2021).

File name: **Supplementary Data 9.**
Description: Phenotypic and genetic correlations between the three factors of neuroticism with blood measures in UK Biobank. GWAS summary data for the three factors of neuroticism was obtained from Hill et al. (2020) and GWAS summary data on each of the blood measures was taken from http://www.nealelab.is/uk-biobank. Pearson correlation was calculated for phenotypic correlation. Genetic correlations were derived using LDSC regression and two-sided Z test was used. MPV = mean platelet volume; PCT = plateletcrit; RBC = red blood cell count; MCV = mean corpuscular volume; HCT = hematocrit; MCH = mean corpuscular hemoglobin; RDW = red blood cell width; LYMPH = lymphocyte count; WBC = white blood cell count; eGFRcr = Estimated Glomerular Filtration Rate CKD-EPI creatinine equation from PMID:22762315.

File name: **Supplementary Data 10.**
Description: Showing significantly associated (P < 0.05/2099) gene-level statistics derived in MAGMA using autosomal SNPs for the three factors of neuroticism. P values of two-sided Z test were reported here.

File name: **Supplementary Data 11.**
Description: Functional annotation of autosomal mt-nDNA genes associated with the factors of neuroticism. N_genes indicates the number of genes in the gene set and N_overlap indicates the number of genes from the set that were also associated with the neuroticism traits. The column 'P' is raw p vales of Fisher's exact test and the column 'adjP' is its FDR adjusted p values. The column genes indicates the gene name from the set that was associated with the neuroticism traits.

File name: **Supplementary Data 12.**
Description: Gene-set analysis of MT-nDNA candidate genes for neuroticism GWAS. Number of Genes indicate the number of genes in the gene set that overlap with the GWAS. MAGMA gene-set competitive test (equivalent to a one-sided two-sample t-test) were used here.

File name: **Supplementary Data 13.**
Description: Autosomal genetic correlations between haplogroup stratified GWAS. P_{rg}<1 indicates if a p value describing if the genetic correlations is significantly different from 1 using two-sided Z test.

File name: **Supplementary Data 14.**
Description: Showing the genetic correlations derived for the special factor of Worry/Vulnerability with 21 traits by H-haplogroup. P_diff shows a p value derived by comparing two genetic correlations to examine the differences between the two. All = everyone. Is_H = H-haplogroup individuals. Not_H = non-H-haplogroup individuals. adj_H = H-haplogroup autosomal effects conditioned on non-H-haplogroup autosomal effects. Two-sided Z test was performed to test whether rg_1 and rg_2 were significantly different from 0 (P_1 & P_2). Two-sided two-sample T-test was performed to test whether rg_1 and rg_2 is different from each other (P_diff).

File name: **Supplementary Data 15**.
Description: MAGMA gene-based analysis of haplogroup-stratified GWAS. Two-sided Z test was performed. Only significant genes (P < 0.05/18,337) shown.

File name: **Supplementary Data 16.**
Description: Comparison of haplogroup-stratified GWASs results. Only independent markers that reached genome-wide significance level of $5x10^{-8}$ in the haplogroup-stratified GWAS and showed a difference in beta estimates when compared to H-haplogroup-stratified GWAS at $P < 1x10^{-5}$ are shown. Three markers meet these criteria but were in high LD ($r^2 > 0.4$) with the independent SNPs but had weaker association and so were not tested. P: two-sided t-test in PLINK. P_{diff}: two-sided two-sample t-test.

File name: **Supplementary Data 17**.
Description: Results of nDNA-MT interaction for the five independent nDNA in Supplementary Data 16. P: two-sided t-test in PLINK.

File name: **Supplementary Data 18.**
Description: Candidate SNPs in LD ($r^2 > 0.6$) with lead SNP (two of the five pre-selected markers from Supplementary Data 16) detected by FUMA based on LD information in 1000G EUR. gwasP: original GWAS p values (two-sided t-test from PLINK). CADD: CADD score which is computed based on 63 annotations. The higher the score, the more deleterious the SNP is. RDB: RegulomeDB score which is a categorical score (from 1a to 7). 1a is the highest score for SNPs with the most biological evidence to be a regulatory element. minChrState: The minimum 15-core chromatin state across 127 tissue/cell type. commonChrState: The most common 15-core chromatin state across 127 tissue/cell types.

File name: **Supplementary Data 19.**
Description: Genes mapped to each locus by FUMA. Genes were mapped to each locus by positional mapping, eQTL mapping, and chromatin interaction strategies. HUGO: HUGO (HGNC) gene symbol. pLI: probability of loss of function mutation intolerance (higher scores indicate higher probability); ncRVIS: Non-coding residual variation intolerance score. The higher the score is, the more intolerant to noncoding variants the gene is. posMapSNPs (posMap): The number of SNPs mapped to gene based on positional mapping (after functional filtering if parameters are given). posMapMaxCADD (posMap): The maximum CADD score of mapped SNPs by positional mapping. eqtlMapSNPs (eqtlMap): The number of SNPs mapped to the gene based on eQTL mapping. eqtlMapminP (eqtlMap): The minimum eQTL P-value of mapped SNPs. eqtlMapminQ (eqtlMap): The minimum eQTL FDR of mapped SNPs. eqtlMapts (eqtlMap): Tissue types of mapped eQTL SNPs. eqtlDirection (eqtlMap): Consecutive direction of mapped eQTL SNPs after aligning risk increasing alleles in GWAS and tested alleles in eQTL data source. ciMapts (ciMap): Tissue/cell types of mapped chromatin interactions. minGwasP: The minimum P-value of mapped SNPs. IndSigSNPs: rsID of the all independent significant SNPs of mapped SNPs.

File name: **Supplementary Data 20**.
Description: eQTLs detected by FUMA. eQTLs correspond to the eQTL genes mapped to our loci through eQTL mapping strategy in Supplementary Data 19. P: P-value of eQTLs in GTEx.

File name: **Supplementary Data 21.**
Description: Chromatin interaction detected by FUMA. Chromatin interaction regions correspond to the genes mapped through chromatin interaction strategy in Supplementary data 19. intra/inter: Intra- or Inter-chromosomal interaction. SNPs: rsID of candidate SNPs which are overlapping with the region 1. genes: ENSG ID of genes whose promoter regions are overlapped with region 2.

File name: **Supplementary Data 22**.
Description: The three neuroticism phenotypes extracted from the 12 items from the Revised Eysenck Personality Questionnaire using an oblique bi-factor model. Factor loadings are shown for each of the three neuroticism phenotypes. Bold indicates high factor loadings (> 0.3) for each of the special factors of Anxiety/Tension, and Worry/Vulnerability.

File name: **Supplementary Data 23.**
Description: 2,220 MT-nDNA candidate genes and their locations in GRCh37. Candidate genes were sourced from two databases: IMPI and Mitocarta.

File name: **Supplementary Data 24.**
Description: The source of the GWAS data sets used to derive the genetic correlations.

File name: **Supplementary Data 25.**
Description: Co-segregation GWAS results. P: GWAS p values (two-sided t-test from PLINK). Any nDNA markers associate with any MT haplogroup at $P < 1x10^{-5}$ are shown here. No clumping was performed.

File name: **Supplementary Data 26.**
Description: Gene-set analysis on MT-nDNA co-segregation GWAS. Number of Genes indicate the number of genes in the gene set that overlap with the GWAS. P: p values of MAGMA gene-set competitive test (equivalent to a one-sided two-sample t-test). Gene-set enrichment where FDR < 0.05 are in bold.

File name: **Supplementary Data 27.**
Description: Effect of haplogroup-co-segregated marker on neuroticism. Haplogroup = the testing haplogroup. Haplogroup P = the p value of association between the testing haplogroup and the testing trait (two-sided t-test). Haplogroup Analysis Model = the analysis model. HG-co-Segregated Marker Most Associated with the Trait = among all nDNA markers co-segregated with the testing haplogroup at $P < 1x10^{-5}$, which one associate with the testing trait the most. Co-Segregation P = p value in co-segregation GWAS of that nDNA marker and GWAS P = p value in the GWAS of the testing trait of that nDNA marker, both derived by two-sided t-test in PLINK.
