# Supplementary material for: The contributions of mitochondrial and nuclear mitochondrial genetic variation to neuroticism
Source: Nat Commun. 2023 May 30;14:3146. doi: 10.1038/s41467-023-38480-y (PMC10229642; doi:10.1038/s41467-023-38480-y)
Supplement: Supplementary file 30 — Reporting Summary [file 41467_2023_38480_MOESM30_ESM.pdf]

nature portfolio

Corresponding author(s): W. David Hill

Last updated by author(s): Mar 15, 2023

Reporting Summary

Nature Portfolio wishes to improve the reproducibility of the work that we publish. This form provides structure for consistency and transparency in reporting. For further information on Nature Portfolio policies, see our Editorial Policies and the Editorial Policy Checklist.

Statistics

For all statistical analyses, confirm that the following items are present in the figure legend, table legend, main text, or Methods section.

n/a	Confirmed	
☐	☒	The exact sample size (*n*) for each experimental group/condition, given as a discrete number and unit of measurement
☐	☒	A statement on whether measurements were taken from distinct samples or whether the same sample was measured repeatedly
☐	☒	The statistical test(s) used AND whether they are one- or two-sided<br>*Only common tests should be described solely by name; describe more complex techniques in the Methods section.*
☐	☒	A description of all covariates tested
☐	☒	A description of any assumptions or corrections, such as tests of normality and adjustment for multiple comparisons
☐	☒	A full description of the statistical parameters including central tendency (e.g. means) or other basic estimates (e.g. regression coefficient) AND variation (e.g. standard deviation) or associated estimates of uncertainty (e.g. confidence intervals)
☐	☒	For null hypothesis testing, the test statistic (e.g. *F*, *t*, *r*) with confidence intervals, effect sizes, degrees of freedom and *P* value noted<br>*Give P values as exact values whenever suitable.*
☒	☐	For Bayesian analysis, information on the choice of priors and Markov chain Monte Carlo settings
☒	☐	For hierarchical and complex designs, identification of the appropriate level for tests and full reporting of outcomes
☐	☒	Estimates of effect sizes (e.g. Cohen's *d*, Pearson's *r*), indicating how they were calculated

Our web collection on statistics for biologists contains articles on many of the points above.

Software and code

Policy information about availability of computer code

Data collection

No tool was used to collect data.

Data analysis

PLINKv1.90b6.20, https://www.cog-genomics.org/plink/1.9/
PLINK2.00a, https://www.cog-genomics.org/plink/2.0/
SHAPEIT2, https://mathgen.stats.ox.ac.uk/genetics_software/shapeit/shapeit.html
IMPUTE2, https://mathgen.stats.ox.ac.uk/impute/impute_v2.html
Haplogrep2, https://haplogrep.i-med.ac.at/haplogrep2/index.html
R v3.6.1, https://cran.r-project.org/bin/windows/base/old/3.6.1/
LDSC v1.01, https://github.com/bulik/ldsc
MAGMA v1.09, https://ctg.cncr.nl/software/magma
mtCOJO in GCTA v1.92.1. beta6, https://yanglab.westlake.edu.cn/software/gcta/#Overview
STRING, https://string-db.org/
FUMA v1.3.6a, https://fuma.ctglab.nl/

For manuscripts utilizing custom algorithms or software that are central to the research but not yet described in published literature, software must be made available to editors and reviewers. We strongly encourage code deposition in a community repository (e.g. GitHub). See the Nature Portfolio guidelines for submitting code & software for further information.

Data

Policy information about availability of data

All manuscripts must include a data availability statement. This statement should provide the following information, where applicable:

- Accession codes, unique identifiers, or web links for publicly available datasets
- A description of any restrictions on data availability
- For clinical datasets or third party data, please ensure that the statement adheres to our policy

UK Biobank data used in this study are available via the UK Biobank data access process (see http://www.ukbiobank.ac.uk/register-apply/). All GWAS results will be made available through GWAS catalogue upon publicationSummary statistics for MT associations are available in Supplementary Data 4-6. Summary statistics for the autosomal associations are available through GWAS catalog at https://www.ebi.ac.uk/gwas/.

Human research participants

Policy information about studies involving human research participants and Sex and Gender in Research.

Reporting on sex and gender	No analyses conducted were stratified on sex or gender. Self reported biological sex was used as a covariate.
Population characteristics	408,511 participants that were of White British ancestry accoring to the genotype quality control of UK Biobank were included in this study. The study charecteristics can be found in Bycroft et al., Nature 2018 (https://www.nature.com/articles/s41586-018-0579-z).
Recruitment	Please see Bycroft et al., Nature 2018
Ethics oversight	This work was conducted within the approved UK Biobank application number 10279.

Note that full information on the approval of the study protocol must also be provided in the manuscript.

Field-specific reporting

Please select the one below that is the best fit for your research. If you are not sure, read the appropriate sections before making your selection.

☒ Life sciences ☐ Behavioural & social sciences ☐ Ecological, evolutionary & environmental sciences

For a reference copy of the document with all sections, see nature.com/documents/nr-reporting-summary-flat.pdf

Life sciences study design

All studies must disclose on these points even when the disclosure is negative.

Sample size	Sample size for this study was up to 263,883 participants for the single variant and haplogroup association analysis. Sample size was chosen based on the number of individuals in UK Biobank who had completed the neuroticism questionnaire and genotyped MT data.
Data exclusions	Only subjects who completed the neuroticism questionnaire and were of British descent were included in the study.
Replication	A discovery analysis was performed alone due to the lack of a suitable sized replication dataset.
Randomization	Randomisation was not applicable to this study as no data was analysed that would allow identification of any individuals in the study.
Blinding	Blinding is not applicable or necessary in GWAS analyses.

Reporting for specific materials, systems and methods

We require information from authors about some types of materials, experimental systems and methods used in many studies. Here, indicate whether each material, system or method listed is relevant to your study. If you are not sure if a list item applies to your research, read the appropriate section before selecting a response.

Materials & experimental systems

n/a	Involved in the study
☒	☐ Antibodies
☒	☐ Eukaryotic cell lines
☒	☐ Palaeontology and archaeology
☒	☐ Animals and other organisms
☒	☐ Clinical data
☒	☐ Dual use research of concern

Methods

n/a	Involved in the study
☒	☐ ChIP-seq
☒	☐ Flow cytometry
☒	☐ MRI-based neuroimaging
